# Improving outcomes in donation after circulatory death vs. donation after brain death liver transplantation for acute-on-chronic liver failure

**DOI:** 10.3389/ti.2026.16308

**Published:** 2026-08-21

**Authors:** Yvette Ysabel Yao, Tianyi Wen, Nicholas Wellington Tjandra, Kirby Lau, Yi Chun Lin, Xuan Chen, Daljeet Chahal

**Affiliations:** 1 Department of Internal Medicine, The University of British Columbia, Vancouver, BC, Canada; 2 Division of Gastroenterology, Vancouver General Hospital, University of British Columbia, Vancouver, BC, Canada

**Keywords:** graft failure, graft survival, liver grafts, liver transplant, survival analysis

## Abstract

Acute-on-chronic liver failure (ACLF) carries high short-term mortality, and liver transplantation (LT) remains the curative treatment. Donation after circulatory death (DCD) grafts expand the donor pool but carry higher risks of dysfunction compared to donation after brain death (DBD) grafts, and data directly comparing their outcomes in ACLF are limited. Using the Scientific Registry of Transplant Recipients (SRTR), we conducted a retrospective cohort study of 67,201 adult LT recipients from 2004 to 2023, stratified into three eras (Era 1: 2004–2012, Era 2: 2013–2018, Era 3: 2019–2023). Patients with prior LT, fulminant liver failure (status 1A), active hepatocellular carcinoma, or multivisceral transplants (excluding liver-kidney) were excluded. Estimated ACLF (EST-ACLF) severity was defined using EASL-CLIF criteria. One-year graft failure and survival were assessed using multivariable Cox regression and Kaplan-Meier analyses. Of 67,201 recipients, 62,510 received DBD and 4,691 DCD grafts. DCD grafts did not show clinically meaningful differences in 1-year hazards of graft failure compared to DBD grafts. One-year graft survival in EST-ACLF-2/3 improved over time from 87.2% in Era 1%–96.0% in Era 3, approaching the 97.5% observed with DBD grafts. DCD grafts may represent a viable and increasingly safe strategy for expanding LT access in ACLF.

## Graphical Abstract

**Figure:**
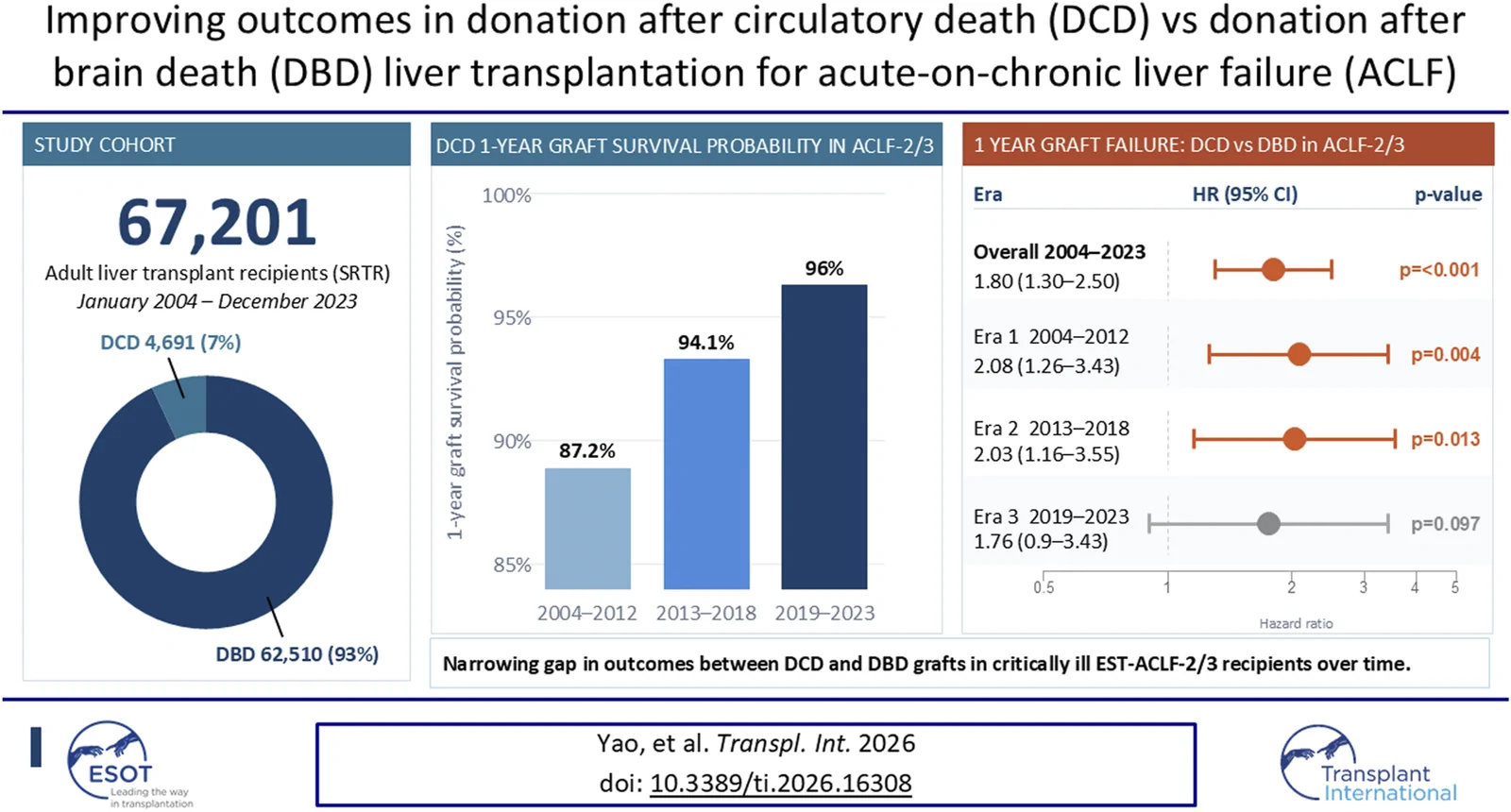

## Introduction

Acute-on-chronic liver failure (ACLF) is a systemic syndrome with high 28-day mortality characterized by acute hepatic deterioration and extrahepatic organ failure (OF) in patients with cirrhosis or chronic liver disease [1–3]. ACLF severity is commonly graded by the European Association for the Study of the Liver–Chronic Liver Failure (EASL-CLIF) consortium criteria, which is based on the number of OFs [4]. ACLF grade 3 (ACLF-3), defined as three or more OFs, has a poor prognosis with 28-day mortality of up to 80% for those who do not receive a liver transplant (LT) [5–7]. Improving access to LT is the only option for long-term survival [8–10].

In the context of donor liver shortages, donation after circulatory death (DCD) grafts have played a role in increasing the availability of LT organs and improving waitlist outcomes [11]. However, the utility of DCD grafts is controversial [12, 13]. DCD liver grafts are associated with higher rates of early allograft dysfunction and ischemic cholangiopathy compared to donation after brain death (DBD) grafts, but patient-centered outcomes such as days alive and out of hospital, graft failure, re-transplantation, and 1-year mortality are similar between the two groups [14–16]. DCD grafts are more likely to be discarded, and DCD recipients have a higher risk of graft loss compared to DBD recipients [17, 18]. Five-year mortality is modestly higher for DCD recipients, but survival remains acceptable [11]. DCD livers remain underutilized in the United States (US), with significant center-level variation, although high-utilization centers achieve similar 1-year patient and graft survival as low-utilization centers, suggesting that careful selection and center experience mitigate risk [19].

The use of DCD grafts may be the most feasible option to ensure timely LT, especially in ACLF-3 patients with high waitlist mortality [20]. The current system of allocation based on the model of end-stage liver disease (MELD) or MELD-Na may disadvantage ACLF patients by failing to capture extrahepatic OFs [21]. In a recent analysis of the Organ Procurement and Transplantation Network and United Network for Organ Sharing (OPTN/UNOS), Kitajima et al. found that DCD grafts in patients with ACLF were a risk factor for patient death within 1 year [22]. However, these studies did not directly compare outcomes between DCD and DBD grafts in patients with ACLF.

To our knowledge, there are no studies that directly compare DCD to DBD grafts in ACLF. Examining the utility of DCD grafts for ACLF is important for expanding the donor pool and improving waitlist mortality [20]. This is relevant with the advent of perfusion technologies which may significantly increase the utility of DCD grafts [23]. This study aimed to investigate 1-year post-LT outcomes in ACLF, specifically in graft failure and survival probability, after the use of DCD compared to DBD grafts.

## Materials and methods

### Ethics

The study was deemed as exempt from review by the institutional review board. The study followed the STROBE (STrengthening the Reporting of OBservational studies in Epidemiology) guidelines.

### Patient population

We used data from the Scientific Registry of Transplant Recipients (SRTR) collected mostly by the OPTN, which captures all patients who received an LT in the US [24].

### Inclusion/exclusion criteria

**FIGURE 1:**
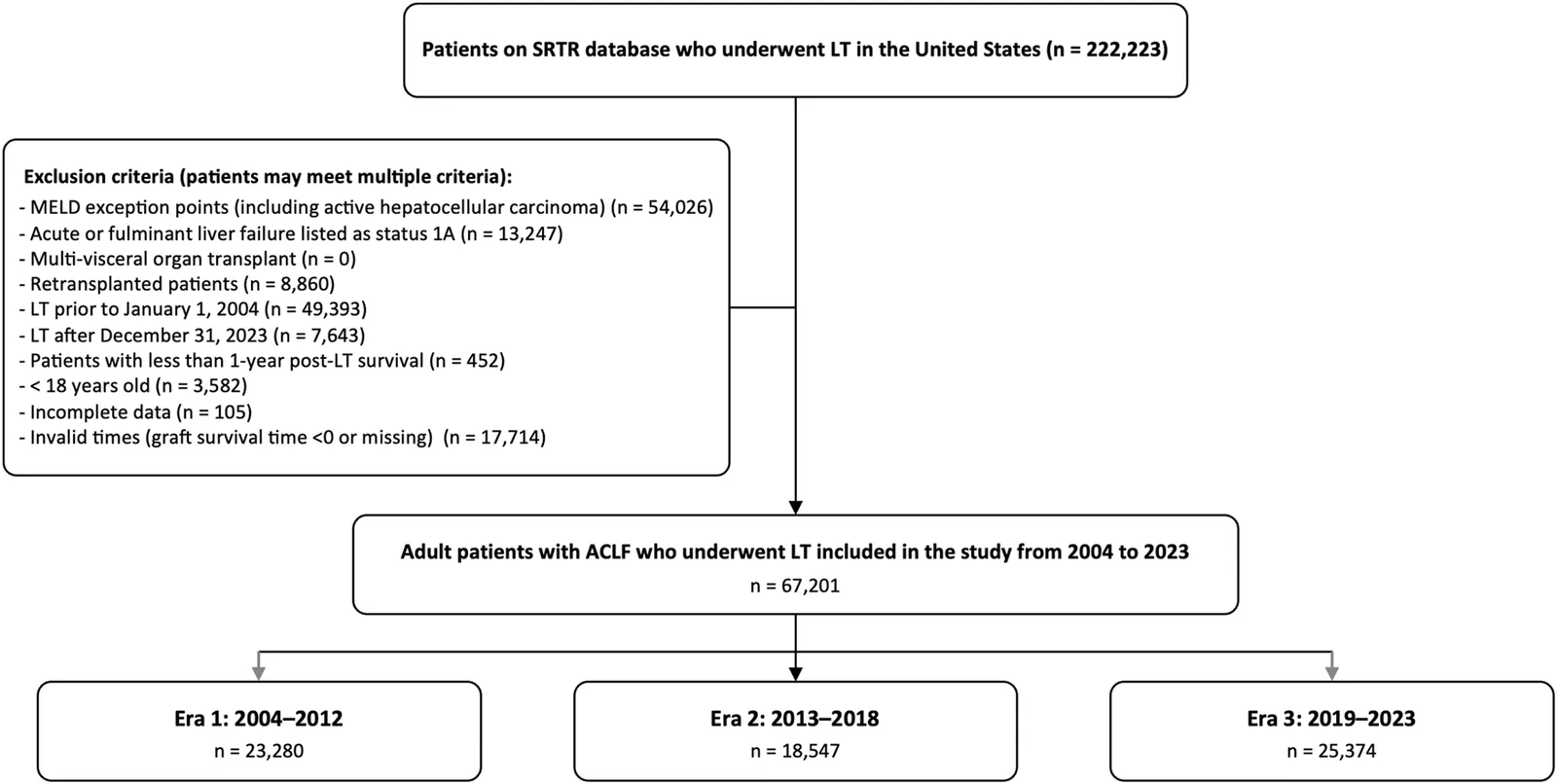
Flow chart of study population selection.

Adult patients (≥18 years old) listed for LT from 1st January 2004 to 31st December 2023 were evaluated. Patients with acute or fulminant liver failure (status 1A), prior LT, or active hepatocellular carcinoma at time of waitlist registration were excluded. Patients who underwent multivisceral organ transplant, except for simultaneous liver-kidney transplant were also excluded. We collected data on patient and donor characteristics at time of transplant and at regular follow-up intervals including age, gender, and race/ethnicity. Donor characteristics also included cause of death, history of diabetes, history of hypertension, and high-risk donor status. Recipient characteristics also included last bilirubin, INR, creatinine, encephalopathy, and ventilator use for life support. Only patients with at least 1 year of post-LT survival were included. The flow chart of study population selection is shown in Figure 1.

### Creation of cohorts

ACLF severity was graded using the EASL-CLIF criteria based on the number of OFs at time of LT across five domains: hepatic failure (bilirubin ≥12 mg/dL), coagulopathy (INR ≥2.5), renal failure (creatinine ≥2 mg/dL or dialysis within the preceding week), cerebral failure (hepatic encephalopathy), and respiratory failure (mechanical ventilation) [25]. Given inherent issues with the SRTR coding of vasopressor use, circulatory failure was not included. Since the SRTR dataset lacks sufficient detail to definitively diagnose ACLF, we used the term estimated ACLF (EST-ACLF). Patients were categorized into three grades: EST-ACLF-1 (1 OF), EST-ACLF-2 (2 OFs) and EST-ACLF-3 (≥3 OFs). Lower ACLF severity patients were grouped to avoid artificially excluding patients whose OF count at transplant may have been transiently lower. Non-critically ill patients were grouped as having a maximum of one OF (No ACLF or EST-ACLF-1), and critically ill patients were grouped as having two or more OFs (EST-ACLF-2, EST-ACLF-3).

We then stratified patients as either DBD or DCD graft recipients. This resulted in four cohorts: 1) Non-critically ill DBD recipients, 2) Non-critically ill DCD recipients, 3) Critically ill DBD recipients, and 4) Critically ill DCD recipients. Non-critically ill patients served as a comparator group for contextualising DCD outcomes across illness severity.

### Era analysis

Trends were evaluated over time based on three eras: Era 1 (2004–2012), Era 2 (2013–2018), and Era 3 (2019–2023). To address whether recipient factors were independently associated with 1-year graft failure in Era 3, we performed a sensitivity Cox proportional hazards model. This included donor type, MELD, and recipient age. Pretransplant diabetes and drug-treated hypertension were excluded because extensive missing or blank coding produced an unstable complete-case model with very few events.

### Data preparation and variable selection

Full details of data preparation, missingness and imputation, variable selection, model selection and model validation are provided in the Supplementary Material. From 128 clinically preselected predictors, 10-fold cross-validated Cox-LASSO retained 13 variables with non-zero coefficients at the one-standard-error penalty (λ_1se_ = 0.0037; Supplementary Figure SA1). Clinical review subsequently excluded nine variables with non-intuitive, implausible or unstable directions of effect. Specifically, variables regarding recipient or graft status at follow-up, recipient hepatic artery thrombosis or rejection, were excluded as post-transplant adverse events that biased results whereas our focus was on the identification of pre-transplant risk factors. Covariates with counter-intuitive effects, including recipient immunosuppression, recipient race and recipient ventilator status, were excluded to ensure model stability and interpretability. This left four covariates for multivariable modeling: donor type/ACLF stratum (reference: DBD with EST-ACLF-2/3, representing higher-risk DBD cases), donor diabetes, donor hypertension, and standardized donor age (interpreted per 10-year increase).

### Statistical analysis

The primary endpoint was 1-year post-transplant graft failure, defined as retransplantation or graft loss excluding death. This was expressed as HRs with 95% confidence intervals (CIs). Graft survival was estimated using Kaplan–Meier methods, with between-group differences assessed by log-rank test. Collinearity was evaluated using variance inflation factors. Model fit and proportional-hazards assumptions were determined using Schoenfeld residuals. Continuous variables were summarized as medians with interquartile ranges, and categorical variables as frequencies and percentages. Missing values were imputed using the median for continuous variables and the mode for categorical variables. Group comparisons were conducted using chi-squared tests for categorical variables and analysis of variance or Kruskal–Wallis tests for continuous variables. Significance was defined as *p* < 0.05.

We applied an exploratory minimum clinically important difference (MCID) framework using a distribution-based approach to assess whether statistically significant differences were clinically meaningful. Since death with a functioning graft precludes subsequent graft failure, we performed a competing-risk sensitivity analysis in which death with a functioning graft was treated as a competing event rather than as censoring alone. Subdistribution hazard ratios (SHR) were estimated using Fine-Gray regression models, allowing the association between donor-recipient strata and graft failure to be re-evaluated while explicitly accounting for the competing risk of death. Statistical analyses were performed using RStudio Version 2025.05.1 + 513 (Boston, MA, USA).

## Results

### Study population characteristics

67,201 adult liver-only transplant recipients with ACLF were included between January 1, 2004 and December 31, 2023 (Supplementary Figure SA1). Of these, 62,510 (93%) received DBD grafts and 4,691 (7%) received DCD grafts.

#### Donor characteristics

Donor characteristics are summarized in Table 1. Compared with DBD donors, DCD donors were younger (median 38 years [IQR 27–49] vs. 41 years [IQR 28–54]; p < 0.001), more frequently male (67% vs. 60%; p < 0.001), and more often White (87% vs. 78%, p < 0.001). Anoxia was a more frequent cause of death among DCD donors (50% vs. 33%), whereas cerebrovascular/stroke (17% vs. 33%) and head trauma (28% vs. 31%) were less common compared with DBD donors (p < 0.001). Compared to DBD donors, more DCD donors did not have diabetes (91% vs. 87%, p < 0.001) and fewer had hypertension (27% vs. 35%; p < 0.001). More DCD donors met CDC high-risk donor status (20%) than DBD donors (19%) (p < 0.001). Donor risk index was higher among DCD than DBD donors (2.35 [IQR 2.09–2.73] vs.1.65 [IQR 1.45–1.95]; p < 0.001).

**TABLE 1:** Donor and recipient characteristics by donor type for patients with ACLF that received a LT between 1 January 2004 and 31 December 2023, n = 67,201.

Variable	Outcome	Overall	DBD	DCD	P-value
Total n, (%)	​	67,201	62,510 (93%)	4,691 (7%)	​
1-year graft failure n, (%)	​	7131	6,366 (89%)	765 (11%)	​
Donor characteristics					
Age at organ recovery/referral years median [IQR]	​	41.00 (28.00, 54.00)	41.00 (28.00, 54.00)	38.00 (27.00, 49.00)	<0.001
Gender n, (%)	Female	26,248 (39%)	24,721 (40%)	1,527 (33%)	<0.001
	Male	40,953 (61%)	37,789 (60%)	3,164 (67%)	​
Race n, (%)	Asian	1,542 (2.3%)	1,458 (2.3%)	84 (1.8%)	<0.001
	Black	12,237 (18%)	11,762 (19%)	475 (10%)	​
	Multi	205 (0.3%)	192 (0.3%)	13 (0.3%)	​
	Native	357 (0.5%)	325 (0.5%)	32 (0.7%)	​
	Pacific	166 (0.2%)	157 (0.3%)	9 (0.2%)	​
	White	52,685 (78%)	48,610 (78%)	4,075 (87%)	​
Cause of death n, (%)	Anoxia	22,913 (34%)	20,552 (33%)	2,361 (50%)	<0.001
	Cerebrovascular/stroke	21,658 (32%)	20,850 (33%)	808 (17%)	​
	Head trauma	20,959 (31%)	19,635 (31%)	1,324 (28%)	​
	CNS tumor	270 (0.4%)	266 (0.4%)	4 (<0.1%)	​
	Other	1,400 (2.1%)	1,206 (1.9%)	194 (4.1%)	​
Diabetes n, (%)	Duration unknown	866 (1.3%)	824 (1.3%)	42 (0.9%)	<0.001
	0–5 years	2,526 (3.8%)	2,372 (3.8%)	154 (3.3%)	​
	6–10 years	1,408 (2.1%)	1,343 (2.1%)	65 (1.4%)	​
	>10 years	2,878 (4.3%)	2,737 (4.4%)	141 (3.0%)	​
	None	58,883 (88%)	54,629 (87%)	4,254 (91%)	​
	N/A	639 (1.0%)	604 (1.0%)	35 (0.7%)	​
Hypertension n, (%)	Yes	23,209 (35%)	21,967 (35%)	1,242 (27%)	<0.001
Meets CDC guidelines for high risk donor n, (%)	Yes	12,636 (19%)	11,693 (19%)	943 (20%)	<0.001
	No	53,451 (80%)	49,704 (80%)	3,747 (80%)	​
	Unknown	86 (0.1%)	85 (0.1%)	1 (<0.1%)	​
	N/A	1,028 (1.5%)	1,028 (1.6%)	0 (0%)	​
Donor risk index (DRI)	​	1.65 (1.45, 1.95)	2.35 (2.09, 2.73)	<0.001	1.65 (1.45, 1.95)
Recipient characteristics					
Age at treatment (years) median [IQR]	​	54.00 (47.00, 61.00)	54.00 (47.00, 61.00)	56.00 (49.00, 62.00)	<0.001
Age at listing (years) median [IQR]	​	54.00 (46.00, 60.00)	54.00 (46.00, 60.00)	56.00 (49.00, 62.00)	<0.001
Gender n, (%)	Female	24,557 (37%)	22,889 (37%)	1,668 (36%)	0.15
	Male	42,644 (63%)	39,621 (63%)	3,023 (64%)	​
Race n, (%)	Asian	2,023 (3.0%)	1,458 (2.3%)	84 (1.8%)	<0.001
	Black	5,579 (8.3%)	11,762 (19%)	478 (10%)	​
	Multi	339 (0.5%)	192 (0.3%)	13 (0.3%)	​
	Native	595 (0.9%)	331 (0.5%)	32 (0.7%)	​
	Pacific	101 (0.2%)	157 (0.3%)	9 (0.2%)	​
	White	58,537 (87%)	48,610 (78%)	4,075 (87%)	​
MELD median [IQR]	​	23 (16.60, 31.59)	24 (16.97, 32.10)	17 (13.54, 22.46)	<0.001
Last bilirubin (used for MELD) median [IQR]	​	7.30 (3.30, 18.00)	7.80 (3.40, 18.90)	3.90 (2.20, 7.40)	<0.001
Last INR (used for MELD) median [IQR]	​	2.00 (1.54, 2.60)	2.00 (1.60, 2.60)	1.70 (1.40, 2.10)	<0.001
Last serum creatinine mg/dL (used for MELD) median [IQR]	​	1.15 (0.82, 1.80)	1.18 (0.83, 1.90)	1.00 (0.77, 1.30)	<0.001
Last encephalopathy (used for MELD) n, (%)	None	18,640 (28%)	17,311 (28%)	1,329 (28%)	<0.001
	1–2	36,447 (54%)	33,713 (54%)	2,734 (58%)	​
	3–4	12,110 (18%)	11,482 (18%)	628 (13%)	​
	N/A	4 (<0.1%)	4 (<0.1%)	0 (0%)	​
Ventilator n, (%)	​	3,519 (5.2%)	3,434 (5.5%)	85 (1.8%)	<0.001

Abbreviations: DBD, donation after brain death; DCD, donation after circulatory death.

#### Recipient characteristics

Recipient characteristics are summarized in Table 1. Compared with DBD recipients, DCD recipients were older at treatment (median 56 years [IQR 49–62] vs. 54 years [IQR 47–61]; p < 0.001) and listing (median 56 years [IQR 49–62] vs. 54 years [IQR 46–60]; p < 0.001). Gender distribution was similar. DCD recipients were more frequently White (91% vs. 87%, p < 0.001). DCD recipients had a significantly lower MELD score than DBD recipients (median 17 vs. 24, p < 0.001).

DCD recipients were less ill at transplant with lower median bilirubin (3.90 mg/dL [IQR 2.20–7.40] vs. 7.80 mg/dL [IQR 3.40–18.90]; p < 0.001), lower INR (1.70 [IQR 1.40–2.10] vs. 2.00 [IQR 1.60–2.60]; p < 0.001), and lower creatinine (1.00 mg/dL [IQR 0.77–1.30] vs. 1.18 mg/dL [IQR 0.83–1.90]; p < 0.001) compared with DBD recipients. More DCD recipients had grade 1–2 encephalopathy (58% vs. 54%) and less had grades 3–4 (13% vs. 18%) (p < 0.001). Ventilator use was less frequent among DCD recipients (1.8% vs. 5.5%; p < 0.001).

Sensitivity Cox proportional hazards model was performed on LT recipients in Era 3 to see whether recipient factors were associated with 1-year graft failure. The recipient-factor model included 25,374 recipients and 698 1-year graft-failure events. After adjustment for MELD and recipient age, DCD donor type remained independently associated with higher 1-year graft failure (HR 2.00, 95% CI 1.65–2.42; p < 0.001). MELD was not significantly associated with 1-year graft failure (HR 0.99, 95% CI 0.98–1.00; p = 0.06). Recipient age showed a small association (HR 0.99, 95% CI 0.99–1.00; p = 0.03).

#### DCD-specific graft complications

Biliary tract complications (20% vs. 11%; p < 0.001) and infection (28% vs. 21%; p = 0.03) were more frequently reported causes of graft failure in DCD than DBD recipients. Conversely, primary graft failure (8.9% vs. 15%; p = 0.04) and recurrent hepatitis (15% vs. 31%; p < 0.001) were less frequent, and vascular thrombosis was numerically lower (4.1% vs. 7.7%; p = 0.11) in DCD versus DBD grafts. *De novo* hepatitis, recurrent non-hepatitis disease, acute and chronic rejection did not differ significantly between DCD and DBD graft complications. Cause indicators were non-mutually exclusive fields. Thus, individual patients could have more than one cause attributed.

### Hazard ratios of one-year graft failure over eras

#### DCD grafts

Compared with the critically ill DBD EST-ACLF-2/3 reference group, critically ill DCD recipients had significantly higher hazards of graft failure overall (HR 1.80, 95% CI 1.30–2.50, *p* < 0.001), with consistent associations in Era 1 (HR 2.08, 95% CI 1.26–3.43, *p* = 0.004) and Era 2 (HR 2.03, 95% CI 1.16–3.55, *p* = 0.013) (Table 2). This effect was attenuated and nonsignificant in Era 3 (HR 1.76, 95% CI 0.9–3.43, *p* = 0.097) (Table 2). Similarly, non-critically ill DCD recipients had an increased risk of graft failure overall (HR 1.42, 95% CI 1.23–1.64, *p* < 0.001) and across all eras, ranging from HR 1.38 (95% CI 1–1.91, *p* = 0.05) in Era 1 to HR 2.11 (95% CI 1.71–2.6, *p* < 0.001) in Era 3 (Table 2).

**TABLE 2:** Multivariable stratified Cox proportional hazards regression model for 1-year graft failure in each ACLF cohort severity overall and according to eras.

​	Overall: 2004–2023			Era 1: 2004–2012			Era 2: 2013–2018			Era 3: 2019–2023		
Variable	HR (95% CI)	p	VIF	HR (95% CI)	p	VIF	HR (95% CI)	p	VIF	HR (95% CI)	p	VIF
DBD												
EST-ACLF-2 and 3	Referent	​	1.01	Referent	​	1	Referent	​	1.01	Referent	​	1.01
No ACLF and EST-ACLF-1	0.93 (0.86–1.01)	0.08	​	0.82 (0.74–0.91)	<0.001	​	0.84 (0.71–0.99)	0.043	​	0.92 (0.77–1.09)	0.321	​
DCD												
EST-ACLF-2 and 3	1.80 (1.30–2.50)	<0.001	​	2.08 (1.26–3.43)	0.004	​	2.03 (1.16–3.55)	0.013	​	1.76 (0.9–3.43)	0.097	​
No ACLF and EST-ACLF-1	1.42 (1.23–1.64)	<0.001	​	1.38 (1–1.91)	0.05	​	1.88 (1.4–2.51)	<0.001	​	2.11 (1.71–2.6)	<0.001	​
Donor characteristics												
Diabetes	1.30 (1.18–1.44)	<0.001	1.07	1.33 (1.15–1.54)	<0.001	1.07	1.45 (1.17–1.81)	<0.001	1.08	1.46 (1.2–1.77)	<0.001	1.08
Hypertension	1.21 (1.11–1.33)	<0.001	1.2	1.08 (0.96–1.22)	0.194	1.2	1.22 (1–1.48)	0.046	1.21	1.49 (1.24–1.78)	<0.001	1.19
Age	1.01 (1.01–1.01)	<0.001	1.18	1.01 (1.01–1.02)	<0.001	1.18	1.01 (1–1.01)	0.009	1.2	1 (1–1.01)	0.321	1.19

The reference category was DBD with EST-ACLF-2 or 3. Bold type indicates statistically significant difference. Abbreviations: DBD, donation after brain death; DCD, donation after circulatory death; ACLF, acute-on-chronic liver failure; HR, hazard ratio; CI, confidence interval; VIF, variable inflation factors.

#### DBD grafts

DBD grafts in non-critically ill recipients were not associated with graft failure compared to the critically ill DBD reference group overall (HR 0.93, 95% CI 0.86–1.01, *p* = 0.08) (Table 2). DBD grafts in non-critically ill patients had a lower risk of 1-year graft failure compared to DBD grafts in more critically ill patients in Era 1 (HR 0.82, 95% CI 0.74–0.91, *p* < 0.001) and Era 2 (HR 0.84, 95% CI 0.71–0.99, *p* = 0.043), but this was not significant in Era 3.

#### Donor comorbidities

Donor diabetes was associated with increased graft failure risk overall (HR 1.30, 95% CI 1.18–1.44, *p* < 0.001), particularly in Era 2 (HR 1.45, 95% CI 1.17–1.81, *p* < 0.001) and Era 3 (HR 1.46, 95% CI 1.2–1.77, *p* < 0.001) (Table 2). Donor hypertension was also associated with higher hazards overall (HR 1.21, 95% CI 1.11–1.33, *p* < 0.001) and in most eras (Table 2). Increasing donor age was associated with a modest increase in graft failure risk overall (HR 1.01 per year, 95% CI 1.01–1.01, *p* < 0.001), particularly in Era 1 and Era 2 (Table 2).

### Survival analysis over eras

**FIGURE 2:**
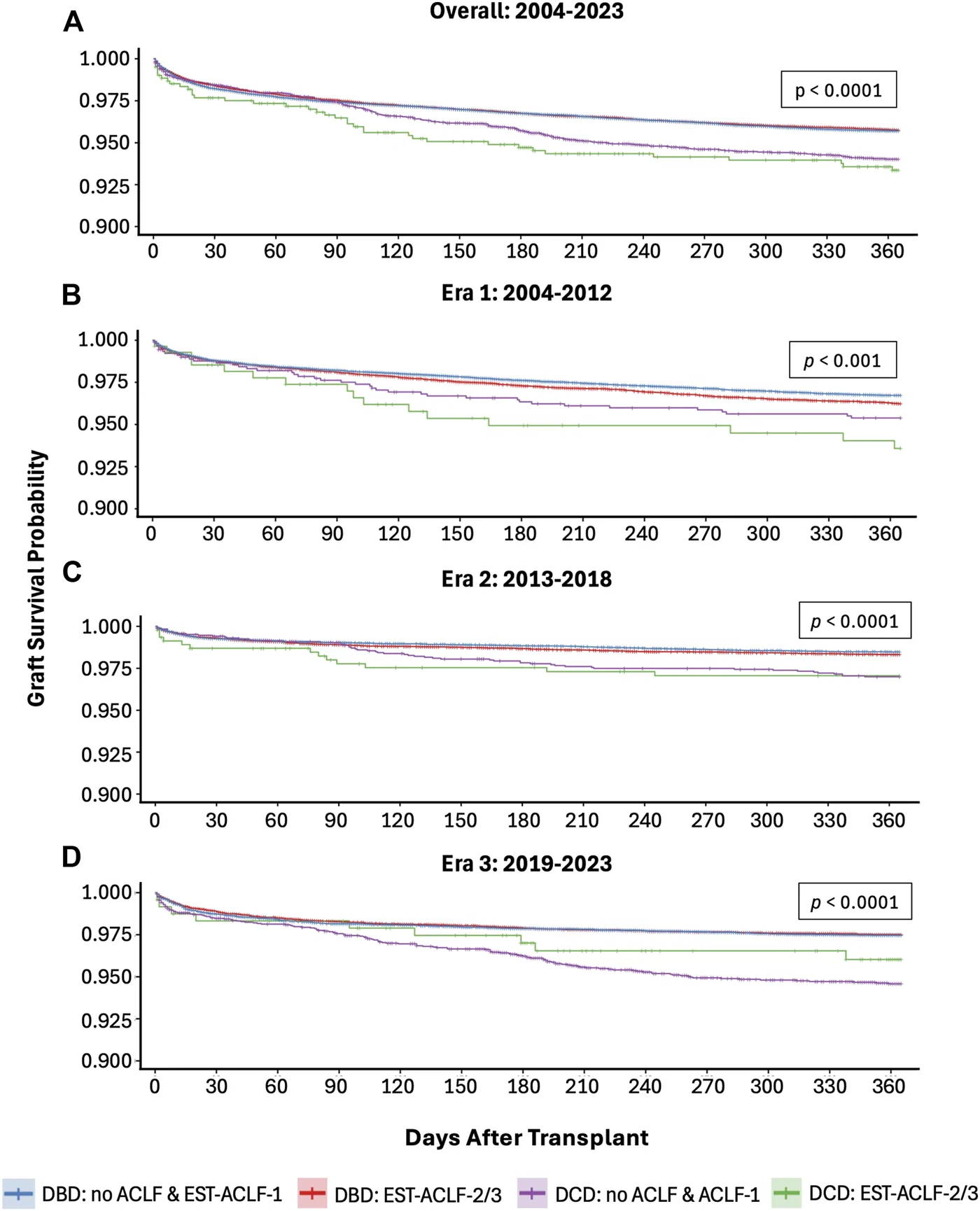
Kaplan-Meier 1-year graft survival curves stratified by eras and estimated ACLF (EST-ACLF) severity. **(A)** Overall: 2004–2023, **(B)** Era 1 (2004–2012), **(C)** Era 2 (2013–2018), **(D)** Era 3 (2019–2023). P values were calculated by log-rank test. Hazard ratios and corresponding confidence intervals are further detailed in Table 2.

Cox regression findings were consistent with Kaplan-Meier analysis (Figure 2) which showed significant differences in 1-year graft survival between groups. Across all eras, 1-year graft survival was significantly lower among DCD recipients compared to DBD counterparts although absolute differences were small (Figure 2). DCD recipients with EST-ACLF-2/3 had the lowest graft survival at 93.4% compared to other groups (Figure 2A). One-year graft survival probabilities for DBD and no ACLF/EST-ALCF-1 increased over time from 93.4% (Era 1), 97% (Era 2), and 97.5% (Era 3). A similar increase was seen for DBD EST-ACLF-2/3 with 92.5% (Era 1), 96.6% (Era 2), 97.5% (Era 3), respectively (p < 0.0001). There were no significant differences between the survival probabilities of critically ill and non-critically ill DCD recipients. For patients with DCD EST-ACLF-2/3 recipients, 1-year graft survival increased from 87.2% in Era 1, 94.1% in Era 2, and 96% in Era 3 respectively. For DCD recipients with no ACLF/EST-ACLF-1, 1-year graft survival also increased from 90.8% in Era 1, 94% in Era 2, and 94.6% in Era 3.

### Effect-size, competing-risk, and fine-gray analyses

Overall, 1-year Kaplan-Meier graft survival was 95.72% for DBD and 93.94% for DCD grafts. Across the eras, 1-year graft survival was 93.13% versus 89.97% in 2004–2012, 96.81% versus 94.00% in 2013–2018, and 97.48% versus 94.70% in 2019–2023 for DBD and DCD grafts, respectively. Across donor-ACLF strata, 1-year graft survival in the most recent era remained high in all groups, ranging from 94.58% to 97.51%, with the lowest estimates again observed among DCD recipients.

Effect-size analyses placed these differences in clearer clinical context. For DCD versus DBD, the absolute increase in 1-year graft failure was 1.66 percentage points (95% CI, 0.97–2.35) in the overall cohort, 2.94 percentage points (95% CI, 0.54–5.34) in 2004–2012, 2.68 percentage points (95% CI, 1.32–4.04) in 2013–2018, and 2.60 percentage points (95% CI, 1.78–3.42) in 2019–2023. By contrast, among DBD recipients, higher ACLF severity was associated with little to no absolute difference in 1-year graft failure: the risk difference for DBD with EST-ACLF-2/3 versus DBD with no ACLF/EST-ACLF-1 was −0.12 percentage points (95% CI, −0.44 to 0.20) overall, 0.71 percentage points (95% CI, 0.00–1.41) in 2004–2012, 0.27 percentage points (95% CI, −0.25–0.79) in 2013–2018, and −0.06 percentage points (95% CI, −0.47 to 0.35) in 2019–2023. None of these differences exceeded the prespecified MCID thresholds, which ranged from 7.73% to 12.42% across eras, indicating that the clinical magnitude of the observed differences was modest relative to the strong absolute 1-year outcomes.

Competing-risk analyses showed that death with a functioning graft occurred more frequently than graft failure overall and in every era (5.74% versus 4.26% overall, 7.34% versus 6.68% in 2004–2012, 5.65% versus 3.28% in 2013–2018, and 4.33% versus 2.75% in 2019–2023). In the Fine-Gray models, DCD strata consistently showed higher SHR of graft failure than the reference DBD with EST-ACLF-2/3 group, particularly DCD with no ACLF/EST-ACLF-1, with SHR 1.448 (95% CI 1.257–1.669) overall, SHR 1.417 (95% CI 1.027–1.957) in 2004–2012, SHR 1.904 (95% CI 1.425–2.546) in 2013–2018, and SHR 2.127 (95% CI 1.724–2.624) in 2019–2023. DCD with EST-ACLF-2/3 also showed elevated risk overall and in the earlier eras, although the estimate for 2019–2023 was no longer statistically significant (SHR 1.750, 95% CI 0.899–3.407).

## Discussion

In this national retrospective analysis of 67,201 adult liver-only transplant recipients (62,510 DBD, 4,691 DCD) from the SRTR database, outcomes following DCD LT in patients with ACLF improved over time and became increasingly comparable to DBD LT. Among the most critically ill ACLF patients, 1-year DCD graft survival improved from 87.2% in Era 1 (2004–2012) to 96.0% in Era 3 (2019–2023), approaching the 97.5% 1-year graft survival seen for DBD counterparts. Although the overall adjusted HR favored DBD grafts (HR 1.80, 95% CI 1.30–2.50; p < 0.001), this result is likely driven by the large sample size. Near 90% and above survival probabilities were observed even in the highest-risk DCD group in recent years. All absolute graft failure risk differences fell below MCID thresholds indicating no clinically meaningful differences between DCD and DBD grafts. These findings support the use of DCD grafts as a viable option in ACLF.

Several factors may explain why DCD outcomes in critically ill ACLF recipients have improved over time. Normothermic machine perfusion (NMP) for LT was first performed in 2012 [26] and has since been increasingly adopted, with a sharp increase between 2021 and 2022 [27]. NMP has increasingly been used in higher-risk recipient-donor combinations, indicating growing confidence in applying this technology to sicker patients [27]. Use of NMP for DCD donation was associated with a lower hazard of graft failure [28]. We were unable to formally account for the use of NMP, thus its impact on our outcomes may be underestimated. Future studies examining NMP’s effect on DCD outcomes in ACLF are warranted, as is center level analysis to differentiate outcomes between low and high volume DCD programs.

The decision to allocate a DCD graft to a recipient with ACLF reflects complex clinical judgement involving graft scarcity, perceived recipient risk, center experience, and local allocation practices. Providers are guided by frameworks such as the International Liver Transplant Society consensus, which recommends caution when DCD livers are used in higher risk patients that meet specific criteria [29]. Consistent with these principles, DCD recipients in our study were systematically less ill at transplantation than their DBD counterparts with lower median MELD scores (17 vs. 24), less severe hepatic encephalopathy, and markedly lower rates of ventilator dependence (1.8% vs. 5.5%). This may reflect a pattern of preferential allocation of DCD grafts to more stable recipients. This is supported by single center studies employing rigorous donor-recipient matching that report no difference in mortality or graft loss at 3 years post-transplant, with DCD not emerging as a risk factor for patient or graft survival [30]. However, outcomes seem to differ when extended-criteria DCD donors are used which carry particularly poor outcomes, whereas non-extended-criteria DCD grafts achieve patient survival comparable to DBD transplantation [31]. We found that in the most recent era, the association between DCD donor type and 1-year graft failure persisted after accounting for recipient MELD and age. These findings underscore that recipient illness severity and donor organ quality must be considered when optimizing donor-recipient matching.

Among causes of graft failure, biliary tract complications accounted for a significantly greater proportion among DCD compared to DBD grafts (20% vs. 11%, p < 0.001). This is consistent with the known susceptibility of DCD grafts to ischemia–reperfusion injury and ischemic cholangiopathy, both consequences of the warm ischemic period [32, 33]. NMP mitigates these risks by restoring near-physiological perfusion prior to implantation, and its expanding use is likely a contributor to the era-over-era improvement [34, 35]. Even setting perfusion technology aside, accepting a DCD offer confers better overall survival for patients with advanced liver disease than remaining on the waitlist, a pertinent consideration given the high waitlist mortality in ACLF [36].

In contrast to DCD grafts, DBD graft outcomes were stable across ACLF severity strata and did not significantly differ from one another in either adjusted or unadjusted analyses. This reinforces that DBD grafts remain the benchmark against which DCD performance is measured, and it also underscores that the principal driver of between-group differences in our analysis was the donor type rather than recipient illness severity alone.

Beyond donor type, donor diabetes (HR 1.30, 95% CI 1.18–1.44) and hypertension (HR 1.21, 95% CI 1.11–1.33) were independent predictors of graft failure. Both comorbidities are core components of the metabolic syndrome and are recognized indicators of hepatic microvascular compromise and reduced regenerative capacity [37]. Donor diabetes has been associated with increased graft failure risk, particularly in recipients with MASH [38]. Similarly, donor hypertension has been linked to reduced graft and patient survival, with both the presence and duration of hypertension implicated in worse outcomes [39]. Donor metabolic comorbidities should be accounted for when utilizing DCD grafts in ACLF.

Donor age was also associated with increased graft failure risk (HR 1.01, 95% CI 1.01–1.01). This is consistent with previous studies which show that DCD liver grafts from older donors have a higher incidence of primary nonfunction and initial poor function [40]. This likely reflects the decline in hepatic regenerative capacity with age [41]. Transplantation from younger DCD donors (under 50 years) has been associated with better graft survival than from older DBD donors (over 60 years), suggesting that careful age-based selection of DCD donors may allow for expansion of DCD utilization without compromising outcomes [42]. The effect of donor age on graft failure was no longer statistically significant in the most recent eras which may reflect either evolving allocation strategies or the selective acceptance of older donors with favorable graft profiles.

Competing-risk analyses provided additional context. DCD strata consistently showed higher SHR of graft failure than the reference DBD EST-ACLF-2/3 group, particularly DCD with no ACLF/EST-ACLF-1. That the non-critically ill DCD group consistently fared worse in graft failure is counterintuitive. This may reflect suboptimal donor–recipient matching in patients perceived as “safe” enough to tolerate a less optimal organ, inadvertently raising their graft failure risk. The loss of statistical significance in the most recent era is consistent with the Kaplan-Meier curves that showed 1-year graft survival becoming increasingly comparable to DBD outcomes over time.

Strengths of our study include the large sample size and national scope, providing robust statistical power and generalizability. Combining Cox-LASSO with clinical expert review yielded a parsimonious, interpretable model with acceptable discrimination (C-index 0.71), good calibration, negligible multicollinearity and only mild non-proportionality.

This analysis has some limitations. The restriction to US data limits generalizability to international populations. We are also limited by the retrospective design of national registry analysis that, in turn, offers the benefit of a large sample size. We did not assess graft survival beyond 1 year, so applicability to later outcomes remains uncertain. Additionally, unmeasured confounders, notably the increasing adoption of NMP technologies likely contributed to the improvement in DCD outcomes [43]. However, we have controlled for other confounders. Lastly, the SRTR database does not provide information regarding antihypertensive or diabetic therapies of the donors, nor the characteristics of the liver graft. Nonetheless, the consistency of our findings across multiple statistical diagnostics, eras, and both adjusted and unadjusted analyses strengthens confidence in their validity [44].

Overall, 1-year graft survival after DCD LT in ACLF has improved substantially over time and is now approaching DBD outcomes. DCD grafts represent a viable means of expanding the donor pool and reducing waitlist mortality in this high-risk population, particularly as NMP technologies become more widely adopted. These findings underscore the importance of nuanced donor–recipient matching when considering DCD grafts in ACLF. Avoiding high-risk combinations, such as older DCD donors with metabolic comorbidities allocated to higher severity ACLF recipients is likely to optimize graft survival.

## Data Availability

The original contributions presented in the study are included in the article/Supplementary Material, further inquiries can be directed to the corresponding author.

## References

[1] Karvellas CJ Bajaj JS Kamath PS Napolitano L O’Leary JG Solà E AASLD practice guidance on acute-on-chronic liver failure and the management of critically ill patients with cirrhosis. Hepatology (2024) 79(6):1463–502. 10.1097/HEP.0000000000000671 37939273

[2] Moreau R Jalan R Gines P Pavesi M Angeli P Cordoba J Acute-on-Chronic liver failure is a distinct syndrome that develops in patients with acute decompensation of cirrhosis. Gastroenterology (2013) 144(7):1426–37.e9. 10.1053/j.gastro.2013.02.042 23474284

[3] Zaccherini G Weiss E Moreau R . Acute-on-chronic liver failure: definitions, pathophysiology and principles of treatment. JHEP Rep (2021) 3(1):100176. 10.1016/j.jhepr.2020.100176 33205036 PMC7652714

[4] Xia L Qiao Z Zhang Z Lv Z Tong H Tong Y Transplantation for EASL-CLIF and APASL acute-on-chronic liver failure (ACLF) patients: the TEA cohort to evaluate long-term post-transplant outcomes. eClinicalMedicine (2022) 49:49. 10.1016/j.eclinm.2022.101476 PMC9167862 35747194

[5] Arroyo V Moreau R Jalan R Ginès P . Acute-on-chronic liver failure: a new syndrome that will re-classify cirrhosis. J Hepatol (2015) 62(1):S131–43. 10.1016/j.jhep.2014.11.045 25920082

[6] Jalan R Saliba F Pavesi M Amoros A Moreau R Ginès P Development and validation of a prognostic score to predict mortality in patients with acute-on-chronic liver failure. J Hepatol (2014) 61(5):1038–47. 10.1016/j.jhep.2014.06.012 24950482

[7] Laici C Guizzardi C Morelli MC Vitale G Caraceni P Cescon M Predictive factors of inhospital mortality for ICU patients with acute-on-chronic liver failure undergoing liver transplantation. Eur J Gastroenterol Hepatol (2022) 34(9):967–74. 10.1097/MEG.0000000000002413 35913780

[8] Mita A Shimizu S Ichiyama T Yamamoto T Yamaguchi A Sonoda K Outcomes of critically ill patients with liver failure who require mechanical ventilation: a retrospective, single-center study. Health Sci Rep (2024) 7(3):e1926. 10.1002/hsr2.1926 38469112 PMC10925802

[9] Artru F Louvet A Ruiz I Levesque E Labreuche J Ursic-Bedoya J Liver transplantation in the most severely ill cirrhotic patients: a multicenter study in acute-on-chronic liver failure grade 3. J Hepatol (2017) 67(4):708–15. 10.1016/j.jhep.2017.06.009 28645736

[10] Della Guardia B Boteon APCS Matielo CEL Felga G Boteon YL . Current and future perspectives on acute-on-chronic liver failure: challenges of transplantation, machine perfusion, and beyond. World J Gastroenterol (2022) 28(48):6922–34. 10.3748/wjg.v28.i48.6922 36632319 PMC9827581

[11] Kwon JH Blanding WM Shorbaji K Scalea JR Gibney BC Baliga PK Waitlist and transplant outcomes in organ donation after circulatory death: trends in the United States. Ann Surg (2023) 278(4):609–20. 10.1097/SLA.0000000000005947 37334722

[12] Mateo R Cho Y Singh G Stapfer M Donovan J Kahn J Risk factors for graft survival after liver transplantation from donation after cardiac death donors: an analysis of OPTN/UNOS data. Am J Transpl (2006) 6(4):791–6. 10.1111/j.1600-6143.2006.01243.x 16539637

[13] Mathur AK Heimbach J Steffick DE Sonnenday CJ Goodrich NP Merion RM . Donation after cardiac death liver transplantation: predictors of outcome. Am J Transpl (2010) 10(11):2512–9. 10.1111/j.1600-6143.2010.03293.x 20977642

[14] Ziogas IA Kakos CD Esagian SM Skarentzos K Alexopoulos SP Shingina A Liver transplant after donation from controlled circulatory death *versus* brain death: a UNOS database analysis and publication bias adjusted meta-analysis. Clin Transpl (2022) 36(2):e14521. 10.1111/ctr.14521 34689372

[15] Frasco PE Mathur AK Chang YH Alvord JM Poterack KA Khurmi N Days alive and out of hospital after liver transplant: comparing a patient-centered outcome between recipients of grafts from donation after circulatory and brain deaths. Am J Transpl Off J Am Soc Transpl Am Soc Transpl Surg (2023) 23(1):55–63. 10.1016/j.ajt.2022.10.007 36695622

[16] Limkemann AJ Singh N Helfrich K Schenk A Logan A Washburn L Safely expanding the liver donor pool by utilization of organs from donation after circulatory death with comparable results to donation after brain death, a large single-center experience. J Gastrointest Surg Off J Soc Surg Aliment Tract (2022) 26(7):1453–61. 10.1007/s11605-022-05313-0 PMC9012439 35428935

[17] Sher L Quintini C Fayek SA Abt P Lo M Yuk P Attitudes and barriers to the use of donation after cardiac death livers: comparison of a United States transplant center survey to the united network for organ sharing data. Liver Transpl Off Publ Am Assoc Study Liver Dis Int Liver Transpl Soc (2017) 23(11):1372–83. 10.1002/lt.24855 28834180

[18] Ruck JM Jackson KR Motter JD Massie AB Philosophe B Cameron AM Temporal trends in utilization and outcomes of DCD livers in the United States. Transplantation (2022) 106(3):543–51. 10.1097/TP.0000000000003878 34259435

[19] Manzi JLL Oliveira ES Rombach S Turra V Zaragoza S Bababekov Y Comparisons of liver transplant from DCD outcomes in high-utilization centers *versus* low-utilization centers in the US: a systematic review and meta-analysis. Front Immunol (2025) 16:1564551. 10.3389/fimmu.2025.1564551 40416988 PMC12098045

[20] Sundaram V Jalan R Wu T Volk ML Asrani SK Klein AS Factors associated with survival of patients with severe acute-on-chronic liver failure before and after liver transplantation. Gastroenterology (2019) 156(5):1381–91.e3. 10.1053/j.gastro.2018.12.007 30576643

[21] Karvellas CJ Francoz C Weiss E . Liver transplantation in acute-on-chronic liver failure. Transplantation (2021) 105(7):1471. 10.1097/TP.0000000000003550 33208692

[22] Kitajima T Kuno Y Ivanics T Lu M Moonka D Shimada S Improved survival with higher-risk donor grafts in liver transplant with acute-on-chronic liver failure. Transpl Direct (2022) 8(2):e1283. 10.1097/TXD.0000000000001283 35187210 PMC8806387

[23] Chahal D Hornby L Bird JD Lee SS Schiano TD Sekhon MS . Hepatic hypoxia in donation after circulatory death: physiology, clinical relevance, and future directions. Liver Transpl (2026) 32:315–23. 10.1097/LVT.0000000000000647 40454891

[24] The SRTR Database. (2025) Available online at: https://www.srtr.org/about-the-data/the-srtr-database/ (Accessed: August 12, 2025).

[25] Moreau R Tonon M Krag A Angeli P Berenguer M Berzigotti A EASL clinical Practice guidelines on acute-on-chronic liver failure. J Hepatol (2023) 79(2):461–91. 10.1016/j.jhep.2023.04.021 37364789

[26] op den Dries S Karimian N Sutton M Westerkamp A Nijsten M Gouw A *Ex vivo* normothermic machine perfusion and viability testing of discarded human donor livers. Am J Transpl (2013) 13(5):1327–35. 10.1111/ajt.12187 23463950

[27] Abu-Gazala S Tang H Abt P Mahmud N . National trends in utilization of normothermic machine perfusion in DCD liver transplantation. Transpl Direct (2024) 10(5):e1596. 10.1097/TXD.0000000000001596 38606351 PMC11005893

[28] Ibeabuchi T Li E Bittermann T Mahmud N Abt PL . Can *ex-situ* normothermic perfusion improve graft survival compared to static cold storage among donation after circulatory death liver allografts? Liver Transpl (2023) 29(9):952–60. 10.1097/LVT.0000000000000143 37016764 PMC11972138

[29] Schlegel A Foley DP Savier E Flores Carvalho M De Carlis L Heaton N Recommendations for donor and recipient selection and risk prediction: Working group report from the ILTS consensus conference in DCD liver transplantation. Transplantation (2021) 105(9):1892–903. 10.1097/TP.0000000000003825 34416750

[30] Hobeika MJ Saharia A Mobley CM Menser T Nguyen DT Graviss EA Donation after circulatory death liver transplantation: an in-depth analysis and propensity score-matched comparison. Clin Transpl (2021) 35(6):e14304. 10.1111/ctr.14304 33792971

[31] Pandya K Sastry V Panlilio MT Yip TCF Salimi S West C Differential impact of extended criteria donors after brain death or circulatory death in adult liver transplantation. Liver Transpl Off Publ Am Assoc Study Liver Dis Int Liver Transpl Soc (2020) 26(12):1603–17. 10.1002/lt.25859 32750732

[32] Foley DP Fernandez LA Leverson G Chin LT Krieger N Cooper JT Donation after cardiac death: the university of Wisconsin experience with liver transplantation. Ann Surg (2005) 242(5):724–31. 10.1097/01.sla.0000186178.07110.92 16244547 PMC1409855

[33] O’Neill S Roebuck A Khoo E Wigmore SJ Harrison EM . A meta-analysis and meta-regression of outcomes including biliary complications in donation after cardiac death liver transplantation. Transpl Int Off J Eur Soc Organ Transpl (2014) 27(11):1159–74. 10.1111/tri.12403 25052036

[34] Jassem W Xystrakis E Ghnewa YG Yuksel M Pop O Martinez‐Llordella M Normothermic machine perfusion (NMP) inhibits proinflammatory responses in the liver and promotes regeneration. Hepatology (2019) 70(2):682–95. 10.1002/hep.30475 30561835

[35] De Carlis R Muiesan P Taner B . Donation after circulatory death: novel strategies to improve the liver transplant outcome. J Hepatol (2023) 78(6):1169–80. 10.1016/j.jhep.2023.04.008 37208104

[36] Taylor R Allen E Richards JA Goh MA Neuberger J Collett D Survival advantage for patients accepting the offer of a circulatory death liver transplant. J Hepatol (2019) 70(5):855–65. 10.1016/j.jhep.2018.12.033 30639505

[37] Alberti KGMM Zimmet P Shaw J IDF Epidemiology Task Force Consensus Group. The metabolic syndrome--a new worldwide definition. Lancet Lond Engl (2005) 366(9491):1059–62. 10.1016/S0140-6736(05)67402-8 16182882

[38] Lim WH Ng CH Tan DJH Xiao J Fu CE Ong C Donor diabetes and steatosis affects recipient survival following liver transplantation based on etiology of liver cirrhosis. Transplantation (2024) 108(2):473–82. 10.1097/TP.0000000000004718 37439778

[39] Hu Z Mei S Xiang J Zhou J Li Z Zhou L Survival rates after liver transplantation using hypertensive donor grafts: an analysis of the scientific registry of transplant recipients database. J Hepato-biliary-pancreat Sci (2017) 24(8):441–8. 10.1002/jhbp.466 28544515

[40] Ploeg RJ D’Alessandro AM Knechtle SJ Stegall MD Pirsch JD Hoffmann RM Risk factors for primary dysfunction after liver transplantation--a multivariate analysis. Transplantation (1993) 55(4):807–13. 10.1097/00007890-199304000-00024 8475556

[41] Freeman RB . Deceased donor risk factors influencing liver transplant outcome. Transpl Int (2013) 26(5):463–70. 10.1111/tri.12071 23414069

[42] Scalea JR Redfield RR Foley DP . Liver transplant outcomes using ideal donation after circulatory death livers are superior to using older donation after brain death donor livers. Liver Transpl (2016) 22(9):1197–204. 10.1002/lt.24494 27314220

[43] Croome KP Subramanian V Mathur AK Aqel B Mao SA Clendenon JN Outcomes of DCD liver transplant using sequential normothermic regional perfusion and normothermic machine perfusion or NRP alone *versus* static cold storage. Transplantation (2025) 109(7):1184–90. 10.1097/TP.0000000000005301 39716357

[44] ODT Clinical - NHS Blood and Transplant. Donation after circulatory death. (2025) Available online at: https://www.odt.nhs.uk/deceased-donation/best-practice-guidance/donation-after-circulatory-death/ (Accessed: August 8, 2025).

