# Supplementary material for: Improving outcomes in donation after circulatory death vs. donation after brain death liver transplantation for acute-on-chronic liver failure
Source: Transpl Int. 2026 Aug 21;39:16308. doi: 10.3389/ti.2026.16308 (PMC13541644; doi:10.3389/ti.2026.16308)
Supplement: Supplementary file 1 [file Supplementaryfile1.docx]

**Capsule Sentence Summary**

In this large registry study, outcomes following DCD liver transplantation in acute-on-chronic liver failure have improved over time, approaching those of DBD grafts in the most recent era — offering optimism for expanding DCD utilization in this critically ill population.

**Supplementary Methods**

Variable Creation

We began by loading the primary transplant file (tx_li.sas7bdat) along with the MELD exception tables (mpexcept, mpexcept_orig_tumors, mpexcept_tumors) and the listing history file (stathist_liin) to apply our inclusion and exclusion criteria.

From df_tx_li, we deduplicated on PERS_ID and PX_ID, then excluded any patients with HCC exception points (CANHX_MPXCPT_HCC_APPLY_IND or CANHX_MPXCPT_HCC_APPROVE_IND), status‐1 listings (CAN_INIT_STAT in stathist_liin), non–liver transplants (ORG_TY != "LI"), prior retransplants (CAN_PREV_HL\HR\IN\KI\KP\LI\LUPA\TX), transplants outside January 1, 2004 to December 31, 2023, follow‐up under one year, pediatric patients (< 18 years at listing), or missing key labs (CAN_LAST_ENCEPH, REC_VENTILATOR, CAN_LAST_BILI, CAN_LAST_INR, CAN_LAST_SERUM_CREAT, CAN_LAST_DIAL_PRIOR_WEEK).

The resulting tx_li8_full dataset was used to define five organ‐failure indicators—hepatic (CAN_LAST_BILI ≥ 12), coagulation (CAN_LAST_INR ≥ 2.5), renal (CAN_LAST_SERUM_CREAT ≥ 2.0 or prior week dialysis), cerebral (CAN_LAST_ENCEPH > 1), and respiratory (REC_VENTILATOR == 1)—

which were summed into organ_failure_count and mapped to ACLF grades (No ACLF = 0 failures; ACLF 1 = 1; ACLF 2 = 2; ACLF 3 = 3; ACLF >3 = >3), with a binary has_aclf flag distinguishing any ACLF (grades 1–3) from none.

We then read the donor file (donor_deceased.sas7bdat), used the non‐NA DON_DCD_SUPPORT_WITHDRAW_DT field to classify donors as DCD versus DBD, and joined on DONOR_ID to append a donor_type variable. We chose the non-missing DON_DCD_SUPPORT_WITHDRAW_DT field to identify DCD donors because it directly reflects the controlled withdrawal of life-sustaining therapy, making it a clinically precise indicator; this field was present in 41 081(DCD) of 298 897 registry donors. When applied to our final analytic cohort of 67 244 adult, liver-only transplants, this approach yielded 62 548 (93.0 %) DBD and 4 696 (7.0 %) DCD classifications.

Finally, we created three strata definitions combining donor type and ACLF status: strata_group_1 as “donor_type and has_aclf,” strata_group_2 as “donor_type and aclf_category” with ACLF≥3 collapsed, and strata_group_3 as “donor_type and (No ACLF or ACLF1)” versus “donor_type and (ACLF2, ACLF3, or ACLF>3).”

After filtering to complete donor classifications, the final counts can be seen in Table 3 in the supplementary tables section.

Preparation for COX-LASSO Variable Selection

In preparation for Cox‐LASSO variable selection, we first constructed the necessary survival endpoints by calculating each patient’s time to graft failure or censoring, censoring at one year where appropriate, and excluded any records with non‐positive follow‐up times to ensure valid survival intervals.

Next, we identified our candidate covariates based on clinical expertise(Dr. Chahal selected like 128 variables), separating them into continuous and categorical lists for downstream processing.

To reduce bias from sparse data, we then removed any variables with more than 40 % missingness, followed by the exclusion of categorical predictors exhibiting over ten distinct levels to avoid overfitting and unstable parameter estimates.

We addressed remaining gaps by imputing missing continuous values with their median and filling missing categories with the most frequent level, thereby preserving sample size without introducing undue distortion.

Small categories within each factor that comprised less than ten percent of the largest group were collapsed into an “Other” category to strengthen analytic power and model convergence, and all categorical variables were then encoded as factors.

Before penalized fitting, we evaluated the skewness of each continuous covariate and log‐ or square‐root‐transformed those with absolute skewness above one to mitigate extreme tails and enhance linearity assumptions.

Finally, all transformed continuous predictors were standardized to a mean of zero and a standard deviation of one to place them on a common scale, ensuring fair penalty application and comparability across variables in the Cox‐LASSO procedure.

COX-LASSO Variable Selection

LASSO automatically tests each candidate predictor and keeps only those with a strong enough signal, those that most improve the model’s ability to predict one‐year graft failure, while discarding weaker or redundant variables.

To perform Cox–LASSO variable selection, we first constructed a design matrix of all standardized continuous covariates (continuous_vars_std) and dummy‐encoded categorical predictors and paired this with a one‐year survival outcome (time_to_event_1yr, event_1yr) in a Surv() object. We then fitted a penalized Cox model via cv.glmnet(..., family="cox", alpha=1, nfolds=10), tuning the L₁‐penalty λ over a logarithmic grid. Cross‐validated partial‐likelihood deviance was minimized at **λₘᵢₙ = 0.0015**, while the “one‐standard‐error” rule identified **λ₁ₛₑ = 0.0037**, which balanced fit and parsimony.

From 128 clinically preselected predictors, 10-fold cross-validated Cox-LASSO retained 13 variables with non-zero coefficients at the one-standard-error penalty (λ₁ₛₑ = 0.0037; Appendix Figure A2) : DON_HIST_DIAB, DON_NON_HR_BEAT, DON_HTN, DON_AGE, CAN_RACE_SRTR, REC_PX_STAT, REC_IMMUNO_MAINT_MEDS, REC_VENTILATOR, REC_GRAFT_STAT, REC_FAIL_REJ_ACUTE, REC_HEPATIC_ARTER_THROMB, TFL_LASTATUS, TFL_LASTATUS.

Covariates with counter-intuitive effects (DON_NON_HR_BEAT, REC_IMMUNO_MAINT_MEDS, CAN_RACE_SRTR and REC_VENTILATOR) were also excluded to ensure model stability and interpretability. The final multivariable Cox model therefore included only the combined donor‐type/ACLF strata (strata_group_3), donor diabetes history (DON_HIST_DIAB), donor hypertension (DON_HTN) and donor age (DON_AGE_trans), and all variance‐inflation factors were below 1.5, indicating no collinearity.

Donor age did not require transformation. Per our rule (transform only when |skewness| > 1), DON_AGE’s skewness was 0.159, so it was retained on its original scale. The variable labeled DON_AGE_trans in the Cox model is identical to DON_AGE (the ‘_trans’ suffix reflects preprocessing), and the hazard ratio is interpreted per 1-year increase in donor age.

**Figure A1. Cross-validation for Cox-Lasso Plot**

The cross‐validation plot (Figure 3) shows that deviance reaches its minimum around log(λ) ≈ –6.5 (λₘᵢₙ) and remains within one standard error at log(λ) ≈ –5.6 (λ₁ₛₑ), indicated by the two vertical dashed lines; the coefficient‐path plot illustrates how each predictor’s effect shrinks toward zero as λ increases, justifying our choice of the more parsimonious λ₁ₛₑ solution.

**Schoenfield Residual Test Statistics**

**Table A1.** “Schoenfeld Residual Test Statistics for Proportional-Hazards Assumption”

| **Variable** | **p** |
| --- | --- |
| **Donor Type & ACLF Strata** | **0.0081** |
| Donor diabetes | 0.8305 |
| Donor hypertension | 0.1728 |
| **Donor age (transformed)** | **0.0200** |
| **Global** | **0.0035** |

**Figure A2.** “Schoenfeld Residual Diagnostic Plots for Final Cox Model Covariates”

**Table A2.** “Schoenfeld Residual Test Statistics for Proportional-Hazards Assumption, 2018-2023”

| **Variable** | **p** |
| --- | --- |
| **Donor Type & ACLF Strata** | **<0.001** |
| Donor diabetes | 0.112 |
| Donor hypertension | 0.084 |
| Donor age (transformed) | 0.135 |
| **Global** | **<0.001** |

**Figure A3.** “Schoenfeld Residual Diagnostic Plots for 2018-2023 Cox Model Covariates”

**Table A3.** “Schoenfeld Residual Test Statistics for Proportional-Hazards Assumption, 2013-2023”

| **Variable** | **p** |
| --- | --- |
| **Donor Type & ACLF Strata** | **<0.001** |
| Donor diabetes | 0.244 |
| **Donor hypertension** | **0.045** |
| **Donor age (transformed)** | **0.020** |
| **Global** | **<0.001** |

**Figure A4.** “Schoenfeld Residual Diagnostic Plots for 2013-2023 Cox Model Covariates”

**Kaplan Meier Curves**

**Figure A5.** Kaplan Meier Curve for overall 2004-2023 study

**Figure A6.** Kaplan Meier Curve for 2004-2012 era

**Figure A7.** Kaplan Meier Curve for 2013-2018 era

**Figure A8.** Kaplan Meier Curve for 2019-2023 era

**Forest Plots: Hazard Ratios for 1-Year Graft Failure**

**Figure B1. 1-Year Graft Failure in 2004-2012**

**Figure B2. 1-Year Graft Failure in 2013-2018**

**Figure B3. 1-Year Graft Failure in 2019-2023**

**Figure B4. 1-Year Graft Failure in 2004-2023**

**Effect-Size, Competing-Risk, and Fine-Gray Analyses**

**Table C1. One-year Kaplan-Meier graft survival by donor type and donor-ACLF strata**

| **Period** | **Grouping** | **Group** | **N** | **1-year survival, %** | **95% CI lower** | **95% CI upper** |
| --- | --- | --- | --- | --- | --- | --- |
| Overall: 2004-2023 | Donor type | DBD | 62510 | 95.72 | 95.56 | 95.88 |
| Overall: 2004-2023 | Donor type | DCD | 4691 | 93.94 | 93.24 | 94.64 |
| Overall: 2004-2023 | Donor-ACLF strata | DBD and (ACLF 2,3,>3) | 24676 | 95.75 | 95.49 | 96.01 |
| Overall: 2004-2023 | Donor-ACLF strata | DBD and (No ACLF or ACLF1) | 37834 | 95.7 | 95.49 | 95.91 |
| Overall: 2004-2023 | Donor-ACLF strata | DCD and (ACLF 2,3,>3) | 610 | 93.37 | 91.35 | 95.43 |
| Overall: 2004-2023 | Donor-ACLF strata | DCD and (No ACLF or ACLF1) | 4081 | 94.02 | 93.28 | 94.77 |
| Era 1: 2004-2012 | Donor type | DBD | 22693 | 93.13 | 92.8 | 93.47 |
| Era 1: 2004-2012 | Donor type | DCD | 587 | 89.97 | 87.51 | 92.5 |
| Era 1: 2004-2012 | Donor-ACLF strata | DBD and (ACLF 2,3,>3) | 7332 | 92.45 | 91.83 | 93.08 |
| Era 1: 2004-2012 | Donor-ACLF strata | DBD and (No ACLF or ACLF1) | 15361 | 93.44 | 93.04 | 93.84 |
| Era 1: 2004-2012 | Donor-ACLF strata | DCD and (ACLF 2,3,>3) | 139 | 87.16 | 81.45 | 93.27 |
| Era 1: 2004-2012 | Donor-ACLF strata | DCD and (No ACLF or ACLF1) | 448 | 90.77 | 88.08 | 93.54 |
| Era 2: 2013-2018 | Donor type | DBD | 17373 | 96.81 | 96.55 | 97.08 |
| Era 2: 2013-2018 | Donor type | DCD | 1174 | 94 | 92.63 | 95.39 |
| Era 2: 2013-2018 | Donor-ACLF strata | DBD and (ACLF 2,3,>3) | 7832 | 96.63 | 96.23 | 97.04 |
| Era 2: 2013-2018 | Donor-ACLF strata | DBD and (No ACLF or ACLF1) | 9541 | 96.95 | 96.61 | 97.3 |
| Era 2: 2013-2018 | Donor-ACLF strata | DCD and (ACLF 2,3,>3) | 231 | 94.12 | 91.07 | 97.28 |
| Era 2: 2013-2018 | Donor-ACLF strata | DCD and (No ACLF or ACLF1) | 943 | 93.98 | 92.45 | 95.54 |
| Era 3: 2019-2023 | Donor type | DBD | 22444 | 97.48 | 97.28 | 97.69 |
| Era 3: 2019-2023 | Donor type | DCD | 2930 | 94.7 | 93.87 | 95.53 |
| Era 3: 2019-2023 | Donor-ACLF strata | DBD and (ACLF 2,3,>3) | 9512 | 97.51 | 97.19 | 97.83 |
| Era 3: 2019-2023 | Donor-ACLF strata | DBD and (No ACLF or ACLF1) | 12932 | 97.47 | 97.19 | 97.74 |
| Era 3: 2019-2023 | Donor-ACLF strata | DCD and (ACLF 2,3,>3) | 240 | 96.03 | 93.51 | 98.61 |
| Era 3: 2019-2023 | Donor-ACLF strata | DCD and (No ACLF or ACLF1) | 2690 | 94.58 | 93.71 | 95.46 |

**Table C2. Effect-size analysis for 1-year graft failure by donor type**

| **Period** | **Reference** | **Comparison** | **Reference N** | **Comparison N** | **Reference failure, %** | **Comparison failure, %** | **Absolute difference, %** | **RD 95% CI lower** | **RD 95% CI upper** | **Risk ratio** | **RR 95% CI lower** | **RR 95% CI upper** | **MCID threshold, %** | **Exceeds MCID** |
| --- | --- | --- | --- | --- | --- | --- | --- | --- | --- | --- | --- | --- | --- | --- |
| Overall: 2004-2023 | DBD | DCD | 62510 | 4691 | 4.14 | 5.8 | 1.66 | 0.97 | 2.35 | 1.4 | 1.24 | 1.58 | 9.96 | False |
| Era 1: 2004-2012 | DBD | DCD | 22693 | 587 | 6.6 | 9.54 | 2.94 | 0.54 | 5.34 | 1.45 | 1.12 | 1.86 | 12.42 | False |
| Era 2: 2013-2018 | DBD | DCD | 17373 | 1174 | 3.11 | 5.79 | 2.68 | 1.32 | 4.04 | 1.86 | 1.46 | 2.38 | 8.68 | False |
| Era 3: 2019-2023 | DBD | DCD | 22444 | 2930 | 2.45 | 5.05 | 2.6 | 1.78 | 3.42 | 2.06 | 1.73 | 2.46 | 7.73 | False |

**Table C3. Effect-size analysis for 1-year graft failure by donor-ACLF strata**

| **Period** | **Reference** | **Comparison** | **Reference N** | **Comparison N** | **Reference failure, %** | **Comparison failure, %** | **Absolute difference, %** | **RD 95% CI lower** | **RD 95% CI upper** | **Risk ratio** | **RR 95% CI lower** | **RR 95% CI upper** | **MCID threshold, %** | **Exceeds MCID** |
| --- | --- | --- | --- | --- | --- | --- | --- | --- | --- | --- | --- | --- | --- | --- |
| Overall: 2004-2023 | DBD and (No ACLF or ACLF1) | DBD and (ACLF 2,3,>3) | 37834 | 24676 | 4.19 | 4.07 | -0.12 | -0.44 | 0.2 | 0.97 | 0.9 | 1.05 | 10.01 | False |
| Overall: 2004-2023 | DBD and (No ACLF or ACLF1) | DCD and (No ACLF or ACLF1) | 37834 | 4081 | 4.19 | 5.73 | 1.55 | 0.81 | 2.29 | 1.37 | 1.2 | 1.56 | 10.01 | False |
| Overall: 2004-2023 | DBD and (No ACLF or ACLF1) | DCD and (ACLF 2,3,>3) | 37834 | 610 | 4.19 | 6.23 | 2.04 | 0.11 | 3.97 | 1.49 | 1.09 | 2.03 | 10.01 | False |
| Era 1: 2004-2012 | DBD and (No ACLF or ACLF1) | DBD and (ACLF 2,3,>3) | 15361 | 7332 | 6.37 | 7.08 | 0.71 | 0 | 1.41 | 1.11 | 1 | 1.23 | 12.21 | False |
| Era 1: 2004-2012 | DBD and (No ACLF or ACLF1) | DCD and (No ACLF or ACLF1) | 15361 | 448 | 6.37 | 8.93 | 2.56 | -0.11 | 5.22 | 1.4 | 1.04 | 1.9 | 12.21 | False |
| Era 1: 2004-2012 | DBD and (No ACLF or ACLF1) | DCD and (ACLF 2,3,>3) | 15361 | 139 | 6.37 | 11.51 | 5.14 | -0.18 | 10.46 | 1.81 | 1.14 | 2.88 | 12.21 | False |
| Era 2: 2013-2018 | DBD and (No ACLF or ACLF1) | DBD and (ACLF 2,3,>3) | 9541 | 7832 | 2.99 | 3.26 | 0.27 | -0.25 | 0.79 | 1.09 | 0.92 | 1.29 | 8.51 | False |
| Era 2: 2013-2018 | DBD and (No ACLF or ACLF1) | DCD and (No ACLF or ACLF1) | 9541 | 943 | 2.99 | 5.83 | 2.85 | 1.31 | 4.38 | 1.95 | 1.48 | 2.59 | 8.51 | False |
| Era 2: 2013-2018 | DBD and (No ACLF or ACLF1) | DCD and (ACLF 2,3,>3) | 9541 | 231 | 2.99 | 5.63 | 2.64 | -0.35 | 5.63 | 1.88 | 1.1 | 3.23 | 8.51 | False |
| Era 3: 2019-2023 | DBD and (No ACLF or ACLF1) | DBD and (ACLF 2,3,>3) | 12932 | 9512 | 2.47 | 2.42 | -0.06 | -0.47 | 0.35 | 0.98 | 0.83 | 1.16 | 7.77 | False |
| Era 3: 2019-2023 | DBD and (No ACLF or ACLF1) | DCD and (No ACLF or ACLF1) | 12932 | 2690 | 2.47 | 5.17 | 2.69 | 1.81 | 3.57 | 2.09 | 1.72 | 2.54 | 7.77 | False |
| Era 3: 2019-2023 | DBD and (No ACLF or ACLF1) | DCD and (ACLF 2,3,>3) | 12932 | 240 | 2.47 | 3.75 | 1.28 | -1.14 | 3.69 | 1.51 | 0.79 | 2.9 | 7.77 | False |

**Table C4. One-year competing-risk summary in the overall cohort**

| **Period** | **N** | **Graft failure within 1 year** | **DWFG within 1 year** | **Censored within 1 year** | **Graft failure rate, %** | **DWFG rate, %** |
| --- | --- | --- | --- | --- | --- | --- |
| Overall: 2004-2023 | 67201 | 2860 | 3854 | 60487 | 4.26 | 5.74 |
| Era 1: 2004-2012 | 23280 | 1554 | 1708 | 20018 | 6.68 | 7.34 |
| Era 2: 2013-2018 | 18547 | 608 | 1048 | 16891 | 3.28 | 5.65 |
| Era 3: 2019-2023 | 25374 | 698 | 1098 | 23578 | 2.75 | 4.33 |

**Table C5. One-year competing-risk summary by donor type**

| **Period** | **Donor type** | **N** | **Graft failure within 1 year** | **DWFG within 1 year** | **Censored within 1 year** | **Graft failure rate, %** | **DWFG rate, %** |
| --- | --- | --- | --- | --- | --- | --- | --- |
| Overall: 2004-2023 | DBD | 62510 | 2588 | 3631 | 56291 | 4.14 | 5.81 |
| Overall: 2004-2023 | DCD | 4691 | 272 | 223 | 4196 | 5.8 | 4.75 |
| Era 1: 2004-2012 | DBD | 22693 | 1498 | 1655 | 19540 | 6.6 | 7.29 |
| Era 1: 2004-2012 | DCD | 587 | 56 | 53 | 478 | 9.54 | 9.03 |
| Era 2: 2013-2018 | DBD | 17373 | 540 | 988 | 15845 | 3.11 | 5.69 |
| Era 2: 2013-2018 | DCD | 1174 | 68 | 60 | 1046 | 5.79 | 5.11 |
| Era 3: 2019-2023 | DBD | 22444 | 550 | 988 | 20906 | 2.45 | 4.4 |
| Era 3: 2019-2023 | DCD | 2930 | 148 | 110 | 2672 | 5.05 | 3.75 |

**Table C6. One-year competing-risk summary by donor-ACLF strata**

| **Period** | **Strata group** | **N** | **Graft failure within 1 year** | **DWFG within 1 year** | **Censored within 1 year** | **Graft failure rate, %** | **DWFG rate, %** |
| --- | --- | --- | --- | --- | --- | --- | --- |
| Overall: 2004-2023 | DBD and (ACLF 2,3,>3) | 24676 | 1004 | 1932 | 21740 | 4.07 | 7.83 |
| Overall: 2004-2023 | DBD and (No ACLF or ACLF1) | 37834 | 1584 | 1699 | 34551 | 4.19 | 4.49 |
| Overall: 2004-2023 | DCD and (ACLF 2,3,>3) | 610 | 38 | 60 | 512 | 6.23 | 9.84 |
| Overall: 2004-2023 | DCD and (No ACLF or ACLF1) | 4081 | 234 | 163 | 3684 | 5.73 | 3.99 |
| Era 1: 2004-2012 | DBD and (ACLF 2,3,>3) | 7332 | 519 | 821 | 5992 | 7.08 | 11.2 |
| Era 1: 2004-2012 | DBD and (No ACLF or ACLF1) | 15361 | 979 | 834 | 13548 | 6.37 | 5.43 |
| Era 1: 2004-2012 | DCD and (ACLF 2,3,>3) | 139 | 16 | 24 | 99 | 11.51 | 17.27 |
| Era 1: 2004-2012 | DCD and (No ACLF or ACLF1) | 448 | 40 | 29 | 379 | 8.93 | 6.47 |
| Era 2: 2013-2018 | DBD and (ACLF 2,3,>3) | 7832 | 255 | 585 | 6992 | 3.26 | 7.47 |
| Era 2: 2013-2018 | DBD and (No ACLF or ACLF1) | 9541 | 285 | 403 | 8853 | 2.99 | 4.22 |
| Era 2: 2013-2018 | DCD and (ACLF 2,3,>3) | 231 | 13 | 21 | 197 | 5.63 | 9.09 |
| Era 2: 2013-2018 | DCD and (No ACLF or ACLF1) | 943 | 55 | 39 | 849 | 5.83 | 4.14 |
| Era 3: 2019-2023 | DBD and (ACLF 2,3,>3) | 9512 | 230 | 526 | 8756 | 2.42 | 5.53 |
| Era 3: 2019-2023 | DBD and (No ACLF or ACLF1) | 12932 | 320 | 462 | 12150 | 2.47 | 3.57 |
| Era 3: 2019-2023 | DCD and (ACLF 2,3,>3) | 240 | 9 | 15 | 216 | 3.75 | 6.25 |
| Era 3: 2019-2023 | DCD and (No ACLF or ACLF1) | 2690 | 139 | 95 | 2456 | 5.17 | 3.53 |

**Table C7. Fine-Gray subdistribution hazard models for 1-year graft failure**

| **Period** | **Term** | **SHR (95% CI)** | **P value** |
| --- | --- | --- | --- |
| Overall: 2004-2023 | DBD and (No ACLF or ACLF1) vs DBD and (ACLF 2,3,>3) | 0.948 (0.874-1.027) | 0.191 |
| Overall: 2004-2023 | DCD and (ACLF 2,3,>3) vs DBD and (ACLF 2,3,>3) | 1.775 (1.283-2.455) | <0.001 |
| Overall: 2004-2023 | DCD and (No ACLF or ACLF1) vs DBD and (ACLF 2,3,>3) | 1.448 (1.257-1.669) | <0.001 |
| Overall: 2004-2023 | DON_HIST_DIAB | 1.308 (1.182-1.448) | <0.001 |
| Overall: 2004-2023 | DON_HTN | 1.215 (1.114-1.325) | <0.001 |
| Overall: 2004-2023 | DON_AGE | 1.010 (1.008-1.013) | <0.001 |
| Era 1: 2004-2012 | DBD and (No ACLF or ACLF1) vs DBD and (ACLF 2,3,>3) | 0.852 (0.765-0.948) | 0.003 |
| Era 1: 2004-2012 | DCD and (ACLF 2,3,>3) vs DBD and (ACLF 2,3,>3) | 1.985 (1.210-3.255) | 0.007 |
| Era 1: 2004-2012 | DCD and (No ACLF or ACLF1) vs DBD and (ACLF 2,3,>3) | 1.417 (1.027-1.957) | 0.034 |
| Era 1: 2004-2012 | DON_HIST_DIAB | 1.335 (1.157-1.541) | <0.001 |
| Era 1: 2004-2012 | DON_HTN | 1.082 (0.962-1.218) | 0.189 |
| Era 1: 2004-2012 | DON_AGE | 1.014 (1.010-1.018) | <0.001 |
| Era 2: 2013-2018 | DBD and (No ACLF or ACLF1) vs DBD and (ACLF 2,3,>3) | 0.850 (0.716-1.010) | 0.065 |
| Era 2: 2013-2018 | DCD and (ACLF 2,3,>3) vs DBD and (ACLF 2,3,>3) | 2.003 (1.139-3.522) | 0.016 |
| Era 2: 2013-2018 | DCD and (No ACLF or ACLF1) vs DBD and (ACLF 2,3,>3) | 1.904 (1.425-2.546) | <0.001 |
| Era 2: 2013-2018 | DON_HIST_DIAB | 1.461 (1.178-1.813) | <0.001 |
| Era 2: 2013-2018 | DON_HTN | 1.220 (1.008-1.475) | 0.041 |
| Era 2: 2013-2018 | DON_AGE | 1.008 (1.001-1.015) | 0.019 |
| Era 3: 2019-2023 | DBD and (No ACLF or ACLF1) vs DBD and (ACLF 2,3,>3) | 0.923 (0.778-1.095) | 0.357 |
| Era 3: 2019-2023 | DCD and (ACLF 2,3,>3) vs DBD and (ACLF 2,3,>3) | 1.750 (0.899-3.407) | 0.100 |
| Era 3: 2019-2023 | DCD and (No ACLF or ACLF1) vs DBD and (ACLF 2,3,>3) | 2.127 (1.724-2.624) | <0.001 |
| Era 3: 2019-2023 | DON_HIST_DIAB | 1.460 (1.201-1.775) | <0.001 |
| Era 3: 2019-2023 | DON_HTN | 1.487 (1.248-1.772) | <0.001 |
| Era 3: 2019-2023 | DON_AGE | 1.003 (0.997-1.009) | 0.332 |

**Supplementary Tables**

# **Table D1. Donor Characteristics by Donor Type, 2004-01-01 to 2023-12-31**

| **Characteristic** | **DBD**  N = 62,510^1^ | **DCD**  N = 4,691^1^ | **p-value**^2^ |
| --- | --- | --- | --- |
| **Calculated Donor Age in Years at Organ Recovery or Referral Date** | 41.00 (28.00, 54.00) | 38.00 (27.00, 49.00) | <0.001 |
| **Donor/s Age in Months** | 501.00 (338.00, 655.00) | 458.00 (326.00, 593.00) | <0.001 |
| **most recent serum creatinine** | 1.10 (0.80, 1.70) | 0.80 (0.60, 1.16) | <0.001 |
| **BUN** | 19.00 (12.00, 30.00) | 18.00 (12.00, 26.00) | <0.001 |
| **Cancer Free Interval:** | 6.00 (1.00, 15.00) | 5.00 (0.00, 11.00) | 0.077 |
| **Cardiac Index (final) //Cardiac Index:** | 3.80 (3.10, 4.70) | 3.90 (3.00, 4.40) | >0.9 |
| **Cardiac Index (initial) //Cardiac Index: (L/min/sq.m)** | 3.50 (2.70, 4.50) | 4.30 (4.00, 4.90) | 0.079 |
| **CO (final) (L/min) //CO:** | 7.30 (5.90, 9.00) | 8.40 (6.20, 9.10) | 0.5 |
| **CO (initial) (L/min) //CO: (L/min)** | 6.70 (5.10, 8.50) | 8.40 (7.50, 10.50) | 0.038 |
| **Serum Creatinine** | 1.10 (0.80, 1.70) | 0.80 (0.60, 1.17) | <0.001 |
| **CVP (final) //CVP:** | 9.00 (7.00, 12.00) | 10.00 (7.50, 15.50) | 0.071 |
| **CVP (initial) //CVP: (mm/Hg)** | 9.00 (6.00, 12.00) | 10.00 (6.00, 13.00) | 0.5 |
| **LV Ejection Fraction %** | 60.00 (55.00, 65.00) | 60.00 (55.00, 65.00) | 0.5 |
| **Final Serum Creatinine** | 1.10 (0.80, 1.75) | 0.80 (0.60, 1.16) | <0.001 |
| **Hematocrit:** | 29.20 (25.90, 33.10) | 30.00 (25.70, 34.60) | <0.001 |
| **Donor/s Height in centimeters** | 172.72 (165.00, 180.00) | 173.00 (165.00, 180.00) | <0.001 |
| **INR:** | 1.24 (1.10, 1.40) | 1.20 (1.10, 1.30) | <0.001 |
| **Glomeruli Observed on Kidney biopsy** | 48.00 (27.50, 68.50) | 45.50 (30.50, 62.50) | >0.9 |
| **Percent Glomerular Sclerosis on Kidney biopsy** | 9.00 (2.00, 21.00) | 2.50 (0.00, 8.00) | 0.004 |
| **% Glomerulosclerosis (Left Kidney)** | 1.00 (1.00, 2.00) | 1.00 (1.00, 2.00) | <0.001 |
| **Final Resistance Prior to Shipping (left) //Final Resistance Prior to Shipping:** | 0.21 (0.16, 0.29) | 0.20 (0.15, 0.27) | <0.001 |
| **Serum Lipase** | 27.00 (14.00, 69.00) | 36.00 (18.00, 85.00) | <0.001 |
| **% Macro vesicular fat:** | 5.00 (0.00, 10.00) | 5.00 (0.00, 10.00) | <0.001 |
| **% Micro vesicular fat:** | 5.00 (0.00, 10.00) | 0.00 (0.00, 5.00) | <0.001 |
| **MAP (final) //Map:** | 86.00 (76.00, 98.00) | 86.00 (76.00, 99.00) | 0.8 |
| **MAP (initial) //Map: (mm/Hg)** | 84.00 (73.00, 96.00) | 84.00 (74.00, 97.00) | 0.7 |
| **Maximum Donor Creatinine** | 1.10 (0.80, 1.70) | 0.80 (0.60, 1.18) | <0.001 |
| **PA Diastolic (final) //PA Diastolic:** | 17.00 (13.00, 21.00) | 19.50 (16.00, 24.00) | 0.070 |
| **PA Diastolic (initial) //PA Diastolic: (mm/Hg)** | 17.00 (13.00, 21.00) | 17.50 (13.00, 22.00) | 0.6 |
| **PA Systolic (final) //PA Systolic:** | 28.00 (23.50, 35.00) | 30.50 (28.50, 41.00) | 0.045 |
| **PA Systolic (initial) //PA Systolic: (mm/Hg)** | 29.00 (24.00, 35.00) | 33.00 (29.00, 38.00) | 0.2 |
| **pCO2:** | 37.90 (33.80, 42.00) | 38.60 (34.00, 44.00) | <0.001 |
| **PCWP (final) //PCWP:** | 12.00 (9.00, 15.00) | 10.00 (10.00, 13.00) | 0.6 |
| **PCWP (initial) //PCWP: (mm/Hg)** | 12.00 (9.00, 16.00) | 14.00 (11.00, 17.00) | 0.3 |
| **Peak Serum Creatinine** | 1.50 (1.10, 2.25) | 1.29 (1.00, 1.73) | <0.001 |
| **Blood PH:** | 7.41 (7.36, 7.45) | 7.42 (7.37, 7.46) | <0.001 |
| **Lung pO2 on 100%** | 185.00 (113.00, 391.00) | 131.00 (90.40, 260.00) | <0.001 |
| **Lung pO2 on Fio2 //If Yes, Lung pO2 on FiO2 of:** | 100.00 (50.00, 100.00) | 65.00 (40.00, 100.00) | <0.001 |
| **% Glomerulosclerosis (Right Kidney)** | 1.00 (1.00, 2.00) | 1.00 (1.00, 2.00) | <0.001 |
| **Final Resistance Prior to Shipping (right) //Final Resistance Prior to Shipping:** | 0.20 (0.15, 0.27) | 0.19 (0.14, 0.26) | <0.001 |
| **Serum Amylase** | 61.00 (33.00, 121.00) | 56.00 (34.00, 104.00) | 0.036 |
| **SGOT/AST** | 41.00 (25.00, 80.00) | 51.00 (33.00, 82.00) | <0.001 |
| **SGPT/ALT** | 35.00 (21.00, 70.00) | 40.00 (24.00, 74.00) | <0.001 |
| **Serum Sodium //Last Serum Sodium Prior to Procurement:** | 148.00 (142.00, 153.00) | 146.00 (141.00, 152.00) | <0.001 |
| **SVR (final) //SVR:** | 864.00 (687.00, 1,136.00) | 824.50 (763.00, 1,017.00) | >0.9 |
| **SVR (initial) //SVR: (dynes/sec/cm)^5)** | 993.00 (734.00, 1,321.00) | 826.00 (611.00, 848.00) | 0.032 |
| **Total Bilirubin** | 0.70 (0.40, 1.00) | 0.50 (0.40, 0.80) | <0.001 |
| **Troponin I** | 0.21 (0.05, 1.37) | 0.26 (0.04, 6.79) | <0.001 |
| **Troponin T** | 0.18 (0.02, 15.00) | 7.00 (0.06, 43.00) | <0.001 |
| **If Abnormal, # of Vessels with > 50% Stenosis** | 0.00 (0.00, 2.00) | 1.00 (0.00, 1.00) | 0.051 |
| **Estimated Warm Ischemic Time in minutes** | 0.00 (0.00, 0.00) | 19.00 (14.00, 24.00) | <0.001 |
| **Donor/s Weight in kilograms** | 79.40 (68.00, 93.00) | 80.00 (68.00, 94.00) | 0.10 |
| **Donor Risk Index (DRI)** | 1.65 (1.45, 1.95) | 2.35 (2.09, 2.73) | <0.001 |
| **Donor/s Blood Type** |  |  | <0.001 |
| A | 9,536 (15%) | 766 (16%) |  |
| A1 | 11,502 (18%) | 929 (20%) |  |
| A1B | 764 (1.2%) | 23 (0.5%) |  |
| A2 | 2,133 (3.4%) | 197 (4.2%) |  |
| A2B | 307 (0.5%) | 16 (0.3%) |  |
| AB | 1,003 (1.6%) | 24 (0.5%) |  |
| B | 7,075 (11%) | 427 (9.1%) |  |
| O | 30,190 (48%) | 2,309 (49%) |  |
| **CVA cause of death** |  |  | <0.001 |
| No | 40,643 (65%) | 3,882 (83%) |  |
| Yes | 20,118 (32%) | 809 (17%) |  |
| Missing | 1,749 (2.8%) | 0 (0%) |  |
| **history of hypertension** |  |  | <0.001 |
| No | 38,617 (62%) | 3,419 (73%) |  |
| Yes | 21,216 (34%) | 1,233 (26%) |  |
| Missing | 2,677 (4.3%) | 39 (0.8%) |  |
| **Allocate Remaining Liver Segment?** |  |  | 0.008 |
| Missing | 62,270 (100%) | 4,686 (100%) |  |
| No | 118 (0.2%) | 4 (<0.1%) |  |
| Yes | 122 (0.2%) | 1 (<0.1%) |  |
| **Anti-CMV** |  |  | <0.001 |
| Missing | 1 (<0.1%) | 0 (0%) |  |
| Indeterminate | 168 (0.3%) | 18 (0.4%) |  |
| Negative | 21,759 (35%) | 2,058 (44%) |  |
| Not Done | 135 (0.2%) | 15 (0.3%) |  |
| Positive | 40,415 (65%) | 2,600 (55%) |  |
| Unknown | 32 (<0.1%) | 0 (0%) |  |
| **Anti-HBC** |  |  | <0.001 |
| Indeterminate | 5 (<0.1%) | 2 (<0.1%) |  |
| Negative | 59,300 (95%) | 4,542 (97%) |  |
| Not Done | 54 (<0.1%) | 1 (<0.1%) |  |
| Positive | 3,147 (5.0%) | 146 (3.1%) |  |
| Unknown | 4 (<0.1%) | 0 (0%) |  |
| **Anti-HCV** |  |  | 0.5 |
| Missing | 0 (0%) | 0 (0%) |  |
| Cannot Disclose | 1 (<0.1%) | 0 (0%) |  |
| Indeterminate | 10 (<0.1%) | 1 (<0.1%) |  |
| Negative | 58,483 (94%) | 4,399 (94%) |  |
| Not Done | 33 (<0.1%) | 0 (0%) |  |
| Positive | 3,978 (6.4%) | 291 (6.2%) |  |
| Unknown | 5 (<0.1%) | 0 (0%) |  |
| **Anti-HIV I/II:** |  |  | <0.001 |
| Missing | 1 (<0.1%) | 0 (0%) |  |
| Negative | 58,841 (94%) | 4,164 (89%) |  |
| Not Done | 3,613 (5.8%) | 527 (11%) |  |
| Positive | 46 (<0.1%) | 0 (0%) |  |
| Unknown | 9 (<0.1%) | 0 (0%) |  |
| **Anti-HTLV I/II:** |  |  | <0.001 |
| Missing | 1 (<0.1%) | 0 (0%) |  |
| Cannot Disclose | 2 (<0.1%) | 0 (0%) |  |
| Indeterminate | 4 (<0.1%) | 0 (0%) |  |
| Negative | 24,099 (39%) | 1,159 (25%) |  |
| Not Done | 38,314 (61%) | 3,528 (75%) |  |
| Positive | 74 (0.1%) | 3 (<0.1%) |  |
| Unknown | 16 (<0.1%) | 1 (<0.1%) |  |
| **Arginine Vasopressin:** |  |  | <0.001 |
| Missing | 1,019 (1.6%) | 0 (0%) |  |
| Negative / No | 23,117 (37%) | 3,588 (76%) |  |
| Positive / Yes | 38,298 (61%) | 1,096 (23%) |  |
| Unknown | 76 (0.1%) | 7 (0.1%) |  |
| **Donor/s Cause of Death** |  |  | <0.001 |
| Anoxia | 20,552 (33%) | 2,361 (50%) |  |
| Cerebrovascular / Stroke | 20,850 (33%) | 808 (17%) |  |
| CNS Tumor | 266 (0.4%) | 4 (<0.1%) |  |
| Head Trauma | 19,635 (31%) | 1,324 (28%) |  |
| Other | 1,206 (1.9%) | 194 (4.1%) |  |
| Missing | 1 (<0.1%) | 0 (0%) |  |
| **Cardiac Arr. Since Event that led to Decl of Death** |  |  | <0.001 |
| Missing | 165 (0.3%) | 3,933 (84%) |  |
| No | 58,212 (93%) | 717 (15%) |  |
| Yes | 4,133 (6.6%) | 41 (0.9%) |  |
| **Donor RNA Nat serology value for Chagas test** |  |  | <0.001 |
| Missing | 27,811 (44%) | 863 (18%) |  |
| Cannot Disclose | 1 (<0.1%) | 0 (0%) |  |
| Indeterminate | 3 (<0.1%) | 0 (0%) |  |
| Negative | 123 (0.2%) | 6 (0.1%) |  |
| Not Done | 34,564 (55%) | 3,822 (81%) |  |
| Positive | 1 (<0.1%) | 0 (0%) |  |
| Unknown | 7 (<0.1%) | 0 (0%) |  |
| **Chest X-ray:** |  |  | <0.001 |
| No chest x-ray | 377 (0.6%) | 17 (0.4%) |  |
| Normal | 12,826 (21%) | 725 (15%) |  |
| Abnormal-left | 5,673 (9.1%) | 390 (8.3%) |  |
| Abnormal-right | 5,788 (9.3%) | 418 (8.9%) |  |
| Abnormal-both | 30,378 (49%) | 2,828 (60%) |  |
| Results Unknown | 188 (0.3%) | 10 (0.2%) |  |
| Unknown if chest x-ray performed | 230 (0.4%) | 0 (0%) |  |
| Missing | 7,050 (11%) | 303 (6.5%) |  |
| **Donor/s Citizenship** |  |  | <0.001 |
| US Citizen | 58,176 (93%) | 4,400 (94%) |  |
| Resident Alien | 284 (0.5%) | 5 (0.1%) |  |
| Non-Resident Alien, Specify Country | 114 (0.2%) | 0 (0%) |  |
| Non-US Citizen/US Resident | 1,205 (1.9%) | 52 (1.1%) |  |
| Non-US Citizen/Non-US Resident | 175 (0.3%) | 13 (0.3%) |  |
| Unknown | 2,555 (4.1%) | 221 (4.7%) |  |
| Missing | 1 (<0.1%) | 0 (0%) |  |
| **Clinical Infection** |  |  | <0.001 |
| Missing | 4 (<0.1%) | 0 (0%) |  |
| Negative / No | 21,481 (34%) | 1,363 (29%) |  |
| Positive / Yes | 40,350 (65%) | 3,299 (70%) |  |
| Unknown | 675 (1.1%) | 29 (0.6%) |  |
| **Donor cause of death is stroke** |  |  | <0.001 |
| No | 41,243 (66%) | 3,865 (82%) |  |
| Yes | 21,267 (34%) | 826 (18%) |  |
| **Alcohol Dependency Last 6 Months** |  |  | 0.002 |
| Missing | 62,281 (100%) | 4,691 (100%) |  |
| Negative / No | 60 (<0.1%) | 0 (0%) |  |
| Positive / Yes | 165 (0.3%) | 0 (0%) |  |
| Unknown | 4 (<0.1%) | 0 (0%) |  |
| **Donor/s Continued Use in Last 6 Months** |  |  | <0.001 |
| Missing | 47,866 (77%) | 3,767 (80%) |  |
| Negative / No | 2,440 (3.9%) | 120 (2.6%) |  |
| Positive / Yes | 12,118 (19%) | 793 (17%) |  |
| Unknown | 86 (0.1%) | 11 (0.2%) |  |
| **Cocaine Use Last 6 Months** |  |  | 0.005 |
| Missing | 51,037 (82%) | 3,756 (80%) |  |
| Negative / No | 3,908 (6.3%) | 333 (7.1%) |  |
| Positive / Yes | 6,072 (9.7%) | 459 (9.8%) |  |
| Unknown | 1,493 (2.4%) | 143 (3.0%) |  |
| **IV Drug Last 6 Months** |  |  | 0.4 |
| Missing | 62,474 (100%) | 4,691 (100%) |  |
| Negative / No | 14 (<0.1%) | 0 (0%) |  |
| Positive / Yes | 14 (<0.1%) | 0 (0%) |  |
| Unknown | 8 (<0.1%) | 0 (0%) |  |
| **Other Drug Abuse Last 6 Months** |  |  | <0.001 |
| Missing | 36,544 (58%) | 2,485 (53%) |  |
| Negative / No | 4,891 (7.8%) | 346 (7.4%) |  |
| Positive / Yes | 18,776 (30%) | 1,667 (36%) |  |
| Unknown | 2,299 (3.7%) | 193 (4.1%) |  |
| **Coronary Angiogram** |  |  | <0.001 |
| No | 47,958 (77%) | 4,520 (96%) |  |
| Yes, normal | 10,435 (17%) | 112 (2.4%) |  |
| Yes, not normal | 3,374 (5.4%) | 47 (1.0%) |  |
| 4 | 104 (0.2%) | 7 (0.1%) |  |
| 5 | 26 (<0.1%) | 5 (0.1%) |  |
| Missing | 613 (1.0%) | 0 (0%) |  |
| **If not DCD, Was this a consented DCD donor that progressed to brain death?** |  |  | <0.001 |
| Missing | 10,506 (17%) | 4,691 (100%) |  |
| No | 47,352 (76%) | 0 (0%) |  |
| Yes | 4,652 (7.4%) | 0 (0%) |  |
| **Meds given Donor: DDAVP** |  |  | <0.001 |
| Missing | 4 (<0.1%) | 0 (0%) |  |
| Negative / No | 55,341 (89%) | 4,352 (93%) |  |
| Positive / Yes | 7,109 (11%) | 334 (7.1%) |  |
| Unknown | 56 (<0.1%) | 5 (0.1%) |  |
| **Cirumstances of Death** |  |  | <0.001 |
| MVA | 9,597 (15%) | 805 (17%) |  |
| SUICIDE | 5,959 (9.5%) | 685 (15%) |  |
| HOMICIDE | 3,480 (5.6%) | 147 (3.1%) |  |
| CHILD-ABUSE | 9 (<0.1%) | 0 (0%) |  |
| NON-MVA | 8,386 (13%) | 756 (16%) |  |
| DEATH FROM NATURAL CAUSES | 25,171 (40%) | 1,764 (38%) |  |
| NONE OF THE ABOVE | 9,907 (16%) | 534 (11%) |  |
| Missing | 1 (<0.1%) | 0 (0%) |  |
| **Mechanism of Death** |  |  | <0.001 |
| DROWNING | 295 (0.5%) | 34 (0.7%) |  |
| SEIZURE | 529 (0.8%) | 62 (1.3%) |  |
| DRUG INTOXICATION | 7,273 (12%) | 657 (14%) |  |
| ASPHYXIATION | 2,370 (3.8%) | 485 (10%) |  |
| CARDIOVASCULAR | 8,900 (14%) | 946 (20%) |  |
| ELECTRICAL | 39 (<0.1%) | 4 (<0.1%) |  |
| GUNSHOT WOUND | 6,420 (10%) | 361 (7.7%) |  |
| STAB | 124 (0.2%) | 11 (0.2%) |  |
| BLUNT INJURY | 12,619 (20%) | 980 (21%) |  |
| INTRACRANIAL HEMORRHAGE/STROKE | 21,248 (34%) | 838 (18%) |  |
| DEATH FROM NATURAL CAUSES | 1,501 (2.4%) | 207 (4.4%) |  |
| NONE OF THE ABOVE | 1,191 (1.9%) | 106 (2.3%) |  |
| Missing | 1 (<0.1%) | 0 (0%) |  |
| **EBNA (Epstein-Barr nuclear antigen)** |  |  | <0.001 |
| Missing | 5,651 (9.0%) | 0 (0%) |  |
| Cannot Disclose | 23 (<0.1%) | 1 (<0.1%) |  |
| Indeterminate | 77 (0.1%) | 9 (0.2%) |  |
| Negative | 1,232 (2.0%) | 153 (3.3%) |  |
| Not Done | 46,300 (74%) | 3,747 (80%) |  |
| Positive | 9,065 (15%) | 778 (17%) |  |
| Unknown | 162 (0.3%) | 3 (<0.1%) |  |
| **EBV//IgG:** |  |  | <0.001 |
| Missing | 5,568 (8.9%) | 0 (0%) |  |
| Cannot Disclose | 4 (<0.1%) | 0 (0%) |  |
| Indeterminate | 320 (0.5%) | 24 (0.5%) |  |
| Negative | 2,860 (4.6%) | 359 (7.7%) |  |
| Not Done | 3,838 (6.1%) | 219 (4.7%) |  |
| Positive | 49,745 (80%) | 4,088 (87%) |  |
| Unknown | 175 (0.3%) | 1 (<0.1%) |  |
| **EBV//IgM:** |  |  | <0.001 |
| Missing | 5,568 (8.9%) | 0 (0%) |  |
| Cannot Disclose | 9 (<0.1%) | 0 (0%) |  |
| Indeterminate | 140 (0.2%) | 13 (0.3%) |  |
| Negative | 45,225 (72%) | 3,613 (77%) |  |
| Not Done | 10,461 (17%) | 991 (21%) |  |
| Positive | 942 (1.5%) | 73 (1.6%) |  |
| Unknown | 165 (0.3%) | 1 (<0.1%) |  |
| **Method** |  |  | 0.017 |
| Echo | 45,250 (72%) | 2,076 (44%) |  |
| MUGA | 5 (<0.1%) | 0 (0%) |  |
| Angiogram | 771 (1.2%) | 14 (0.3%) |  |
| Missing | 16,484 (26%) | 2,601 (55%) |  |
| **SRTR Donor Ethnicity** |  |  | <0.001 |
| Latino | 9,188 (15%) | 442 (9.4%) |  |
| Non-Latino or unknown | 53,322 (85%) | 4,249 (91%) |  |
| **Does donor meet criteria to be an Expanded Donor?** |  |  | <0.001 |
| No | 47,598 (76%) | 4,197 (89%) |  |
| Yes | 14,828 (24%) | 490 (10%) |  |
| Missing | 84 (0.1%) | 4 (<0.1%) |  |
| **Meets expanded donor criteria for kidney (1= yes , 0= no)** |  |  | <0.001 |
| No | 47,079 (75%) | 4,192 (89%) |  |
| Yes | 15,431 (25%) | 499 (11%) |  |
| **Did the patient express to family or others the intent to be a donor** |  |  | <0.001 |
| Missing | 1,828 (2.9%) | 461 (9.8%) |  |
| Negative / No | 18,168 (29%) | 1,207 (26%) |  |
| Positive / Yes | 17,661 (28%) | 1,628 (35%) |  |
| Unknown | 24,853 (40%) | 1,395 (30%) |  |
| **Donor/s Gender** |  |  | <0.001 |
| Female | 24,721 (40%) | 1,527 (33%) |  |
| Male | 37,789 (60%) | 3,164 (67%) |  |
| **Hepatitis B Core Antibody Status** |  |  | <0.001 |
| Indeterminate | 10 (<0.1%) | 1 (<0.1%) |  |
| Negative | 54,723 (88%) | 4,514 (96%) |  |
| Not Done | 3,980 (6.4%) | 6 (0.1%) |  |
| Pending | 422 (0.7%) | 23 (0.5%) |  |
| Positive | 3,080 (4.9%) | 146 (3.1%) |  |
| Unknown | 255 (0.4%) | 1 (<0.1%) |  |
| Missing | 40 (<0.1%) | 0 (0%) |  |
| **Donor RNA Nat serology value for hbv test** |  |  | <0.001 |
| Missing | 27,811 (44%) | 863 (18%) |  |
| Indeterminate | 2 (<0.1%) | 0 (0%) |  |
| Negative | 34,433 (55%) | 3,809 (81%) |  |
| Not Done | 124 (0.2%) | 16 (0.3%) |  |
| Positive | 138 (0.2%) | 3 (<0.1%) |  |
| Unknown | 2 (<0.1%) | 0 (0%) |  |
| **HBsAb (Hepatitis B Surface Antibody)** |  |  | <0.001 |
| Missing | 5,567 (8.9%) | 0 (0%) |  |
| Cannot Disclose | 7 (<0.1%) | 0 (0%) |  |
| Indeterminate | 88 (0.1%) | 8 (0.2%) |  |
| Negative | 6,894 (11%) | 367 (7.8%) |  |
| Not Done | 46,691 (75%) | 4,126 (88%) |  |
| Positive | 3,216 (5.1%) | 189 (4.0%) |  |
| Unknown | 47 (<0.1%) | 1 (<0.1%) |  |
| **HBV//Surface Antigen:** |  |  | 0.5 |
| Missing | 1 (<0.1%) | 0 (0%) |  |
| Negative | 62,355 (100%) | 4,684 (100%) |  |
| Not Done | 54 (<0.1%) | 4 (<0.1%) |  |
| Positive | 88 (0.1%) | 3 (<0.1%) |  |
| Unknown | 12 (<0.1%) | 0 (0%) |  |
| **Donor RNA Nat serology value for hcv test** |  |  | <0.001 |
| Missing | 27,811 (44%) | 863 (18%) |  |
| Indeterminate | 3 (<0.1%) | 0 (0%) |  |
| Negative | 32,855 (53%) | 3,679 (78%) |  |
| Not Done | 22 (<0.1%) | 2 (<0.1%) |  |
| Positive | 1,817 (2.9%) | 147 (3.1%) |  |
| Unknown | 2 (<0.1%) | 0 (0%) |  |
| **HCV Antibody Status** |  |  | <0.001 |
| Indeterminate | 14 (<0.1%) | 1 (<0.1%) |  |
| Negative | 53,938 (86%) | 4,370 (93%) |  |
| Not Done | 3,900 (6.2%) | 4 (<0.1%) |  |
| Pending | 399 (0.6%) | 24 (0.5%) |  |
| Positive | 3,986 (6.4%) | 291 (6.2%) |  |
| Unknown | 235 (0.4%) | 1 (<0.1%) |  |
| Missing | 38 (<0.1%) | 0 (0%) |  |
| **Heavy Alcohol Use (heavy= 2+ drinks/day):** |  |  | <0.001 |
| Missing | 1,024 (1.6%) | 0 (0%) |  |
| Negative / No | 50,133 (80%) | 3,695 (79%) |  |
| Positive / Yes | 9,781 (16%) | 868 (19%) |  |
| Unknown | 1,572 (2.5%) | 128 (2.7%) |  |
| **Heparin** |  |  | 0.033 |
| Missing | 2 (<0.1%) | 0 (0%) |  |
| Negative / No | 2,058 (3.3%) | 118 (2.5%) |  |
| Positive / Yes | 60,428 (97%) | 4,570 (97%) |  |
| Unknown | 22 (<0.1%) | 3 (<0.1%) |  |
| **Donor serum creatinine > 1.5 (1= yes, 0= no)** |  |  | <0.001 |
| No | 43,780 (70%) | 3,953 (84%) |  |
| Yes | 18,727 (30%) | 738 (16%) |  |
| Missing | 3 (<0.1%) | 0 (0%) |  |
| **Alcohol Dependency** |  |  | <0.001 |
| Missing | 61,318 (98%) | 4,691 (100%) |  |
| Negative / No | 955 (1.5%) | 0 (0%) |  |
| Positive / Yes | 229 (0.4%) | 0 (0%) |  |
| Unknown | 8 (<0.1%) | 0 (0%) |  |
| **History of Cancer** |  |  | 0.086 |
| NO | 59,791 (96%) | 4,547 (97%) |  |
| CNS TUMOR - OTHER | 75 (0.1%) | 8 (0.2%) |  |
| GENITOURINARY - BLADDER | 30 (<0.1%) | 2 (<0.1%) |  |
| GENITOURINARY - UTERINE CERVIX | 204 (0.3%) | 8 (0.2%) |  |
| GENITOURINARY - UTERINE BODY ENDOMETRIAL | 23 (<0.1%) | 0 (0%) |  |
| GENITOURINARY - UTERINE BODY CHORIOCARCINOMA | 1 (<0.1%) | 0 (0%) |  |
| GENITOURINARY - VULVA | 2 (<0.1%) | 0 (0%) |  |
| GENITOURINARY - OVARIAN | 65 (0.1%) | 3 (<0.1%) |  |
| GENITOURINARY - PENIS, TESTICULAR | 32 (<0.1%) | 3 (<0.1%) |  |
| SKIN - SQUAMOUS, BASAL CELL | 659 (1.1%) | 39 (0.8%) |  |
| GENITOURINARY - PROSTATE | 122 (0.2%) | 5 (0.1%) |  |
| GENITOURINARY - KIDNEY | 37 (<0.1%) | 3 (<0.1%) |  |
| GENITOURINARY - UNKNOWN | 10 (<0.1%) | 1 (<0.1%) |  |
| GASTROINTESTINAL - ESOPHAGEAL | 2 (<0.1%) | 0 (0%) |  |
| GASTROINTESTINAL - STOMACH | 8 (<0.1%) | 0 (0%) |  |
| GASTROINTESTINAL - SMALL INTESTINE | 4 (<0.1%) | 0 (0%) |  |
| GASTROINTESTINAL - COLO-RECTAL | 34 (<0.1%) | 1 (<0.1%) |  |
| GASTROINTESTINAL - PANCREAS | 3 (<0.1%) | 1 (<0.1%) |  |
| BREAST | 166 (0.3%) | 8 (0.2%) |  |
| SKIN - MELANOMA | 64 (0.1%) | 2 (<0.1%) |  |
| THYROID | 60 (<0.1%) | 5 (0.1%) |  |
| TONGUE/THROAT | 22 (<0.1%) | 2 (<0.1%) |  |
| LARYNX | 4 (<0.1%) | 1 (<0.1%) |  |
| LUNG (include bronchial) | 11 (<0.1%) | 0 (0%) |  |
| LEUKEMIA/LYMPHOMA | 36 (<0.1%) | 6 (0.1%) |  |
| CNS TUMOR - ASTROCYTOMA | 33 (<0.1%) | 2 (<0.1%) |  |
| CNS TUMOR - GLIOBLASTOMA MULTIFORME | 41 (<0.1%) | 0 (0%) |  |
| CNS TUMOR - MEDULLOBLASTOMA | 5 (<0.1%) | 2 (<0.1%) |  |
| CNS TUMOR - NEUROBLASTOMA | 1 (<0.1%) | 0 (0%) |  |
| CNS TUMOR - MENINGIOMA | 35 (<0.1%) | 0 (0%) |  |
| UNKNOWN | 579 (0.9%) | 26 (0.6%) |  |
| OTHER, SPECIFY | 349 (0.6%) | 16 (0.3%) |  |
| Missing | 2 (<0.1%) | 0 (0%) |  |
| **Cigarette Use > 20 Pack Years - Ever** |  |  | <0.001 |
| Missing | 1 (<0.1%) | 0 (0%) |  |
| Negative / No | 46,493 (74%) | 3,691 (79%) |  |
| Positive / Yes | 14,644 (23%) | 924 (20%) |  |
| Unknown | 1,372 (2.2%) | 76 (1.6%) |  |
| **Cocaine Use** |  |  | <0.001 |
| Missing | 1,817 (2.9%) | 460 (9.8%) |  |
| Negative / No | 47,978 (77%) | 3,218 (69%) |  |
| Positive / Yes | 11,473 (18%) | 935 (20%) |  |
| Unknown | 1,242 (2.0%) | 78 (1.7%) |  |
| **History of Diabetes** |  |  | <0.001 |
| No | 54,629 (87%) | 4,254 (91%) |  |
| Unknown | 604 (1.0%) | 35 (0.7%) |  |
| Yes, >10 years | 2,737 (4.4%) | 141 (3.0%) |  |
| Yes, 0-5 years | 2,372 (3.8%) | 154 (3.3%) |  |
| Yes, 6-10 years | 1,343 (2.1%) | 65 (1.4%) |  |
| Yes, Duration Unknown | 824 (1.3%) | 42 (0.9%) |  |
| Missing | 1 (<0.1%) | 0 (0%) |  |
| **History of Hypertension** |  |  | <0.001 |
| No | 40,196 (64%) | 3,415 (73%) |  |
| Unknown | 651 (1.0%) | 38 (0.8%) |  |
| Yes, >10 years | 5,691 (9.1%) | 263 (5.6%) |  |
| Yes, 0-5 years | 8,984 (14%) | 590 (13%) |  |
| Yes, 6-10 years | 3,988 (6.4%) | 211 (4.5%) |  |
| Yes, Duration Unknown | 2,999 (4.8%) | 174 (3.7%) |  |
| Missing | 1 (<0.1%) | 0 (0%) |  |
| **Insulin Dependent** |  |  | 0.9 |
| No | 3,438 (5.5%) | 187 (4.0%) |  |
| Unknown | 622 (1.0%) | 40 (0.9%) |  |
| Yes, >10 years | 1,348 (2.2%) | 75 (1.6%) |  |
| Yes, 0-5 years | 853 (1.4%) | 49 (1.0%) |  |
| Yes, 6-10 years | 457 (0.7%) | 26 (0.6%) |  |
| Yes, Duration Unknown | 557 (0.9%) | 25 (0.5%) |  |
| Missing | 55,235 (88%) | 4,289 (91%) |  |
| **IV Drug Use - Pre-6/30/2004** |  |  | <0.001 |
| Missing | 61,319 (98%) | 4,691 (100%) |  |
| Negative / No | 1,144 (1.8%) | 0 (0%) |  |
| Positive / Yes | 36 (<0.1%) | 0 (0%) |  |
| Unknown | 11 (<0.1%) | 0 (0%) |  |
| **Other Drug Abuse** |  |  | <0.001 |
| Missing | 1,816 (2.9%) | 460 (9.8%) |  |
| Negative / No | 33,849 (54%) | 1,984 (42%) |  |
| Positive / Yes | 25,967 (42%) | 2,206 (47%) |  |
| Unknown | 878 (1.4%) | 41 (0.9%) |  |
| **History of Previous MI** |  |  | <0.001 |
| Missing | 613 (1.0%) | 0 (0%) |  |
| Negative / No | 58,247 (93%) | 4,459 (95%) |  |
| Positive / Yes | 2,663 (4.3%) | 180 (3.8%) |  |
| Unknown | 987 (1.6%) | 52 (1.1%) |  |
| **Donor RNA Nat serology value for hiv test** |  |  | <0.001 |
| Missing | 27,811 (44%) | 863 (18%) |  |
| Indeterminate | 2 (<0.1%) | 0 (0%) |  |
| Negative | 34,596 (55%) | 3,812 (81%) |  |
| Not Done | 68 (0.1%) | 15 (0.3%) |  |
| Positive | 31 (<0.1%) | 1 (<0.1%) |  |
| Unknown | 2 (<0.1%) | 0 (0%) |  |
| **Donor/s Home State** |  |  | <0.001 |
| ALASKA | 139 (0.2%) | 3 (<0.1%) |  |
| ALABAMA | 1,317 (2.1%) | 55 (1.2%) |  |
| ARKANSAS | 710 (1.1%) | 64 (1.4%) |  |
| PAGO PAGO | 4 (<0.1%) | 0 (0%) |  |
| ARIZONA | 1,157 (1.9%) | 152 (3.2%) |  |
| CALIFORNIA | 5,536 (8.9%) | 347 (7.4%) |  |
| COLORADO | 716 (1.1%) | 114 (2.4%) |  |
| CONNECTICUT | 527 (0.8%) | 20 (0.4%) |  |
| DISTRICT OF COLUMBIA | 138 (0.2%) | 7 (0.1%) |  |
| DELAWARE | 323 (0.5%) | 10 (0.2%) |  |
| FLORIDA | 4,677 (7.5%) | 431 (9.2%) |  |
| GEORGIA | 2,375 (3.8%) | 123 (2.6%) |  |
| GUAM | 1 (<0.1%) | 0 (0%) |  |
| HAWAII | 201 (0.3%) | 2 (<0.1%) |  |
| IOWA | 515 (0.8%) | 54 (1.2%) |  |
| IDAHO | 249 (0.4%) | 41 (0.9%) |  |
| ILLINOIS | 2,384 (3.8%) | 198 (4.2%) |  |
| INDIANA | 1,668 (2.7%) | 167 (3.6%) |  |
| KANSAS | 747 (1.2%) | 55 (1.2%) |  |
| KENTUCKY | 950 (1.5%) | 80 (1.7%) |  |
| LOUISIANA | 1,461 (2.3%) | 102 (2.2%) |  |
| MASSACHUSETTS | 816 (1.3%) | 55 (1.2%) |  |
| MARYLAND | 1,272 (2.0%) | 81 (1.7%) |  |
| MAINE | 201 (0.3%) | 12 (0.3%) |  |
| MICHIGAN | 1,886 (3.0%) | 183 (3.9%) |  |
| MINNESOTA | 739 (1.2%) | 79 (1.7%) |  |
| MISSOURI | 1,778 (2.8%) | 98 (2.1%) |  |
| MISSISSIPPI | 821 (1.3%) | 44 (0.9%) |  |
| MONTANA | 152 (0.2%) | 15 (0.3%) |  |
| FOREIGN COUNTRY | 34 (<0.1%) | 2 (<0.1%) |  |
| NORTH CAROLINA | 1,933 (3.1%) | 155 (3.3%) |  |
| NORTH DAKOTA | 106 (0.2%) | 13 (0.3%) |  |
| NEBRASKA | 338 (0.5%) | 48 (1.0%) |  |
| NEW HAMPSHIRE | 174 (0.3%) | 8 (0.2%) |  |
| NEW JERSEY | 1,492 (2.4%) | 61 (1.3%) |  |
| NEW MEXICO | 406 (0.6%) | 33 (0.7%) |  |
| NEVADA | 692 (1.1%) | 68 (1.4%) |  |
| NEW YORK | 2,560 (4.1%) | 132 (2.8%) |  |
| OHIO | 2,452 (3.9%) | 278 (5.9%) |  |
| OKLAHOMA | 886 (1.4%) | 88 (1.9%) |  |
| OREGON | 467 (0.7%) | 51 (1.1%) |  |
| PENNSYLVANIA | 3,013 (4.8%) | 165 (3.5%) |  |
| PUERTO RICO | 908 (1.5%) | 1 (<0.1%) |  |
| RHODE ISLAND | 105 (0.2%) | 8 (0.2%) |  |
| SOUTH CAROLINA | 1,311 (2.1%) | 55 (1.2%) |  |
| SOUTH DAKOTA | 111 (0.2%) | 10 (0.2%) |  |
| TENNESSEE | 1,885 (3.0%) | 110 (2.3%) |  |
| TEXAS | 5,160 (8.3%) | 332 (7.1%) |  |
| UTAH | 486 (0.8%) | 86 (1.8%) |  |
| VIRGINIA | 1,325 (2.1%) | 78 (1.7%) |  |
| VIRGIN ISLANDS | 8 (<0.1%) | 0 (0%) |  |
| VERMONT | 76 (0.1%) | 7 (0.1%) |  |
| WASHINGTON | 897 (1.4%) | 102 (2.2%) |  |
| WISCONSIN | 1,332 (2.1%) | 151 (3.2%) |  |
| WEST VIRGINIA | 472 (0.8%) | 26 (0.6%) |  |
| WYOMING | 98 (0.2%) | 14 (0.3%) |  |
| UNKNOWN | 323 (0.5%) | 17 (0.4%) |  |
| **Donor RNA Nat serology value for htlv test** |  |  | <0.001 |
| Missing | 27,811 (44%) | 863 (18%) |  |
| Cannot Disclose | 1 (<0.1%) | 0 (0%) |  |
| Indeterminate | 5 (<0.1%) | 0 (0%) |  |
| Negative | 67 (0.1%) | 7 (0.1%) |  |
| Not Done | 34,617 (55%) | 3,821 (81%) |  |
| Positive | 1 (<0.1%) | 0 (0%) |  |
| Unknown | 8 (<0.1%) | 0 (0%) |  |
| **Donor history of hypertension (1= yes, 0= no)** |  |  | <0.001 |
| No | 39,915 (64%) | 3,412 (73%) |  |
| Yes | 21,967 (35%) | 1,242 (26%) |  |
| Missing | 628 (1.0%) | 37 (0.8%) |  |
| **Infection Source: Blood** |  |  | <0.001 |
| No | 55,970 (90%) | 4,061 (87%) |  |
| Yes | 6,540 (10%) | 630 (13%) |  |
| **Infection Source: Blood/Confirmed by Culture** |  |  | <0.001 |
| Missing | 60,245 (96%) | 4,613 (98%) |  |
| No | 389 (0.6%) | 1 (<0.1%) |  |
| Yes | 1,876 (3.0%) | 77 (1.6%) |  |
| **Infection Source: Lung** |  |  | <0.001 |
| No | 27,943 (45%) | 1,915 (41%) |  |
| Yes | 34,567 (55%) | 2,776 (59%) |  |
| **Infection Source: Lung/Confirmed by Culture** |  |  | <0.001 |
| Missing | 51,533 (82%) | 4,349 (93%) |  |
| No | 219 (0.4%) | 9 (0.2%) |  |
| Yes | 10,758 (17%) | 333 (7.1%) |  |
| **Infection Source: Other** |  |  | 0.024 |
| No | 58,084 (93%) | 4,315 (92%) |  |
| Yes | 4,426 (7.1%) | 376 (8.0%) |  |
| **Infection Source: Other/Confimed by Culture** |  |  | <0.001 |
| Missing | 60,700 (97%) | 4,641 (99%) |  |
| No | 53 (<0.1%) | 0 (0%) |  |
| Yes | 1,757 (2.8%) | 50 (1.1%) |  |
| **Infection Source: Urine** |  |  | <0.001 |
| No | 54,306 (87%) | 3,988 (85%) |  |
| Yes | 8,204 (13%) | 703 (15%) |  |
| **Infection Source: Urine/Confirmed by Culture** |  |  | <0.001 |
| Missing | 59,483 (95%) | 4,591 (98%) |  |
| No | 357 (0.6%) | 2 (<0.1%) |  |
| Yes | 2,670 (4.3%) | 98 (2.1%) |  |
| **Three or More Inotropic Agents at Time of Incision** |  |  | <0.001 |
| Missing | 34,509 (55%) | 3,817 (81%) |  |
| No | 27,281 (44%) | 873 (19%) |  |
| Yes | 720 (1.2%) | 1 (<0.1%) |  |
| **Inotropic Support** |  |  | <0.001 |
| Missing | 3 (<0.1%) | 0 (0%) |  |
| Negative / No | 30,985 (50%) | 4,500 (96%) |  |
| Positive / Yes | 31,384 (50%) | 189 (4.0%) |  |
| Unknown | 138 (0.2%) | 2 (<0.1%) |  |
| **Insulin:** |  |  | <0.001 |
| Missing | 1,020 (1.6%) | 0 (0%) |  |
| Negative / No | 25,326 (41%) | 3,727 (79%) |  |
| Positive / Yes | 36,104 (58%) | 959 (20%) |  |
| Unknown | 60 (<0.1%) | 5 (0.1%) |  |
| **Left Lung Machine Perfusion Intended or Performed:** |  |  | <0.001 |
| Missing | 47,207 (76%) | 3,550 (76%) |  |
| No | 14,742 (24%) | 1,023 (22%) |  |
| Yes | 561 (0.9%) | 118 (2.5%) |  |
| **Was the patient declared legally brain dead:** |  |  | <0.001 |
| Missing | 25,957 (42%) | 826 (18%) |  |
| No | 4 (<0.1%) | 3,774 (80%) |  |
| Yes | 36,549 (58%) | 91 (1.9%) |  |
| **Kidney Biopsy (left)** |  |  | <0.001 |
| Missing | 2,001 (3.2%) | 55 (1.2%) |  |
| No | 34,338 (55%) | 2,311 (49%) |  |
| Yes | 26,171 (42%) | 2,325 (50%) |  |
| **Pump (Left Kidney)** |  |  | <0.001 |
| Missing | 3,402 (5.4%) | 76 (1.6%) |  |
| No | 43,074 (69%) | 1,839 (39%) |  |
| Yes | 16,034 (26%) | 2,776 (59%) |  |
| **Transferred on pump (left)://Transferred on pump:** |  |  | <0.001 |
| Missing | 46,638 (75%) | 1,915 (41%) |  |
| No | 5,639 (9.0%) | 915 (20%) |  |
| Yes | 10,233 (16%) | 1,861 (40%) |  |
| **Left Lung Bronchoscopy:** |  |  | <0.001 |
| No Bronchoscopy | 2,496 (4.0%) | 435 (9.3%) |  |
| Bronchoscopy Results normal | 14,501 (23%) | 360 (7.7%) |  |
| Bronchoscopy Results, Abnormal-purulent secretions | 2,881 (4.6%) | 84 (1.8%) |  |
| Bronchoscopy Results, Abnormal-aspiration of foreign body | 165 (0.3%) | 4 (<0.1%) |  |
| Bronchoscopy Results, Abnormal-blood | 1,057 (1.7%) | 19 (0.4%) |  |
| Bronchoscopy Results, Abnormal-anatomy/other lesion | 299 (0.5%) | 15 (0.3%) |  |
| Bronchoscopy Results, Unknown | 131 (0.2%) | 5 (0.1%) |  |
| 8 | 35 (<0.1%) | 8 (0.2%) |  |
| Unknown if bronchoscopy performed | 36 (<0.1%) | 0 (0%) |  |
| Missing | 40,909 (65%) | 3,761 (80%) |  |
| **Liver Biopsy** |  |  | <0.001 |
| Missing | 53 (<0.1%) | 0 (0%) |  |
| No | 37,824 (61%) | 3,367 (72%) |  |
| Yes | 24,633 (39%) | 1,324 (28%) |  |
| **Granulomas Histology performed** |  |  | 0.5 |
| No | 26,657 (43%) | 872 (19%) |  |
| Yes | 66 (0.1%) | 1 (<0.1%) |  |
| Missing | 35,787 (57%) | 3,818 (81%) |  |
| **Hemosidera Histology performed** |  |  | 0.5 |
| No | 26,652 (43%) | 872 (19%) |  |
| Yes | 71 (0.1%) | 1 (<0.1%) |  |
| Missing | 35,787 (57%) | 3,818 (81%) |  |
| **Other Histology performed** |  |  | <0.001 |
| No | 23,655 (38%) | 812 (17%) |  |
| Yes | 3,068 (4.9%) | 61 (1.3%) |  |
| Missing | 35,787 (57%) | 3,818 (81%) |  |
| **Medical Examiner/Coroner** |  |  | <0.001 |
| Yes | 21,270 (34%) | 1,229 (26%) |  |
| YES, MEDICAL EXAMINER CONSENTED | 39,188 (63%) | 2,966 (63%) |  |
| YES, MEDICAL EXAMINER REFUSED CONSENT | 109 (0.2%) | 8 (0.2%) |  |
| UNKNOWN | 217 (0.3%) | 33 (0.7%) |  |
| Missing | 1,726 (2.8%) | 455 (9.7%) |  |
| **Does the Donor meet CDC guidelines for High Risk for an organ donor:** |  |  | <0.001 |
| Missing | 1,028 (1.6%) | 0 (0%) |  |
| Negative / No | 49,704 (80%) | 3,747 (80%) |  |
| Positive / Yes | 11,693 (19%) | 943 (20%) |  |
| Unknown | 85 (0.1%) | 1 (<0.1%) |  |
| **Non-Heart Beating Donor** |  |  | <0.001 |
| Missing | 0 (0%) | 0 (0%) |  |
| No | 61,970 (99%) | 0 (0%) |  |
| Yes | 540 (0.9%) | 4,691 (100%) |  |
| **Controlled** |  |  | <0.001 |
| Missing | 61,970 (99%) | 0 (0%) |  |
| Negative / No | 28 (<0.1%) | 0 (0%) |  |
| Positive / Yes | 507 (0.8%) | 4,691 (100%) |  |
| Unknown | 5 (<0.1%) | 0 (0%) |  |
| **Core Cooling Used** |  |  | <0.001 |
| Missing | 59,937 (96%) | 0 (0%) |  |
| No | 1,070 (1.7%) | 1,219 (26%) |  |
| Yes | 1,503 (2.4%) | 3,472 (74%) |  |
| **Organ was used in a shared 1 (vs local 0) TX** |  |  | 0.035 |
| No | 35,055 (56%) | 2,709 (58%) |  |
| Yes | 27,455 (44%) | 1,982 (42%) |  |
| **Lung - Was pO2 done:** |  |  | <0.001 |
| Missing | 35,365 (57%) | 3,817 (81%) |  |
| Negative / No | 629 (1.0%) | 23 (0.5%) |  |
| Positive / Yes | 26,456 (42%) | 850 (18%) |  |
| Unknown | 60 (<0.1%) | 1 (<0.1%) |  |
| **Pre-Recov Meds given Donor: Diuretics** |  |  | <0.001 |
| Missing | 3 (<0.1%) | 0 (0%) |  |
| Negative / No | 23,134 (37%) | 3,757 (80%) |  |
| Positive / Yes | 39,321 (63%) | 929 (20%) |  |
| Unknown | 52 (<0.1%) | 5 (0.1%) |  |
| **Pre-Recov Meds given Donor: Steroids** |  |  | <0.001 |
| Missing | 3 (<0.1%) | 0 (0%) |  |
| Negative / No | 16,214 (26%) | 3,758 (80%) |  |
| Positive / Yes | 46,226 (74%) | 927 (20%) |  |
| Unknown | 67 (0.1%) | 6 (0.1%) |  |
| **Pre-Recov Meds given Donor: T3** |  |  | 0.002 |
| Missing | 3 (<0.1%) | 0 (0%) |  |
| Negative / No | 62,172 (99%) | 4,688 (100%) |  |
| Positive / Yes | 286 (0.5%) | 0 (0%) |  |
| Unknown | 49 (<0.1%) | 3 (<0.1%) |  |
| **Pre-Recov Meds given Donor: T4** |  |  | <0.001 |
| Missing | 3 (<0.1%) | 0 (0%) |  |
| Negative / No | 26,059 (42%) | 4,384 (93%) |  |
| Positive / Yes | 36,415 (58%) | 304 (6.5%) |  |
| Unknown | 33 (<0.1%) | 3 (<0.1%) |  |
| **Previous Gastrointestinal Disease** |  |  | <0.001 |
| Missing | 726 (1.2%) | 3 (<0.1%) |  |
| Negative / No | 55,428 (89%) | 4,382 (93%) |  |
| Positive / Yes | 3,914 (6.3%) | 269 (5.7%) |  |
| Unknown | 2,442 (3.9%) | 37 (0.8%) |  |
| **Protein in Urine** |  |  | <0.001 |
| Missing | 2 (<0.1%) | 0 (0%) |  |
| Negative / No | 31,307 (50%) | 1,906 (41%) |  |
| Positive / Yes | 30,311 (48%) | 2,718 (58%) |  |
| Unknown | 890 (1.4%) | 67 (1.4%) |  |
| **Was a pulmonary artery catheter placed:** |  |  | <0.001 |
| Missing | 2,427 (3.9%) | 460 (9.8%) |  |
| No | 55,769 (89%) | 4,195 (89%) |  |
| Yes | 4,314 (6.9%) | 36 (0.8%) |  |
| **SRTR Donor Race** |  |  | <0.001 |
| Asian | 1,458 (2.3%) | 84 (1.8%) |  |
| Black | 11,762 (19%) | 478 (10%) |  |
| Multiracial | 192 (0.3%) | 13 (0.3%) |  |
| Native American | 331 (0.5%) | 32 (0.7%) |  |
| Pacific Islander | 157 (0.3%) | 9 (0.2%) |  |
| White | 48,610 (78%) | 4,075 (87%) |  |
| **Recovered Outside the U.S.** |  |  | <0.001 |
| N | 62,150 (99%) | 4,691 (100%) |  |
| Y | 360 (0.6%) | 0 (0%) |  |
| **Duration of Resuscitation** |  |  | 0.5 |
| 1 | 78 (0.1%) | 1 (<0.1%) |  |
| 10 | 282 (0.5%) | 2 (<0.1%) |  |
| 11 | 57 (<0.1%) | 0 (0%) |  |
| 112 | 1 (<0.1%) | 0 (0%) |  |
| 115 | 1 (<0.1%) | 0 (0%) |  |
| 12 | 73 (0.1%) | 1 (<0.1%) |  |
| 120 | 7 (<0.1%) | 0 (0%) |  |
| 13 | 38 (<0.1%) | 0 (0%) |  |
| 14 | 43 (<0.1%) | 1 (<0.1%) |  |
| 15 | 246 (0.4%) | 3 (<0.1%) |  |
| 16 | 44 (<0.1%) | 0 (0%) |  |
| 17 | 20 (<0.1%) | 0 (0%) |  |
| 18 | 35 (<0.1%) | 0 (0%) |  |
| 19 | 13 (<0.1%) | 0 (0%) |  |
| 2 | 165 (0.3%) | 0 (0%) |  |
| 20 | 296 (0.5%) | 0 (0%) |  |
| 21 | 25 (<0.1%) | 0 (0%) |  |
| 22 | 43 (<0.1%) | 0 (0%) |  |
| 23 | 28 (<0.1%) | 0 (0%) |  |
| 24 | 22 (<0.1%) | 1 (<0.1%) |  |
| 25 | 110 (0.2%) | 2 (<0.1%) |  |
| 26 | 18 (<0.1%) | 0 (0%) |  |
| 27 | 18 (<0.1%) | 0 (0%) |  |
| 28 | 20 (<0.1%) | 0 (0%) |  |
| 29 | 18 (<0.1%) | 0 (0%) |  |
| 3 | 133 (0.2%) | 0 (0%) |  |
| 30 | 250 (0.4%) | 6 (0.1%) |  |
| 31 | 14 (<0.1%) | 1 (<0.1%) |  |
| 32 | 15 (<0.1%) | 1 (<0.1%) |  |
| 33 | 24 (<0.1%) | 0 (0%) |  |
| 34 | 15 (<0.1%) | 0 (0%) |  |
| 35 | 72 (0.1%) | 1 (<0.1%) |  |
| 36 | 20 (<0.1%) | 0 (0%) |  |
| 37 | 19 (<0.1%) | 0 (0%) |  |
| 38 | 7 (<0.1%) | 0 (0%) |  |
| 39 | 12 (<0.1%) | 0 (0%) |  |
| 4 | 120 (0.2%) | 1 (<0.1%) |  |
| 40 | 107 (0.2%) | 2 (<0.1%) |  |
| 41 | 5 (<0.1%) | 0 (0%) |  |
| 42 | 5 (<0.1%) | 0 (0%) |  |
| 43 | 11 (<0.1%) | 0 (0%) |  |
| 44 | 9 (<0.1%) | 0 (0%) |  |
| 45 | 107 (0.2%) | 0 (0%) |  |
| 46 | 9 (<0.1%) | 0 (0%) |  |
| 47 | 8 (<0.1%) | 0 (0%) |  |
| 48 | 10 (<0.1%) | 0 (0%) |  |
| 49 | 6 (<0.1%) | 0 (0%) |  |
| 5 | 202 (0.3%) | 1 (<0.1%) |  |
| 50 | 42 (<0.1%) | 0 (0%) |  |
| 51 | 5 (<0.1%) | 0 (0%) |  |
| 52 | 3 (<0.1%) | 0 (0%) |  |
| 53 | 5 (<0.1%) | 0 (0%) |  |
| 54 | 5 (<0.1%) | 0 (0%) |  |
| 55 | 14 (<0.1%) | 1 (<0.1%) |  |
| 56 | 2 (<0.1%) | 0 (0%) |  |
| 57 | 3 (<0.1%) | 0 (0%) |  |
| 58 | 3 (<0.1%) | 1 (<0.1%) |  |
| 59 | 4 (<0.1%) | 0 (0%) |  |
| 6 | 91 (0.1%) | 1 (<0.1%) |  |
| 60 | 86 (0.1%) | 1 (<0.1%) |  |
| 61 | 1 (<0.1%) | 0 (0%) |  |
| 62 | 6 (<0.1%) | 0 (0%) |  |
| 63 | 5 (<0.1%) | 0 (0%) |  |
| 65 | 6 (<0.1%) | 0 (0%) |  |
| 68 | 2 (<0.1%) | 0 (0%) |  |
| 69 | 3 (<0.1%) | 0 (0%) |  |
| 7 | 59 (<0.1%) | 1 (<0.1%) |  |
| 70 | 11 (<0.1%) | 0 (0%) |  |
| 71 | 1 (<0.1%) | 0 (0%) |  |
| 72 | 1 (<0.1%) | 0 (0%) |  |
| 73 | 1 (<0.1%) | 0 (0%) |  |
| 75 | 7 (<0.1%) | 0 (0%) |  |
| 77 | 1 (<0.1%) | 0 (0%) |  |
| 8 | 81 (0.1%) | 3 (<0.1%) |  |
| 80 | 4 (<0.1%) | 0 (0%) |  |
| 81 | 1 (<0.1%) | 0 (0%) |  |
| 82 | 1 (<0.1%) | 0 (0%) |  |
| 83 | 1 (<0.1%) | 0 (0%) |  |
| 85 | 1 (<0.1%) | 0 (0%) |  |
| 86 | 1 (<0.1%) | 0 (0%) |  |
| 88 | 3 (<0.1%) | 0 (0%) |  |
| 89 | 1 (<0.1%) | 0 (0%) |  |
| 9 | 54 (<0.1%) | 0 (0%) |  |
| 90 | 15 (<0.1%) | 0 (0%) |  |
| 95 | 1 (<0.1%) | 0 (0%) |  |
| 97 | 1 (<0.1%) | 0 (0%) |  |
| 98 | 1 (<0.1%) | 0 (0%) |  |
| 99 | 4 (<0.1%) | 0 (0%) |  |
| Missing | 59,092 (95%) | 4,659 (99%) |  |
| **Right Lung Machine Perfusion Intended or Performed** |  |  | <0.001 |
| Missing | 47,360 (76%) | 3,547 (76%) |  |
| No | 14,601 (23%) | 1,025 (22%) |  |
| Yes | 549 (0.9%) | 119 (2.5%) |  |
| **RPR-VDRL** |  |  | 0.010 |
| Missing | 1 (<0.1%) | 0 (0%) |  |
| Indeterminate | 5 (<0.1%) | 0 (0%) |  |
| Negative | 61,174 (98%) | 4,626 (99%) |  |
| Not Done | 657 (1.1%) | 17 (0.4%) |  |
| Positive | 661 (1.1%) | 48 (1.0%) |  |
| Unknown | 12 (<0.1%) | 0 (0%) |  |
| **Kidney Biopsy (right)** |  |  | <0.001 |
| Missing | 2,032 (3.3%) | 54 (1.2%) |  |
| No | 33,885 (54%) | 2,271 (48%) |  |
| Yes | 26,593 (43%) | 2,366 (50%) |  |
| **Pump (Right Kidney)** |  |  | <0.001 |
| Missing | 3,417 (5.5%) | 73 (1.6%) |  |
| No | 42,828 (69%) | 1,786 (38%) |  |
| Yes | 16,265 (26%) | 2,832 (60%) |  |
| **Transferred on pump (right)://Transferred on pump:** |  |  | <0.001 |
| Missing | 46,404 (74%) | 1,859 (40%) |  |
| No | 5,887 (9.4%) | 946 (20%) |  |
| Yes | 10,219 (16%) | 1,886 (40%) |  |
| **Right Lung Bronchoscopy:** |  |  | <0.001 |
| No Bronchoscopy | 2,558 (4.1%) | 443 (9.4%) |  |
| Bronchoscopy Results normal | 13,770 (22%) | 346 (7.4%) |  |
| Bronchoscopy Results, Abnormal-purulent secretions | 3,216 (5.1%) | 94 (2.0%) |  |
| Bronchoscopy Results, Abnormal-aspiration of foreign body | 232 (0.4%) | 6 (0.1%) |  |
| Bronchoscopy Results, Abnormal-blood | 1,119 (1.8%) | 19 (0.4%) |  |
| Bronchoscopy Results, Abnormal-anatomy/other lesion | 334 (0.5%) | 15 (0.3%) |  |
| Bronchoscopy Results, Unknown | 137 (0.2%) | 5 (0.1%) |  |
| 8 | 37 (<0.1%) | 10 (0.2%) |  |
| Unknown if bronchoscopy performed | 37 (<0.1%) | 1 (<0.1%) |  |
| Missing | 41,070 (66%) | 3,752 (80%) |  |
| **strongyloides test result selection** |  |  | <0.001 |
| Missing | 42,942 (69%) | 2,035 (43%) |  |
| Indeterminate | 9 (<0.1%) | 2 (<0.1%) |  |
| Negative | 3,588 (5.7%) | 417 (8.9%) |  |
| Not Done | 15,765 (25%) | 2,220 (47%) |  |
| Pending | 88 (0.1%) | 5 (0.1%) |  |
| Positive | 118 (0.2%) | 12 (0.3%) |  |
| **Lifestyle Factors: Tattoos** |  |  | <0.001 |
| Missing | 1,816 (2.9%) | 460 (9.8%) |  |
| Negative / No | 35,511 (57%) | 2,046 (44%) |  |
| Positive / Yes | 25,047 (40%) | 2,176 (46%) |  |
| Unknown | 136 (0.2%) | 9 (0.2%) |  |
| **Is there time for crossmatch?** |  |  | <0.001 |
| Missing | 49,737 (80%) | 4,567 (97%) |  |
| No | 574 (0.9%) | 1 (<0.1%) |  |
| Yes | 12,199 (20%) | 123 (2.6%) |  |
| **Number of transfusions during this (terminal) hospitalization:** |  |  | <0.001 |
| NONE | 35,266 (56%) | 3,142 (67%) |  |
| 1 - 5 | 18,563 (30%) | 1,140 (24%) |  |
| 6 - 10 | 4,640 (7.4%) | 243 (5.2%) |  |
| GREATER THAN 10 | 2,950 (4.7%) | 140 (3.0%) |  |
| UNKNOWN | 30 (<0.1%) | 0 (0%) |  |
| Missing | 1,061 (1.7%) | 26 (0.6%) |  |
| **Donor/s HLA - Typing Method Class I** |  |  | <0.001 |
| Serology | 5,046 (8.1%) | 89 (1.9%) |  |
| DNA | 48,845 (78%) | 4,304 (92%) |  |
| 3 | 7,228 (12%) | 247 (5.3%) |  |
| Missing | 1,391 (2.2%) | 51 (1.1%) |  |
| **Donor/s HLA - Typing Method Class II** |  |  | <0.001 |
| Serology | 3,737 (6.0%) | 77 (1.6%) |  |
| DNA | 50,952 (82%) | 4,341 (93%) |  |
| 3 | 6,430 (10%) | 222 (4.7%) |  |
| Missing | 1,391 (2.2%) | 51 (1.1%) |  |
| **Use Double KI allocation, not Expanded Donor** |  |  | <0.001 |
| Missing | 61,660 (99%) | 4,684 (100%) |  |
| No | 367 (0.6%) | 2 (<0.1%) |  |
| Yes | 483 (0.8%) | 5 (0.1%) |  |
| **Vasodilators** |  |  | <0.001 |
| Missing | 4 (<0.1%) | 0 (0%) |  |
| Negative / No | 52,502 (84%) | 4,134 (88%) |  |
| Positive / Yes | 9,946 (16%) | 549 (12%) |  |
| Unknown | 58 (<0.1%) | 8 (0.2%) |  |
| **Wall Abnormalities //Global:** |  |  | <0.001 |
| Missing | 55,481 (89%) | 4,335 (92%) |  |
| No | 4,191 (6.7%) | 217 (4.6%) |  |
| Yes | 2,838 (4.5%) | 139 (3.0%) |  |
| **Wall Abnormalities //Segmental:** |  |  | <0.001 |
| Missing | 55,489 (89%) | 4,335 (92%) |  |
| No | 4,634 (7.4%) | 243 (5.2%) |  |
| Yes | 2,387 (3.8%) | 113 (2.4%) |  |
| **Donor RNA Nat serology value for West Nile test** |  |  | <0.001 |
| Missing | 27,811 (44%) | 863 (18%) |  |
| Cannot Disclose | 3 (<0.1%) | 0 (0%) |  |
| Indeterminate | 2 (<0.1%) | 1 (<0.1%) |  |
| Negative | 12,065 (19%) | 1,537 (33%) |  |
| Not Done | 22,622 (36%) | 2,290 (49%) |  |
| Positive | 2 (<0.1%) | 0 (0%) |  |
| Unknown | 5 (<0.1%) | 0 (0%) |  |
| **^1^Median (Q1, Q3); n (%)** | | | |
| **^2^Wilcoxon rank sum test; Pearson's Chi-squared test with simulated p-value (based on 2000 replicates)** | | | |

# **Table D2. Donor Characteristics by Donor Type, 2004-01-01 to 2012-12-31**

| **Characteristic** | **DBD**  N = 22,693^1^ | **DCD**  N = 587^1^ | **p-value**^2^ |
| --- | --- | --- | --- |
| **Calculated Donor Age in Years at Organ Recovery or Referral Date** | 43.00 (26.00, 55.00) | 31.00 (22.00, 45.00) | <0.001 |
| **Donor/s Age in Months** | 518.00 (314.00, 661.00) | 379.00 (275.00, 540.00) | <0.001 |
| **most recent serum creatinine** | 1.10 (0.80, 1.50) | 0.83 (0.60, 1.20) | <0.001 |
| **BUN** | 15.00 (10.00, 22.00) | 15.00 (10.00, 22.00) | 0.6 |
| **Cancer Free Interval:** | 5.00 (0.00, 13.00) | 0.00 (0.00, 7.50) | 0.034 |
| **Cardiac Index (final) //Cardiac Index:** | 3.80 (3.10, 4.70) | 5.10 (4.30, 5.20) | 0.12 |
| **Cardiac Index (initial) //Cardiac Index: (L/min/sq.m)** | 3.37 (2.60, 4.20) | 4.10 (4.00, 4.30) | 0.3 |
| **CO (final) (L/min) //CO:** | 7.20 (5.80, 8.90) | 10.50 (9.10, 11.30) | 0.038 |
| **CO (initial) (L/min) //CO: (L/min)** | 6.30 (5.00, 8.10) | 8.40 (8.00, 9.90) | 0.2 |
| **Serum Creatinine** | 1.10 (0.80, 1.50) | 0.80 (0.60, 1.20) | <0.001 |
| **CVP (final) //CVP:** | 9.00 (7.00, 12.00) | 8.00 (8.00, 10.00) | 0.7 |
| **CVP (initial) //CVP: (mm/Hg)** | 8.00 (6.00, 11.00) | 9.00 (6.00, 10.00) | 0.5 |
| **LV Ejection Fraction %** | 60.00 (51.00, 65.00) | 60.00 (45.00, 65.00) | 0.3 |
| **Final Serum Creatinine** | 1.10 (0.80, 1.53) | 0.83 (0.60, 1.20) | <0.001 |
| **Hematocrit:** | 31.00 (27.80, 35.20) | 30.05 (26.20, 34.40) | <0.001 |
| **Donor/s Height in centimeters** | 172.72 (165.00, 180.00) | 175.00 (167.00, 182.00) | <0.001 |
| **INR:** | 1.27 (1.10, 1.40) | 1.20 (1.10, 1.40) | <0.001 |
| **Glomeruli Observed on Kidney biopsy** | 42.50 (28.00, 59.00) | 26.00 (8.00, 60.00) | 0.4 |
| **Percent Glomerular Sclerosis on Kidney biopsy** | 12.50 (2.00, 24.00) | 0.00 (0.00, 2.00) | 0.040 |
| **% Glomerulosclerosis (Left Kidney)** | 1.00 (1.00, 2.00) | 1.00 (1.00, 2.00) | <0.001 |
| **Final Resistance Prior to Shipping (left) //Final Resistance Prior to Shipping:** | 0.22 (0.17, 0.30) | 0.21 (0.15, 0.28) | 0.003 |
| **Serum Lipase** | 24.00 (14.00, 68.85) | 36.00 (18.00, 129.00) | 0.001 |
| **% Macro vesicular fat:** | 5.00 (0.00, 10.00) | 4.00 (0.00, 10.00) | 0.060 |
| **% Micro vesicular fat:** | 5.00 (0.00, 10.00) | 1.00 (0.00, 5.00) | 0.045 |
| **MAP (final) //Map:** | 85.00 (75.00, 97.00) | 80.00 (68.00, 97.00) | 0.6 |
| **MAP (initial) //Map: (mm/Hg)** | 83.00 (73.00, 95.00) | 83.00 (74.00, 97.00) | >0.9 |
| **Maximum Donor Creatinine** | 1.10 (0.80, 1.60) | 0.90 (0.60, 1.20) | <0.001 |
| **PA Diastolic (final) //PA Diastolic:** | 17.00 (13.00, 21.00) | 18.50 (16.00, 26.00) | 0.2 |
| **PA Diastolic (initial) //PA Diastolic: (mm/Hg)** | 16.00 (12.00, 20.00) | 13.00 (11.00, 19.00) | 0.3 |
| **PA Systolic (final) //PA Systolic:** | 28.00 (23.00, 35.00) | 30.00 (28.00, 34.00) | 0.3 |
| **PA Systolic (initial) //PA Systolic: (mm/Hg)** | 28.00 (23.00, 34.00) | 30.50 (22.00, 33.00) | >0.9 |
| **pCO2:** | 36.10 (32.00, 41.00) | 38.00 (33.00, 43.30) | <0.001 |
| **PCWP (final) //PCWP:** | 12.00 (9.00, 15.00) | 10.00 (10.00, 12.00) | 0.5 |
| **PCWP (initial) //PCWP: (mm/Hg)** | 12.00 (9.00, 16.00) | 12.00 (9.00, 13.00) | 0.7 |
| **Peak Serum Creatinine** | 1.33 (1.00, 1.90) | 1.20 (0.90, 1.56) | <0.001 |
| **Blood PH:** | 7.40 (7.36, 7.45) | 7.42 (7.37, 7.47) | <0.001 |
| **Lung pO2 on 100%** | 181.00 (114.00, 369.00) | 126.00 (90.00, 201.00) | <0.001 |
| **Lung pO2 on Fio2 //If Yes, Lung pO2 on FiO2 of:** | 100.00 (50.00, 100.00) | 60.00 (40.00, 100.00) | <0.001 |
| **% Glomerulosclerosis (Right Kidney)** | 1.00 (1.00, 2.00) | 1.00 (1.00, 2.00) | <0.001 |
| **Final Resistance Prior to Shipping (right) //Final Resistance Prior to Shipping:** | 0.21 (0.16, 0.28) | 0.20 (0.16, 0.28) | 0.2 |
| **Serum Amylase** | 64.00 (34.00, 132.00) | 55.50 (30.00, 119.00) | 0.2 |
| **SGOT/AST** | 43.00 (27.00, 80.00) | 53.00 (33.00, 84.00) | <0.001 |
| **SGPT/ALT** | 33.00 (21.00, 60.00) | 38.00 (24.00, 70.00) | <0.001 |
| **Serum Sodium //Last Serum Sodium Prior to Procurement:** | 147.00 (141.00, 153.00) | 146.00 (141.00, 152.00) | 0.14 |
| **SVR (final) //SVR:** | 872.00 (693.00, 1,150.00) | 826.00 (826.00, 826.00) | 0.8 |
| **SVR (initial) //SVR: (dynes/sec/cm)^5)** | 1,003.00 (744.00, 1,342.00) | 826.00 (487.00, 831.00) | 0.11 |
| **Total Bilirubin** | 0.70 (0.50, 1.10) | 0.60 (0.40, 0.90) | <0.001 |
| **Troponin I** | 0.20 (0.05, 1.00) | 0.14 (0.05, 1.47) | 0.3 |
| **Troponin T** | 0.07 (0.01, 0.33) | 0.08 (0.03, 0.52) | 0.14 |
| **If Abnormal, # of Vessels with > 50% Stenosis** | 1.00 (0.00, 2.00) | 1.00 (1.00, 1.00) | 0.3 |
| **Estimated Warm Ischemic Time in minutes** | 7.00 (0.00, 15.00) | 15.00 (10.00, 22.00) | <0.001 |
| **Donor/s Weight in kilograms** | 77.80 (66.40, 90.72) | 78.00 (67.00, 91.00) | 0.9 |
| **Donor Risk Index (DRI)** | 1.66 (1.44, 1.97) | 2.23 (2.05, 2.57) | <0.001 |
| **Donor/s Blood Type** |  |  | <0.001 |
| A | 3,570 (16%) | 70 (12%) |  |
| A1 | 4,277 (19%) | 158 (27%) |  |
| A1B | 321 (1.4%) | 3 (0.5%) |  |
| A2 | 552 (2.4%) | 20 (3.4%) |  |
| A2B | 93 (0.4%) | 3 (0.5%) |  |
| AB | 442 (1.9%) | 3 (0.5%) |  |
| B | 2,639 (12%) | 51 (8.7%) |  |
| O | 10,799 (48%) | 279 (48%) |  |
| **CVA cause of death** |  |  | <0.001 |
| No | 12,059 (53%) | 482 (82%) |  |
| Yes | 8,885 (39%) | 105 (18%) |  |
| Missing | 1,749 (8%) | 0 (0%) |  |
| **history of hypertension** |  |  | <0.001 |
| No | 13,113 (58%) | 456 (78%) |  |
| Yes | 7,423 (33%) | 129 (22%) |  |
| Missing | 2,157 (9.5%) | 2 (0.3%) |  |
| **Allocate Remaining Liver Segment?** |  |  | 0.8 |
| Missing | 22,606 (100%) | 586 (100%) |  |
| No | 51 (0.2%) | 1 (0.2%) |  |
| Yes | 36 (0.2%) | 0 (0%) |  |
| **Anti-CMV** |  |  | <0.001 |
| Missing | 1 (<0.1%) | 0 (0%) |  |
| Indeterminate | 35 (0.2%) | 5 (0.9%) |  |
| Negative | 7,612 (34%) | 294 (50%) |  |
| Not Done | 12 (<0.1%) | 0 (0%) |  |
| Positive | 15,001 (66%) | 288 (49%) |  |
| Unknown | 32 (0.1%) | 0 (0%) |  |
| **Anti-HBC** |  |  | 0.2 |
| Indeterminate | 3 (<0.1%) | 0 (0%) |  |
| Negative | 21,511 (95%) | 572 (97%) |  |
| Not Done | 33 (0.1%) | 0 (0%) |  |
| Positive | 1,142 (5.0%) | 15 (2.6%) |  |
| Unknown | 4 (<0.1%) | 0 (0%) |  |
| **Anti-HCV** |  |  | 0.3 |
| Missing | 0 (0%) | 0 (0%) |  |
| Cannot Disclose | 1 (<0.1%) | 0 (0%) |  |
| Indeterminate | 5 (<0.1%) | 0 (0%) |  |
| Negative | 21,959 (97%) | 579 (99%) |  |
| Not Done | 31 (0.1%) | 0 (0%) |  |
| Positive | 692 (3.0%) | 8 (1.4%) |  |
| Unknown | 5 (<0.1%) | 0 (0%) |  |
| **Anti-HIV I/II:** |  |  | >0.9 |
| Missing | 1 (<0.1%) | 0 (0%) |  |
| Negative | 22,678 (100%) | 587 (100%) |  |
| Not Done | 4 (<0.1%) | 0 (0%) |  |
| Positive | 1 (<0.1%) | 0 (0%) |  |
| Unknown | 9 (<0.1%) | 0 (0%) |  |
| **Anti-HTLV I/II:** |  |  | <0.001 |
| Missing | 1 (<0.1%) | 0 (0%) |  |
| Cannot Disclose | 1 (<0.1%) | 0 (0%) |  |
| Indeterminate | 1 (<0.1%) | 0 (0%) |  |
| Negative | 17,044 (75%) | 339 (58%) |  |
| Not Done | 5,573 (25%) | 248 (42%) |  |
| Positive | 61 (0.3%) | 0 (0%) |  |
| Unknown | 12 (<0.1%) | 0 (0%) |  |
| **Arginine Vasopressin:** |  |  | <0.001 |
| Missing | 1,017 (4.5%) | 0 (0%) |  |
| Negative / No | 9,975 (44%) | 470 (80%) |  |
| Positive / Yes | 11,639 (51%) | 113 (19%) |  |
| Unknown | 62 (0.3%) | 4 (0.7%) |  |
| **Donor/s Cause of Death** |  |  | <0.001 |
| Anoxia | 4,157 (18%) | 221 (38%) |  |
| Cerebrovascular / Stroke | 9,530 (42%) | 104 (18%) |  |
| CNS Tumor | 134 (0.6%) | 1 (0.2%) |  |
| Head Trauma | 8,449 (37%) | 236 (40%) |  |
| Other | 422 (1.9%) | 25 (4.3%) |  |
| Missing | 1 (<0.1%) | 0 (0%) |  |
| **Cardiac Arr. Since Event that led to Decl of Death** |  |  | <0.001 |
| Missing | 161 (0.7%) | 164 (28%) |  |
| No | 21,087 (93%) | 402 (68%) |  |
| Yes | 1,445 (6.4%) | 21 (3.6%) |  |
| **Donor RNA Nat serology value for Chagas test** |  |  | >0.9 |
| Missing | 22,686 (100%) | 587 (100%) |  |
| Not Done | 6 (<0.1%) | 0 (0%) |  |
| Unknown | 1 (<0.1%) | 0 (0%) |  |
| **Chest X-ray:** |  |  | <0.001 |
| No chest x-ray | 348 (1.5%) | 14 (2.4%) |  |
| Normal | 5,685 (25%) | 114 (19%) |  |
| Abnormal-left | 1,970 (8.7%) | 47 (8.0%) |  |
| Abnormal-right | 2,021 (8.9%) | 49 (8.3%) |  |
| Abnormal-both | 7,220 (32%) | 264 (45%) |  |
| Results Unknown | 95 (0.4%) | 0 (0%) |  |
| Unknown if chest x-ray performed | 228 (1.0%) | 0 (0%) |  |
| Missing | 5,126 (23%) | 99 (17%) |  |
| **Donor/s Citizenship** |  |  | 0.3 |
| US Citizen | 22,206 (98%) | 582 (99%) |  |
| Resident Alien | 284 (1.3%) | 5 (0.9%) |  |
| Non-Resident Alien, Specify Country | 114 (0.5%) | 0 (0%) |  |
| Non-US Citizen/US Resident | 79 (0.3%) | 0 (0%) |  |
| Non-US Citizen/Non-US Resident | 6 (<0.1%) | 0 (0%) |  |
| Unknown | 4 (<0.1%) | 0 (0%) |  |
| **Clamp Time Zone** |  |  | <0.001 |
| Eastern | 10,941 (48%) | 289 (49%) |  |
| Central | 7,375 (32%) | 213 (36%) |  |
| Mountain | 1,127 (5.0%) | 35 (6.0%) |  |
| Pacific | 2,708 (12%) | 48 (8.2%) |  |
| Alaska | 47 (0.2%) | 2 (0.3%) |  |
| Hawaii | 97 (0.4%) | 0 (0%) |  |
| Atlantic | 397 (1.7%) | 0 (0%) |  |
| Missing | 1 (<0.1%) | 0 (0%) |  |
| **Clinical Infection** |  |  | 0.4 |
| Missing | 3 (<0.1%) | 0 (0%) |  |
| Negative / No | 12,311 (54%) | 319 (54%) |  |
| Positive / Yes | 9,759 (43%) | 246 (42%) |  |
| Unknown | 620 (2.7%) | 22 (3.7%) |  |
| **Donor cause of death is stroke (1= yes, 0= no)** |  |  | <0.001 |
| No | 12,867 (57%) | 479 (82%) |  |
| Yes | 9,826 (43%) | 108 (18%) |  |
| **Alcohol Dependency Last 6 Months** |  |  | 0.14 |
| Missing | 22,464 (99%) | 587 (100%) |  |
| Negative / No | 60 (0.3%) | 0 (0%) |  |
| Positive / Yes | 165 (0.7%) | 0 (0%) |  |
| Unknown | 4 (<0.1%) | 0 (0%) |  |
| **Donor/s Continued Use in Last 6 Months** |  |  | <0.001 |
| Missing | 16,177 (71%) | 480 (82%) |  |
| Negative / No | 1,182 (5.2%) | 9 (1.5%) |  |
| Positive / Yes | 5,302 (23%) | 95 (16%) |  |
| Unknown | 32 (0.1%) | 3 (0.5%) |  |
| **Cocaine Use Last 6 Months** |  |  | <0.001 |
| Missing | 19,731 (87%) | 491 (84%) |  |
| Negative / No | 1,076 (4.7%) | 39 (6.6%) |  |
| Positive / Yes | 1,503 (6.6%) | 34 (5.8%) |  |
| Unknown | 383 (1.7%) | 23 (3.9%) |  |
| **IV Drug Last 6 Months** |  |  | >0.9 |
| Missing | 22,657 (100%) | 587 (100%) |  |
| Negative / No | 14 (<0.1%) | 0 (0%) |  |
| Positive / Yes | 14 (<0.1%) | 0 (0%) |  |
| Unknown | 8 (<0.1%) | 0 (0%) |  |
| **Other Drug Abuse Last 6 Months** |  |  | <0.001 |
| Missing | 15,817 (70%) | 352 (60%) |  |
| Negative / No | 2,024 (8.9%) | 62 (11%) |  |
| Positive / Yes | 4,135 (18%) | 148 (25%) |  |
| Unknown | 717 (3.2%) | 25 (4.3%) |  |
| **Coronary Angiogram** |  |  | <0.001 |
| No | 19,239 (85%) | 580 (99%) |  |
| Yes, normal | 2,013 (8.9%) | 2 (0.3%) |  |
| Yes, not normal | 829 (3.7%) | 5 (0.9%) |  |
| Missing | 612 (2.7%) | 0 (0%) |  |
| **If not DCD, Was this a consented DCD donor that progressed to brain death?** |  |  | <0.001 |
| Missing | 10,494 (46%) | 587 (100%) |  |
| No | 11,909 (52%) | 0 (0%) |  |
| Yes | 290 (1.3%) | 0 (0%) |  |
| **Meds given Donor: DDAVP** |  |  | 0.001 |
| Missing | 2 (<0.1%) | 0 (0%) |  |
| Negative / No | 18,597 (82%) | 540 (92%) |  |
| Positive / Yes | 4,058 (18%) | 43 (7.3%) |  |
| Unknown | 36 (0.2%) | 4 (0.7%) |  |
| **Cirumstances of Death** |  |  | <0.001 |
| MVA | 4,280 (19%) | 155 (26%) |  |
| SUICIDE | 2,025 (8.9%) | 94 (16%) |  |
| HOMICIDE | 1,501 (6.6%) | 23 (3.9%) |  |
| CHILD-ABUSE | 3 (<0.1%) | 0 (0%) |  |
| NON-MVA | 1,910 (8.4%) | 55 (9.4%) |  |
| DEATH FROM NATURAL CAUSES | 8,299 (37%) | 154 (26%) |  |
| NONE OF THE ABOVE | 4,674 (21%) | 106 (18%) |  |
| Missing | 1 (<0.1%) | 0 (0%) |  |
| **Mechanism of Death** |  |  | <0.001 |
| DROWNING | 109 (0.5%) | 9 (1.5%) |  |
| SEIZURE | 163 (0.7%) | 6 (1.0%) |  |
| DRUG INTOXICATION | 909 (4.0%) | 28 (4.8%) |  |
| ASPHYXIATION | 544 (2.4%) | 59 (10%) |  |
| CARDIOVASCULAR | 2,301 (10%) | 109 (19%) |  |
| ELECTRICAL | 9 (<0.1%) | 0 (0%) |  |
| GUNSHOT WOUND | 2,519 (11%) | 58 (9.9%) |  |
| STAB | 48 (0.2%) | 1 (0.2%) |  |
| BLUNT INJURY | 5,462 (24%) | 170 (29%) |  |
| INTRACRANIAL HEMORRHAGE/STROKE | 9,769 (43%) | 115 (20%) |  |
| DEATH FROM NATURAL CAUSES | 323 (1.4%) | 11 (1.9%) |  |
| NONE OF THE ABOVE | 536 (2.4%) | 21 (3.6%) |  |
| Missing | 1 (<0.1%) | 0 (0%) |  |
| **EBNA (Epstein-Barr nuclear antigen)** |  |  | <0.001 |
| Missing | 5,651 (25%) | 0 (0%) |  |
| Cannot Disclose | 21 (<0.1%) | 0 (0%) |  |
| Indeterminate | 39 (0.2%) | 2 (0.3%) |  |
| Negative | 537 (2.4%) | 31 (5.3%) |  |
| Not Done | 12,379 (55%) | 417 (71%) |  |
| Positive | 3,909 (17%) | 134 (23%) |  |
| Unknown | 157 (0.7%) | 3 (0.5%) |  |
| **EBV//IgG:** |  |  | <0.001 |
| Missing | 5,568 (25%) | 0 (0%) |  |
| Cannot Disclose | 4 (<0.1%) | 0 (0%) |  |
| Indeterminate | 38 (0.2%) | 4 (0.7%) |  |
| Negative | 790 (3.5%) | 40 (6.8%) |  |
| Not Done | 2,497 (11%) | 33 (5.6%) |  |
| Positive | 13,621 (60%) | 509 (87%) |  |
| Unknown | 175 (0.8%) | 1 (0.2%) |  |
| **EBV//IgM:** |  |  | <0.001 |
| Missing | 5,568 (25%) | 0 (0%) |  |
| Cannot Disclose | 8 (<0.1%) | 0 (0%) |  |
| Indeterminate | 38 (0.2%) | 2 (0.3%) |  |
| Negative | 12,906 (57%) | 484 (82%) |  |
| Not Done | 3,629 (16%) | 88 (15%) |  |
| Positive | 379 (1.7%) | 12 (2.0%) |  |
| Unknown | 165 (0.7%) | 1 (0.2%) |  |
| **Method** |  |  | 0.13 |
| Echo | 14,186 (63%) | 50 (8.5%) |  |
| MUGA | 4 (<0.1%) | 0 (0%) |  |
| Angiogram | 312 (1.4%) | 3 (0.5%) |  |
| Missing | 8,191 (36%) | 534 (91%) |  |
| **SRTR Donor Ethnicity** |  |  | <0.001 |
| Latino | 3,077 (14%) | 32 (5.5%) |  |
| Non-Latino or unknown | 19,616 (86%) | 555 (95%) |  |
| **Does donor meet criteria to be an Expanded Donor?** |  |  | <0.001 |
| No | 17,130 (75%) | 563 (96%) |  |
| Yes | 5,542 (24%) | 24 (4.1%) |  |
| Missing | 21 (<0.1%) | 0 (0%) |  |
| **Meets expanded donor criteria for kidney (1= yes , 0= no)** |  |  | <0.001 |
| No | 16,616 (73%) | 562 (96%) |  |
| Yes | 6,077 (27%) | 25 (4.3%) |  |
| **Did the patient express to family or others the intent to be a donor** |  |  | <0.001 |
| Missing | 14 (<0.1%) | 0 (0%) |  |
| Negative / No | 6,742 (30%) | 142 (24%) |  |
| Positive / Yes | 4,969 (22%) | 181 (31%) |  |
| Unknown | 10,968 (48%) | 264 (45%) |  |
| **Donor/s Gender** |  |  | <0.001 |
| Female | 9,127 (40%) | 191 (33%) |  |
| Male | 13,566 (60%) | 396 (67%) |  |
| **Hepatitis B Core Antibody Status** |  |  | <0.001 |
| Indeterminate | 8 (<0.1%) | 0 (0%) |  |
| Negative | 16,934 (75%) | 549 (94%) |  |
| Not Done | 3,970 (17%) | 4 (0.7%) |  |
| Pending | 411 (1.8%) | 18 (3.1%) |  |
| Positive | 1,080 (4.8%) | 15 (2.6%) |  |
| Unknown | 253 (1.1%) | 1 (0.2%) |  |
| Missing | 37 (0.2%) | 0 (0%) |  |
| **Donor RNA Nat serology value for hbv test** |  |  | >0.9 |
| Missing | 22,686 (100%) | 587 (100%) |  |
| Negative | 4 (<0.1%) | 0 (0%) |  |
| Not Done | 2 (<0.1%) | 0 (0%) |  |
| Unknown | 1 (<0.1%) | 0 (0%) |  |
| **HBsAb (Hepatitis B Surface Antibody)** |  |  | <0.001 |
| Missing | 5,567 (25%) | 0 (0%) |  |
| Cannot Disclose | 4 (<0.1%) | 0 (0%) |  |
| Indeterminate | 33 (0.1%) | 2 (0.3%) |  |
| Negative | 3,539 (16%) | 90 (15%) |  |
| Not Done | 12,081 (53%) | 448 (76%) |  |
| Positive | 1,426 (6.3%) | 46 (7.8%) |  |
| Unknown | 43 (0.2%) | 1 (0.2%) |  |
| **HBV//Surface Antigen:** |  |  | 0.7 |
| Missing | 1 (<0.1%) | 0 (0%) |  |
| Negative | 22,617 (100%) | 587 (100%) |  |
| Not Done | 31 (0.1%) | 0 (0%) |  |
| Positive | 32 (0.1%) | 0 (0%) |  |
| Unknown | 12 (<0.1%) | 0 (0%) |  |
| **Donor RNA Nat serology value for hcv test** |  |  | >0.9 |
| Missing | 22,686 (100%) | 587 (100%) |  |
| Negative | 3 (<0.1%) | 0 (0%) |  |
| Not Done | 3 (<0.1%) | 0 (0%) |  |
| Unknown | 1 (<0.1%) | 0 (0%) |  |
| **HCV Antibody Status** |  |  | <0.001 |
| Indeterminate | 10 (<0.1%) | 0 (0%) |  |
| Negative | 17,430 (77%) | 556 (95%) |  |
| Not Done | 3,898 (17%) | 3 (0.5%) |  |
| Pending | 388 (1.7%) | 19 (3.2%) |  |
| Positive | 696 (3.1%) | 8 (1.4%) |  |
| Unknown | 235 (1.0%) | 1 (0.2%) |  |
| Missing | 36 (0.2%) | 0 (0%) |  |
| **Heavy Alcohol Use (heavy= 2+ drinks/day):** |  |  | <0.001 |
| Missing | 1,024 (4.5%) | 0 (0%) |  |
| Negative / No | 18,266 (80%) | 511 (87%) |  |
| Positive / Yes | 3,105 (14%) | 71 (12%) |  |
| Unknown | 298 (1.3%) | 5 (0.9%) |  |
| **Heparin** |  |  | 0.046 |
| Missing | 1 (<0.1%) | 0 (0%) |  |
| Negative / No | 1,509 (6.6%) | 47 (8.0%) |  |
| Positive / Yes | 21,170 (93%) | 538 (92%) |  |
| Unknown | 13 (<0.1%) | 2 (0.3%) |  |
| **Donor serum creatinine > 1.5 (1= yes, 0= no)** |  |  | <0.001 |
| No | 16,854 (74%) | 504 (86%) |  |
| Yes | 5,836 (26%) | 83 (14%) |  |
| Missing | 3 (<0.1%) | 0 (0%) |  |
| **Alcohol Dependency** |  |  | <0.001 |
| Missing | 21,501 (95%) | 587 (100%) |  |
| Negative / No | 955 (4.2%) | 0 (0%) |  |
| Positive / Yes | 229 (1.0%) | 0 (0%) |  |
| Unknown | 8 (<0.1%) | 0 (0%) |  |
| **History of Cancer** |  |  | 0.8 |
| NO | 21,780 (96%) | 576 (98%) |  |
| CNS TUMOR - OTHER | 39 (0.2%) | 2 (0.3%) |  |
| GENITOURINARY - BLADDER | 10 (<0.1%) | 0 (0%) |  |
| GENITOURINARY - UTERINE CERVIX | 75 (0.3%) | 2 (0.3%) |  |
| GENITOURINARY - UTERINE BODY ENDOMETRIAL | 10 (<0.1%) | 0 (0%) |  |
| GENITOURINARY - OVARIAN | 19 (<0.1%) | 0 (0%) |  |
| GENITOURINARY - PENIS, TESTICULAR | 11 (<0.1%) | 1 (0.2%) |  |
| SKIN - SQUAMOUS, BASAL CELL | 285 (1.3%) | 5 (0.9%) |  |
| GENITOURINARY - PROSTATE | 33 (0.1%) | 0 (0%) |  |
| GENITOURINARY - KIDNEY | 6 (<0.1%) | 0 (0%) |  |
| GENITOURINARY - UNKNOWN | 3 (<0.1%) | 0 (0%) |  |
| GASTROINTESTINAL - STOMACH | 1 (<0.1%) | 0 (0%) |  |
| GASTROINTESTINAL - SMALL INTESTINE | 1 (<0.1%) | 0 (0%) |  |
| GASTROINTESTINAL - COLO-RECTAL | 13 (<0.1%) | 0 (0%) |  |
| BREAST | 54 (0.2%) | 0 (0%) |  |
| SKIN - MELANOMA | 29 (0.1%) | 0 (0%) |  |
| THYROID | 14 (<0.1%) | 0 (0%) |  |
| TONGUE/THROAT | 4 (<0.1%) | 0 (0%) |  |
| LARYNX | 2 (<0.1%) | 0 (0%) |  |
| LUNG (include bronchial) | 6 (<0.1%) | 0 (0%) |  |
| LEUKEMIA/LYMPHOMA | 7 (<0.1%) | 0 (0%) |  |
| CNS TUMOR - ASTROCYTOMA | 17 (<0.1%) | 0 (0%) |  |
| CNS TUMOR - GLIOBLASTOMA MULTIFORME | 33 (0.1%) | 0 (0%) |  |
| CNS TUMOR - MEDULLOBLASTOMA | 1 (<0.1%) | 0 (0%) |  |
| CNS TUMOR - MENINGIOMA | 19 (<0.1%) | 0 (0%) |  |
| UNKNOWN | 99 (0.4%) | 0 (0%) |  |
| OTHER, SPECIFY | 121 (0.5%) | 1 (0.2%) |  |
| Missing | 1 (<0.1%) | 0 (0%) |  |
| **Cigarette Use > 20 Pack Years - Ever** |  |  | 0.032 |
| Missing | 1 (<0.1%) | 0 (0%) |  |
| Negative / No | 15,904 (70%) | 476 (81%) |  |
| Positive / Yes | 6,516 (29%) | 107 (18%) |  |
| Unknown | 272 (1.2%) | 4 (0.7%) |  |
| **Cocaine Use** |  |  | 0.071 |
| Missing | 1 (<0.1%) | 0 (0%) |  |
| Negative / No | 19,350 (85%) | 479 (82%) |  |
| Positive / Yes | 2,962 (13%) | 96 (16%) |  |
| Unknown | 380 (1.7%) | 12 (2.0%) |  |
| **History of Diabetes** |  |  | 0.003 |
| No | 20,158 (89%) | 555 (95%) |  |
| Unknown | 124 (0.5%) | 0 (0%) |  |
| Yes, >10 years | 836 (3.7%) | 10 (1.7%) |  |
| Yes, 0-5 years | 896 (3.9%) | 15 (2.6%) |  |
| Yes, 6-10 years | 429 (1.9%) | 4 (0.7%) |  |
| Yes, Duration Unknown | 249 (1.1%) | 3 (0.5%) |  |
| Missing | 1 (<0.1%) | 0 (0%) |  |
| **History of Hypertension** |  |  | <0.001 |
| No | 14,745 (65%) | 457 (78%) |  |
| Unknown | 146 (0.6%) | 1 (0.2%) |  |
| Yes, >10 years | 1,932 (8.5%) | 20 (3.4%) |  |
| Yes, 0-5 years | 3,421 (15%) | 69 (12%) |  |
| Yes, 6-10 years | 1,393 (6.1%) | 21 (3.6%) |  |
| Yes, Duration Unknown | 1,055 (4.6%) | 19 (3.2%) |  |
| Missing | 1 (<0.1%) | 0 (0%) |  |
| **Insulin Dependent** |  |  | 0.6 |
| No | 1,265 (5.6%) | 14 (2.4%) |  |
| Unknown | 114 (0.5%) | 2 (0.3%) |  |
| Yes, >10 years | 419 (1.8%) | 7 (1.2%) |  |
| Yes, 0-5 years | 342 (1.5%) | 7 (1.2%) |  |
| Yes, 6-10 years | 163 (0.7%) | 2 (0.3%) |  |
| Yes, Duration Unknown | 106 (0.5%) | 0 (0%) |  |
| Missing | 20,284 (89%) | 555 (95%) |  |
| **IV Drug Use - Pre-6/30/2004** |  |  | 0.001 |
| Missing | 21,502 (95%) | 587 (100%) |  |
| Negative / No | 1,144 (5.0%) | 0 (0%) |  |
| Positive / Yes | 36 (0.2%) | 0 (0%) |  |
| Unknown | 11 (<0.1%) | 0 (0%) |  |
| **Other Drug Abuse** |  |  | 0.033 |
| Missing | 1 (<0.1%) | 0 (0%) |  |
| Negative / No | 15,511 (68%) | 347 (59%) |  |
| Positive / Yes | 6,877 (30%) | 235 (40%) |  |
| Unknown | 304 (1.3%) | 5 (0.9%) |  |
| **History of Previous MI** |  |  | <0.001 |
| Missing | 612 (2.7%) | 0 (0%) |  |
| Negative / No | 20,791 (92%) | 565 (96%) |  |
| Positive / Yes | 910 (4.0%) | 17 (2.9%) |  |
| Unknown | 380 (1.7%) | 5 (0.9%) |  |
| **Donor RNA Nat serology value for hiv test** |  |  | >0.9 |
| Missing | 22,686 (100%) | 587 (100%) |  |
| Negative | 5 (<0.1%) | 0 (0%) |  |
| Not Done | 1 (<0.1%) | 0 (0%) |  |
| Unknown | 1 (<0.1%) | 0 (0%) |  |
| **Donor/s Home State** |  |  | <0.001 |
| ALASKA | 46 (0.2%) | 1 (0.2%) |  |
| ALABAMA | 421 (1.9%) | 4 (0.7%) |  |
| ARKANSAS | 314 (1.4%) | 7 (1.2%) |  |
| PAGO PAGO | 3 (<0.1%) | 0 (0%) |  |
| ARIZONA | 324 (1.4%) | 7 (1.2%) |  |
| CALIFORNIA | 2,051 (9.0%) | 29 (4.9%) |  |
| COLORADO | 245 (1.1%) | 8 (1.4%) |  |
| CONNECTICUT | 198 (0.9%) | 4 (0.7%) |  |
| DISTRICT OF COLUMBIA | 52 (0.2%) | 1 (0.2%) |  |
| DELAWARE | 90 (0.4%) | 3 (0.5%) |  |
| FLORIDA | 1,744 (7.7%) | 74 (13%) |  |
| GEORGIA | 860 (3.8%) | 8 (1.4%) |  |
| HAWAII | 93 (0.4%) | 1 (0.2%) |  |
| IOWA | 190 (0.8%) | 13 (2.2%) |  |
| IDAHO | 79 (0.3%) | 2 (0.3%) |  |
| ILLINOIS | 847 (3.7%) | 28 (4.8%) |  |
| INDIANA | 605 (2.7%) | 14 (2.4%) |  |
| KANSAS | 293 (1.3%) | 7 (1.2%) |  |
| KENTUCKY | 327 (1.4%) | 9 (1.5%) |  |
| LOUISIANA | 539 (2.4%) | 25 (4.3%) |  |
| MASSACHUSETTS | 239 (1.1%) | 17 (2.9%) |  |
| MARYLAND | 451 (2.0%) | 14 (2.4%) |  |
| MAINE | 74 (0.3%) | 4 (0.7%) |  |
| MICHIGAN | 722 (3.2%) | 25 (4.3%) |  |
| MINNESOTA | 308 (1.4%) | 20 (3.4%) |  |
| MISSOURI | 645 (2.8%) | 22 (3.7%) |  |
| MISSISSIPPI | 310 (1.4%) | 4 (0.7%) |  |
| MONTANA | 47 (0.2%) | 1 (0.2%) |  |
| FOREIGN COUNTRY | 22 (<0.1%) | 1 (0.2%) |  |
| NORTH CAROLINA | 718 (3.2%) | 12 (2.0%) |  |
| NORTH DAKOTA | 41 (0.2%) | 2 (0.3%) |  |
| NEBRASKA | 124 (0.5%) | 6 (1.0%) |  |
| NEW HAMPSHIRE | 58 (0.3%) | 3 (0.5%) |  |
| NEW JERSEY | 573 (2.5%) | 5 (0.9%) |  |
| NEW MEXICO | 173 (0.8%) | 1 (0.2%) |  |
| NEVADA | 180 (0.8%) | 2 (0.3%) |  |
| NEW YORK | 985 (4.3%) | 16 (2.7%) |  |
| OHIO | 827 (3.6%) | 24 (4.1%) |  |
| OKLAHOMA | 287 (1.3%) | 2 (0.3%) |  |
| OREGON | 175 (0.8%) | 5 (0.9%) |  |
| PENNSYLVANIA | 1,045 (4.6%) | 26 (4.4%) |  |
| PUERTO RICO | 382 (1.7%) | 0 (0%) |  |
| RHODE ISLAND | 39 (0.2%) | 3 (0.5%) |  |
| SOUTH CAROLINA | 625 (2.8%) | 2 (0.3%) |  |
| SOUTH DAKOTA | 55 (0.2%) | 0 (0%) |  |
| TENNESSEE | 634 (2.8%) | 14 (2.4%) |  |
| TEXAS | 1,765 (7.8%) | 15 (2.6%) |  |
| UTAH | 172 (0.8%) | 14 (2.4%) |  |
| VIRGINIA | 483 (2.1%) | 22 (3.7%) |  |
| VIRGIN ISLANDS | 5 (<0.1%) | 0 (0%) |  |
| VERMONT | 22 (<0.1%) | 2 (0.3%) |  |
| WASHINGTON | 274 (1.2%) | 12 (2.0%) |  |
| WISCONSIN | 557 (2.5%) | 37 (6.3%) |  |
| WEST VIRGINIA | 172 (0.8%) | 7 (1.2%) |  |
| WYOMING | 43 (0.2%) | 2 (0.3%) |  |
| UNKNOWN | 140 (0.6%) | 0 (0%) |  |
| **Donor RNA Nat serology value for htlv test** |  |  | >0.9 |
| Missing | 22,686 (100%) | 587 (100%) |  |
| Not Done | 6 (<0.1%) | 0 (0%) |  |
| Unknown | 1 (<0.1%) | 0 (0%) |  |
| **Donor history of hypertension (1= yes, 0= no)** |  |  | <0.001 |
| No | 14,509 (64%) | 456 (78%) |  |
| Yes | 8,053 (35%) | 130 (22%) |  |
| Missing | 131 (0.6%) | 1 (0.2%) |  |
| **Infection Source: Blood** |  |  | 0.4 |
| No | 20,971 (92%) | 537 (91%) |  |
| Yes | 1,722 (7.6%) | 50 (8.5%) |  |
| **Infection Source: Blood/Confirmed by Culture** |  |  | 0.004 |
| Missing | 20,951 (92%) | 536 (91%) |  |
| No | 355 (1.6%) | 1 (0.2%) |  |
| Yes | 1,387 (6.1%) | 50 (8.5%) |  |
| **Infection Source: Lung** |  |  | 0.028 |
| No | 15,108 (67%) | 416 (71%) |  |
| Yes | 7,585 (33%) | 171 (29%) |  |
| **Infection Source: Lung/Confirmed by Culture** |  |  | 0.021 |
| Missing | 14,993 (66%) | 408 (70%) |  |
| No | 149 (0.7%) | 8 (1.4%) |  |
| Yes | 7,551 (33%) | 171 (29%) |  |
| **Infection Source: Other** |  |  | 0.4 |
| No | 21,284 (94%) | 556 (95%) |  |
| Yes | 1,409 (6.2%) | 31 (5.3%) |  |
| **Infection Source: Other/Confimed by Culture** |  |  | 0.4 |
| Missing | 21,268 (94%) | 556 (95%) |  |
| No | 31 (0.1%) | 0 (0%) |  |
| Yes | 1,394 (6.1%) | 31 (5.3%) |  |
| **Infection Source: Urine** |  |  | 0.7 |
| No | 20,554 (91%) | 535 (91%) |  |
| Yes | 2,139 (9.4%) | 52 (8.9%) |  |
| **Infection Source: Urine/Confirmed by Culture** |  |  | 0.078 |
| Missing | 20,534 (90%) | 534 (91%) |  |
| No | 322 (1.4%) | 2 (0.3%) |  |
| Yes | 1,837 (8.1%) | 51 (8.7%) |  |
| **Three or More Inotropic Agents at Time of Incision** |  |  | 0.057 |
| Missing | 2 (<0.1%) | 0 (0%) |  |
| No | 22,049 (97%) | 586 (100%) |  |
| Yes | 642 (2.8%) | 1 (0.2%) |  |
| **Inotropic Support** |  |  | <0.001 |
| Missing | 2 (<0.1%) | 0 (0%) |  |
| Negative / No | 9,258 (41%) | 566 (96%) |  |
| Positive / Yes | 13,361 (59%) | 20 (3.4%) |  |
| Unknown | 72 (0.3%) | 1 (0.2%) |  |
| **Insulin:** |  |  | <0.001 |
| Missing | 1,019 (4.5%) | 0 (0%) |  |
| Negative / No | 8,003 (35%) | 398 (68%) |  |
| Positive / Yes | 13,634 (60%) | 185 (32%) |  |
| Unknown | 37 (0.2%) | 4 (0.7%) |  |
| **Left Lung Machine Perfusion Intended or Performed:** |  |  | >0.9 |
| Missing | 22,692 (100%) | 587 (100%) |  |
| No | 1 (<0.1%) | 0 (0%) |  |
| **Was the patient declared legally brain dead:** |  |  | 0.038 |
| Missing | 21,241 (94%) | 565 (96%) |  |
| No | 1 (<0.1%) | 0 (0%) |  |
| Yes | 1,451 (6.4%) | 22 (3.7%) |  |
| **Kidney Biopsy (left)** |  |  | 0.001 |
| Missing | 1,135 (5.0%) | 8 (1.4%) |  |
| No | 13,170 (58%) | 320 (55%) |  |
| Yes | 8,388 (37%) | 259 (44%) |  |
| **Pump (Left Kidney)** |  |  | <0.001 |
| Missing | 2,504 (11%) | 27 (4.6%) |  |
| No | 15,496 (68%) | 223 (38%) |  |
| Yes | 4,693 (21%) | 337 (57%) |  |
| **Transferred on pump (left)://Transferred on pump:** |  |  | <0.001 |
| Missing | 18,161 (80%) | 250 (43%) |  |
| No | 1,697 (7.5%) | 112 (19%) |  |
| Yes | 2,835 (12%) | 225 (38%) |  |
| **Left Lung Bronchoscopy:** |  |  | <0.001 |
| No Bronchoscopy | 986 (4.3%) | 46 (7.8%) |  |
| Bronchoscopy Results normal | 4,012 (18%) | 20 (3.4%) |  |
| Bronchoscopy Results, Abnormal-purulent secretions | 673 (3.0%) | 2 (0.3%) |  |
| Bronchoscopy Results, Abnormal-aspiration of foreign body | 52 (0.2%) | 0 (0%) |  |
| Bronchoscopy Results, Abnormal-blood | 266 (1.2%) | 1 (0.2%) |  |
| Bronchoscopy Results, Abnormal-anatomy/other lesion | 72 (0.3%) | 1 (0.2%) |  |
| Bronchoscopy Results, Unknown | 99 (0.4%) | 0 (0%) |  |
| Unknown if bronchoscopy performed | 29 (0.1%) | 0 (0%) |  |
| Missing | 16,504 (73%) | 517 (88%) |  |
| **Liver Biopsy** |  |  | <0.001 |
| Missing | 51 (0.2%) | 0 (0%) |  |
| No | 14,810 (65%) | 440 (75%) |  |
| Yes | 7,832 (35%) | 147 (25%) |  |
| **Granulomas Histology performed** |  |  | >0.9 |
| No | 21,356 (94%) | 584 (99%) |  |
| Yes | 53 (0.2%) | 1 (0.2%) |  |
| Missing | 1,284 (5.7%) | 2 (0.3%) |  |
| **Hemosidera Histology performed** |  |  | 0.7 |
| No | 21,353 (94%) | 584 (99%) |  |
| Yes | 56 (0.2%) | 1 (0.2%) |  |
| Missing | 1,284 (5.7%) | 2 (0.3%) |  |
| **Other Histology performed** |  |  | 0.015 |
| No | 19,245 (85%) | 544 (93%) |  |
| Yes | 2,164 (9.5%) | 41 (7.0%) |  |
| Missing | 1,284 (5.7%) | 2 (0.3%) |  |
| **Medical Examiner/Coroner** |  |  | 0.002 |
| NO | 8,797 (39%) | 176 (30%) |  |
| YES, MEDICAL EXAMINER CONSENTED | 13,799 (61%) | 409 (70%) |  |
| YES, MEDICAL EXAMINER REFUSED CONSENT | 44 (0.2%) | 2 (0.3%) |  |
| UNKNOWN | 52 (0.2%) | 0 (0%) |  |
| Missing | 1 (<0.1%) | 0 (0%) |  |
| **Does the Donor meet CDC guidelines for High Risk for an organ donor:** |  |  | <0.001 |
| Missing | 1,028 (4.5%) | 0 (0%) |  |
| Negative / No | 19,577 (86%) | 526 (90%) |  |
| Positive / Yes | 2,005 (8.8%) | 60 (10%) |  |
| Unknown | 83 (0.4%) | 1 (0.2%) |  |
| **Non-Heart Beating Donor** |  |  | <0.001 |
| Missing | 0 (0%) | 0 (0%) |  |
| No | 22,164 (98%) | 0 (0%) |  |
| Yes | 529 (2.3%) | 587 (100%) |  |
| **Controlled** |  |  | <0.001 |
| Missing | 22,163 (98%) | 0 (0%) |  |
| Negative / No | 18 (<0.1%) | 0 (0%) |  |
| Positive / Yes | 507 (2.2%) | 587 (100%) |  |
| Unknown | 5 (<0.1%) | 0 (0%) |  |
| **Core Cooling Used** |  |  | <0.001 |
| Missing | 22,163 (98%) | 0 (0%) |  |
| No | 298 (1.3%) | 352 (60%) |  |
| Yes | 232 (1.0%) | 235 (40%) |  |
| **Organ was used in a shared 1 (vs local 0) TX** |  |  | 0.2 |
| No | 16,242 (72%) | 406 (69%) |  |
| Yes | 6,451 (28%) | 181 (31%) |  |
| **Lung - Was pO2 done:** |  |  | <0.001 |
| Missing | 859 (3.8%) | 0 (0%) |  |
| Negative / No | 598 (2.6%) | 21 (3.6%) |  |
| Positive / Yes | 21,176 (93%) | 565 (96%) |  |
| Unknown | 60 (0.3%) | 1 (0.2%) |  |
| **Pre-Recov Meds given Donor: Diuretics** |  |  | <0.001 |
| Missing | 1 (<0.1%) | 0 (0%) |  |
| Negative / No | 9,270 (41%) | 452 (77%) |  |
| Positive / Yes | 13,382 (59%) | 131 (22%) |  |
| Unknown | 40 (0.2%) | 4 (0.7%) |  |
| **Pre-Recov Meds given Donor: Steroids** |  |  | <0.001 |
| Missing | 1 (<0.1%) | 0 (0%) |  |
| Negative / No | 5,130 (23%) | 476 (81%) |  |
| Positive / Yes | 17,532 (77%) | 108 (18%) |  |
| Unknown | 30 (0.1%) | 3 (0.5%) |  |
| **Pre-Recov Meds given Donor: T3** |  |  | 0.057 |
| Missing | 1 (<0.1%) | 0 (0%) |  |
| Negative / No | 22,455 (99%) | 585 (100%) |  |
| Positive / Yes | 208 (0.9%) | 0 (0%) |  |
| Unknown | 29 (0.1%) | 2 (0.3%) |  |
| **Pre-Recov Meds given Donor: T4** |  |  | <0.001 |
| Missing | 1 (<0.1%) | 0 (0%) |  |
| Negative / No | 9,498 (42%) | 561 (96%) |  |
| Positive / Yes | 13,177 (58%) | 24 (4.1%) |  |
| Unknown | 17 (<0.1%) | 2 (0.3%) |  |
| **Previous Gastrointestinal Disease** |  |  | <0.001 |
| Missing | 699 (3.1%) | 2 (0.3%) |  |
| Negative / No | 18,583 (82%) | 544 (93%) |  |
| Positive / Yes | 1,458 (6.4%) | 37 (6.3%) |  |
| Unknown | 1,953 (8.6%) | 4 (0.7%) |  |
| **Protein in Urine** |  |  | <0.001 |
| Missing | 1 (<0.1%) | 0 (0%) |  |
| Negative / No | 13,349 (59%) | 264 (45%) |  |
| Positive / Yes | 8,880 (39%) | 306 (52%) |  |
| Unknown | 463 (2.0%) | 17 (2.9%) |  |
| **Was a pulmonary artery catheter placed:** |  |  | <0.001 |
| Missing | 609 (2.7%) | 0 (0%) |  |
| No | 19,132 (84%) | 578 (98%) |  |
| Yes | 2,952 (13%) | 9 (1.5%) |  |
| **SRTR Donor Race** |  |  | 0.002 |
| Asian | 473 (2.1%) | 7 (1.2%) |  |
| Black | 3,996 (18%) | 55 (9.4%) |  |
| Multiracial | 63 (0.3%) | 2 (0.3%) |  |
| Native American | 85 (0.4%) | 1 (0.2%) |  |
| Pacific Islander | 44 (0.2%) | 0 (0%) |  |
| White | 18,032 (79%) | 522 (89%) |  |
| **Recovered Outside the U.S.** |  |  | 0.003 |
| N | 22,335 (98%) | 587 (100%) |  |
| Y | 358 (1.6%) | 0 (0%) |  |
| **Duration of Resuscitation** |  |  | 0.15 |
| 1 | 33 (0.1%) | 0 (0%) |  |
| 10 | 116 (0.5%) | 2 (0.3%) |  |
| 11 | 13 (<0.1%) | 0 (0%) |  |
| 12 | 25 (0.1%) | 0 (0%) |  |
| 120 | 5 (<0.1%) | 0 (0%) |  |
| 13 | 16 (<0.1%) | 0 (0%) |  |
| 14 | 17 (<0.1%) | 0 (0%) |  |
| 15 | 100 (0.4%) | 2 (0.3%) |  |
| 16 | 19 (<0.1%) | 0 (0%) |  |
| 17 | 9 (<0.1%) | 0 (0%) |  |
| 18 | 15 (<0.1%) | 0 (0%) |  |
| 19 | 6 (<0.1%) | 0 (0%) |  |
| 2 | 50 (0.2%) | 0 (0%) |  |
| 20 | 112 (0.5%) | 0 (0%) |  |
| 21 | 5 (<0.1%) | 0 (0%) |  |
| 22 | 13 (<0.1%) | 0 (0%) |  |
| 23 | 11 (<0.1%) | 0 (0%) |  |
| 24 | 10 (<0.1%) | 1 (0.2%) |  |
| 25 | 46 (0.2%) | 1 (0.2%) |  |
| 26 | 2 (<0.1%) | 0 (0%) |  |
| 27 | 5 (<0.1%) | 0 (0%) |  |
| 28 | 9 (<0.1%) | 0 (0%) |  |
| 29 | 3 (<0.1%) | 0 (0%) |  |
| 3 | 45 (0.2%) | 0 (0%) |  |
| 30 | 104 (0.5%) | 2 (0.3%) |  |
| 31 | 5 (<0.1%) | 0 (0%) |  |
| 32 | 6 (<0.1%) | 1 (0.2%) |  |
| 33 | 13 (<0.1%) | 0 (0%) |  |
| 34 | 4 (<0.1%) | 0 (0%) |  |
| 35 | 30 (0.1%) | 1 (0.2%) |  |
| 36 | 5 (<0.1%) | 0 (0%) |  |
| 37 | 3 (<0.1%) | 0 (0%) |  |
| 38 | 5 (<0.1%) | 0 (0%) |  |
| 39 | 6 (<0.1%) | 0 (0%) |  |
| 4 | 30 (0.1%) | 1 (0.2%) |  |
| 40 | 51 (0.2%) | 1 (0.2%) |  |
| 41 | 3 (<0.1%) | 0 (0%) |  |
| 42 | 2 (<0.1%) | 0 (0%) |  |
| 43 | 2 (<0.1%) | 0 (0%) |  |
| 44 | 3 (<0.1%) | 0 (0%) |  |
| 45 | 42 (0.2%) | 0 (0%) |  |
| 46 | 6 (<0.1%) | 0 (0%) |  |
| 47 | 3 (<0.1%) | 0 (0%) |  |
| 48 | 6 (<0.1%) | 0 (0%) |  |
| 49 | 1 (<0.1%) | 0 (0%) |  |
| 5 | 83 (0.4%) | 1 (0.2%) |  |
| 50 | 13 (<0.1%) | 0 (0%) |  |
| 51 | 4 (<0.1%) | 0 (0%) |  |
| 52 | 1 (<0.1%) | 0 (0%) |  |
| 53 | 2 (<0.1%) | 0 (0%) |  |
| 54 | 3 (<0.1%) | 0 (0%) |  |
| 55 | 5 (<0.1%) | 1 (0.2%) |  |
| 56 | 2 (<0.1%) | 0 (0%) |  |
| 58 | 0 (0%) | 1 (0.2%) |  |
| 59 | 2 (<0.1%) | 0 (0%) |  |
| 6 | 25 (0.1%) | 1 (0.2%) |  |
| 60 | 30 (0.1%) | 0 (0%) |  |
| 61 | 1 (<0.1%) | 0 (0%) |  |
| 62 | 2 (<0.1%) | 0 (0%) |  |
| 63 | 1 (<0.1%) | 0 (0%) |  |
| 65 | 1 (<0.1%) | 0 (0%) |  |
| 69 | 2 (<0.1%) | 0 (0%) |  |
| 7 | 20 (<0.1%) | 0 (0%) |  |
| 70 | 4 (<0.1%) | 0 (0%) |  |
| 72 | 1 (<0.1%) | 0 (0%) |  |
| 75 | 3 (<0.1%) | 0 (0%) |  |
| 8 | 26 (0.1%) | 0 (0%) |  |
| 80 | 4 (<0.1%) | 0 (0%) |  |
| 81 | 1 (<0.1%) | 0 (0%) |  |
| 82 | 1 (<0.1%) | 0 (0%) |  |
| 88 | 1 (<0.1%) | 0 (0%) |  |
| 9 | 14 (<0.1%) | 0 (0%) |  |
| 90 | 7 (<0.1%) | 0 (0%) |  |
| 95 | 1 (<0.1%) | 0 (0%) |  |
| 97 | 1 (<0.1%) | 0 (0%) |  |
| 98 | 1 (<0.1%) | 0 (0%) |  |
| 99 | 3 (<0.1%) | 0 (0%) |  |
| Missing | 21,413 (94%) | 571 (97%) |  |
| **Right Lung Machine Perfusion Intended or Performed** |  |  | >0.9 |
| Missing | 22,692 (100%) | 587 (100%) |  |
| No | 1 (<0.1%) | 0 (0%) |  |
| **RPR-VDRL** |  |  | 0.4 |
| Missing | 1 (<0.1%) | 0 (0%) |  |
| Indeterminate | 3 (<0.1%) | 0 (0%) |  |
| Negative | 22,382 (99%) | 583 (99%) |  |
| Not Done | 126 (0.6%) | 3 (0.5%) |  |
| Positive | 169 (0.7%) | 1 (0.2%) |  |
| Unknown | 12 (<0.1%) | 0 (0%) |  |
| **Kidney Biopsy (right)** |  |  | <0.001 |
| Missing | 1,143 (5.0%) | 8 (1.4%) |  |
| No | 13,080 (58%) | 315 (54%) |  |
| Yes | 8,470 (37%) | 264 (45%) |  |
| **Pump (Right Kidney)** |  |  | <0.001 |
| Missing | 2,495 (11%) | 26 (4.4%) |  |
| No | 15,557 (69%) | 220 (37%) |  |
| Yes | 4,641 (20%) | 341 (58%) |  |
| **Transferred on pump (right)://Transferred on pump:** |  |  | <0.001 |
| Missing | 18,211 (80%) | 246 (42%) |  |
| No | 1,738 (7.7%) | 111 (19%) |  |
| Yes | 2,744 (12%) | 230 (39%) |  |
| **Right Lung Bronchoscopy:** |  |  | <0.001 |
| No Bronchoscopy | 1,042 (4.6%) | 47 (8.0%) |  |
| Bronchoscopy Results normal | 3,776 (17%) | 17 (2.9%) |  |
| Bronchoscopy Results, Abnormal-purulent secretions | 771 (3.4%) | 3 (0.5%) |  |
| Bronchoscopy Results, Abnormal-aspiration of foreign body | 72 (0.3%) | 0 (0%) |  |
| Bronchoscopy Results, Abnormal-blood | 275 (1.2%) | 1 (0.2%) |  |
| Bronchoscopy Results, Abnormal-anatomy/other lesion | 79 (0.3%) | 1 (0.2%) |  |
| Bronchoscopy Results, Unknown | 105 (0.5%) | 0 (0%) |  |
| Unknown if bronchoscopy performed | 31 (0.1%) | 0 (0%) |  |
| Missing | 16,542 (73%) | 518 (88%) |  |
| **strongyloides test result selection** |  |  | >0.9 |
| Missing | 22,692 (100%) | 587 (100%) |  |
| Not Done | 1 (<0.1%) | 0 (0%) |  |
| **Lifestyle Factors: Tattoos** |  |  | 0.029 |
| Missing | 1 (<0.1%) | 0 (0%) |  |
| Negative / No | 16,581 (73%) | 388 (66%) |  |
| Positive / Yes | 6,032 (27%) | 197 (34%) |  |
| Unknown | 79 (0.3%) | 2 (0.3%) |  |
| **Is there time for crossmatch?** |  |  | <0.001 |
| Missing | 9,920 (44%) | 463 (79%) |  |
| No | 574 (2.5%) | 1 (0.2%) |  |
| Yes | 12,199 (54%) | 123 (21%) |  |
| **Number of transfusions during this (terminal) hospitalization:** |  |  | 0.056 |
| NONE | 11,530 (51%) | 349 (59%) |  |
| 1 - 5 | 6,919 (30%) | 167 (28%) |  |
| 6 - 10 | 1,862 (8.2%) | 41 (7.0%) |  |
| GREATER THAN 10 | 1,334 (5.9%) | 30 (5.1%) |  |
| UNKNOWN | 25 (0.1%) | 0 (0%) |  |
| Missing | 1,023 (4.5%) | 0 (0%) |  |
| **Donor/s HLA - Typing Method Class I** |  |  | <0.001 |
| Serology | 5,024 (22%) | 89 (15%) |  |
| DNA | 11,456 (50%) | 355 (60%) |  |
| 3 | 5,408 (24%) | 137 (23%) |  |
| Missing | 805 (3.5%) | 6 (1.0%) |  |
| **Donor/s HLA - Typing Method Class II** |  |  | 0.010 |
| Serology | 3,697 (16%) | 74 (13%) |  |
| DNA | 13,467 (59%) | 388 (66%) |  |
| 3 | 4,724 (21%) | 119 (20%) |  |
| Missing | 805 (3.5%) | 6 (1.0%) |  |
| **Use Double KI allocation, not Expanded Donor** |  |  | 0.003 |
| Missing | 22,001 (97%) | 583 (99%) |  |
| No | 298 (1.3%) | 2 (0.3%) |  |
| Yes | 394 (1.7%) | 2 (0.3%) |  |
| **Vasodilators** |  |  | <0.001 |
| Missing | 2 (<0.1%) | 0 (0%) |  |
| Negative / No | 19,307 (85%) | 534 (91%) |  |
| Positive / Yes | 3,349 (15%) | 48 (8.2%) |  |
| Unknown | 35 (0.2%) | 5 (0.9%) |  |
| **Wall Abnormalities //Global:** |  |  | <0.001 |
| Missing | 20,007 (88%) | 572 (97%) |  |
| No | 1,587 (7.0%) | 8 (1.4%) |  |
| Yes | 1,099 (4.8%) | 7 (1.2%) |  |
| **Wall Abnormalities //Segmental:** |  |  | <0.001 |
| Missing | 20,014 (88%) | 572 (97%) |  |
| No | 1,680 (7.4%) | 10 (1.7%) |  |
| Yes | 999 (4.4%) | 5 (0.9%) |  |
| **Donor RNA Nat serology value for West Nile test** |  |  | >0.9 |
| Missing | 22,686 (100%) | 587 (100%) |  |
| Negative | 2 (<0.1%) | 0 (0%) |  |
| Not Done | 4 (<0.1%) | 0 (0%) |  |
| Unknown | 1 (<0.1%) | 0 (0%) |  |
| **^1^Median (Q1, Q3); n (%)** | | | |
| **^2^Wilcoxon rank sum test; Pearson's Chi-squared test with simulated p-value (based on 2000 replicates)** | | | |

# **Table D3. Donor Characteristics by Donor Type, 2013-01-01 to 2018-12-31**

| **Characteristic** | **DBD**  N = 17,373^1^ | **DCD**  N = 1,174^1^ | **p-value**^2^ |
| --- | --- | --- | --- |
| **Calculated Donor Age in Years at Organ Recovery or Referral Date** | 40.00 (27.00, 54.00) | 34.00 (24.00, 46.00) | <0.001 |
| **Donor/s Age in Months** | 491.00 (335.00, 651.00) | 410.50 (295.00, 560.00) | <0.001 |
| **most recent serum creatinine** | 1.10 (0.80, 1.70) | 0.80 (0.60, 1.09) | <0.001 |
| **BUN** | 19.00 (12.00, 30.00) | 16.00 (11.00, 23.00) | <0.001 |
| **Cancer Free Interval:** | 7.00 (2.00, 15.00) | 7.00 (3.00, 20.00) | 0.8 |
| **Cardiac Index (final) //Cardiac Index:** | 4.00 (3.30, 4.80) | 4.00 (2.80, 4.10) | 0.5 |
| **Cardiac Index (initial) //Cardiac Index: (L/min/sq.m)** | 3.60 (2.80, 4.60) | 4.90 (4.50, 5.30) | 0.14 |
| **CO (final) (L/min) //CO:** | 7.70 (6.20, 9.40) | 7.25 (4.80, 8.50) | 0.4 |
| **CO (initial) (L/min) //CO: (L/min)** | 7.00 (5.30, 8.70) | 11.15 (11.10, 11.20) | 0.041 |
| **Serum Creatinine** | 1.10 (0.80, 1.70) | 0.80 (0.60, 1.10) | <0.001 |
| **CVP (final) //CVP:** | 9.00 (7.00, 12.00) | 14.00 (9.50, 16.50) | 0.041 |
| **CVP (initial) //CVP: (mm/Hg)** | 9.00 (7.00, 12.00) | 13.50 (6.00, 16.00) | 0.4 |
| **LV Ejection Fraction %** | 60.00 (55.00, 65.00) | 59.00 (45.00, 65.00) | 0.002 |
| **Final Serum Creatinine** | 1.10 (0.80, 1.70) | 0.80 (0.60, 1.09) | <0.001 |
| **Hematocrit:** | 28.80 (25.50, 32.60) | 29.80 (25.40, 35.30) | <0.001 |
| **Donor/s Height in centimeters** | 172.72 (165.00, 178.00) | 173.00 (165.10, 180.00) | <0.001 |
| **INR:** | 1.25 (1.10, 1.40) | 1.20 (1.10, 1.37) | <0.001 |
| **% Glomerulosclerosis (Left Kidney)** | 1.00 (1.00, 2.00) | 1.00 (1.00, 2.00) | <0.001 |
| **Final Resistance Prior to Shipping (left) //Final Resistance Prior to Shipping:** | 0.20 (0.15, 0.27) | 0.20 (0.15, 0.26) | 0.015 |
| **Serum Lipase** | 27.00 (12.00, 67.00) | 43.00 (16.00, 106.00) | <0.001 |
| **% Macro vesicular fat:** | 5.00 (0.00, 10.00) | 5.00 (0.00, 9.50) | <0.001 |
| **% Micro vesicular fat:** | 5.00 (0.00, 15.00) | 0.00 (0.00, 5.00) | <0.001 |
| **MAP (final) //Map:** | 87.00 (77.00, 99.00) | 81.00 (77.00, 90.00) | 0.3 |
| **MAP (initial) //Map: (mm/Hg)** | 86.00 (76.00, 99.00) | 90.00 (83.00, 97.00) | 0.6 |
| **Maximum Donor Creatinine** | 1.10 (0.80, 1.73) | 0.80 (0.60, 1.10) | <0.001 |
| **PA Diastolic (final) //PA Diastolic:** | 17.00 (14.00, 21.00) | 20.00 (13.00, 23.00) | 0.7 |
| **PA Diastolic (initial) //PA Diastolic: (mm/Hg)** | 17.00 (13.00, 22.00) | 22.00 (21.00, 23.00) | 0.2 |
| **PA Systolic (final) //PA Systolic:** | 29.00 (24.00, 36.00) | 30.00 (28.00, 46.00) | 0.4 |
| **PA Systolic (initial) //PA Systolic: (mm/Hg)** | 30.00 (25.00, 37.00) | 37.00 (29.00, 45.00) | 0.4 |
| **pCO2:** | 38.00 (34.00, 42.30) | 38.00 (33.85, 44.00) | 0.2 |
| **PCWP (initial) //PCWP: (mm/Hg)** | 13.00 (10.00, 18.00) | 35.00 (35.00, 35.00) | 0.092 |
| **Peak Serum Creatinine** | 1.50 (1.10, 2.20) | 1.21 (0.94, 1.64) | <0.001 |
| **Blood PH:** | 7.41 (7.36, 7.45) | 7.42 (7.37, 7.46) | <0.001 |
| **Lung pO2 on 100%** | 178.00 (112.00, 380.00) | 124.00 (88.00, 207.50) | <0.001 |
| **Lung pO2 on Fio2 //If Yes, Lung pO2 on FiO2 of:** | 100.00 (50.00, 100.00) | 60.00 (40.00, 100.00) | <0.001 |
| **% Glomerulosclerosis (Right Kidney)** | 1.00 (1.00, 2.00) | 1.00 (1.00, 2.00) | <0.001 |
| **Final Resistance Prior to Shipping (right) //Final Resistance Prior to Shipping:** | 0.20 (0.15, 0.26) | 0.19 (0.14, 0.25) | 0.026 |
| **Serum Amylase** | 60.00 (32.00, 116.00) | 55.00 (31.00, 100.00) | 0.2 |
| **SGOT/AST** | 41.00 (24.00, 82.00) | 54.00 (34.00, 86.00) | <0.001 |
| **SGPT/ALT** | 36.00 (21.00, 75.00) | 41.00 (25.00, 74.00) | <0.001 |
| **Serum Sodium //Last Serum Sodium Prior to Procurement:** | 148.00 (143.00, 154.00) | 147.00 (141.00, 152.00) | <0.001 |
| **SVR (final) //SVR:** | 820.00 (665.00, 1,055.00) | 1,015.50 (823.00, 1,208.00) | 0.4 |
| **SVR (initial) //SVR: (dynes/sec/cm)^5)** | 954.00 (735.90, 1,288.00) | 848.00 (848.00, 848.00) | 0.7 |
| **Total Bilirubin** | 0.70 (0.40, 1.00) | 0.60 (0.40, 0.80) | <0.001 |
| **Troponin I** | 0.17 (0.04, 0.76) | 0.16 (0.04, 1.19) | 0.2 |
| **Troponin T** | 0.05 (0.01, 0.25) | 0.03 (0.01, 0.25) | 0.4 |
| **If Abnormal, # of Vessels with > 50% Stenosis** | 0.00 (0.00, 1.00) | 1.00 (0.00, 1.00) | 0.4 |
| **Estimated Warm Ischemic Time in minutes** | 0.00 (0.00, 0.00) | 18.00 (13.00, 23.00) | <0.001 |
| **Donor/s Weight in kilograms** | 80.00 (68.00, 94.00) | 78.85 (67.00, 91.50) | 0.017 |
| **Donor Risk Index (DRI)** | 1.63 (1.43, 1.92) | 2.24 (2.03, 2.55) | <0.001 |
| **Donor/s Blood Type** |  |  | <0.001 |
| A | 2,412 (14%) | 213 (18%) |  |
| A1 | 3,321 (19%) | 221 (19%) |  |
| A1B | 222 (1.3%) | 9 (0.8%) |  |
| A2 | 668 (3.8%) | 45 (3.8%) |  |
| A2B | 100 (0.6%) | 8 (0.7%) |  |
| AB | 235 (1.4%) | 6 (0.5%) |  |
| B | 1,897 (11%) | 108 (9.2%) |  |
| O | 8,518 (49%) | 564 (48%) |  |
| **CVA cause of death** |  |  | <0.001 |
| No | 11,884 (68%) | 997 (85%) |  |
| Yes | 5,489 (32%) | 177 (15%) |  |
| **history of hypertension** |  |  | <0.001 |
| No | 11,173 (64%) | 921 (78%) |  |
| Yes | 6,062 (35%) | 244 (21%) |  |
| Missing | 138 (0.8%) | 9 (0.8%) |  |
| **Allocate Remaining Liver Segment?** |  |  | 0.4 |
| A | 17,322 (100%) | 1,173 (100%) |  |
| No | 30 (0.2%) | 0 (0%) |  |
| Yes | 21 (0.1%) | 1 (<0.1%) |  |
| **Anti-CMV** |  |  | <0.001 |
| Missing | 0 (0%) | 0 (0%) |  |
| Indeterminate | 61 (0.4%) | 5 (0.4%) |  |
| Negative | 6,279 (36%) | 530 (45%) |  |
| Not Done | 11 (<0.1%) | 0 (0%) |  |
| Positive | 11,022 (63%) | 639 (54%) |  |
| **Anti-HBC** |  |  | <0.001 |
| Indeterminate | 0 (0%) | 1 (<0.1%) |  |
| Negative | 16,527 (95%) | 1,149 (98%) |  |
| Not Done | 8 (<0.1%) | 0 (0%) |  |
| Positive | 838 (4.8%) | 24 (2.0%) |  |
| **Anti-HCV** |  |  | 0.12 |
| Missing | 0 (0%) | 0 (0%) |  |
| Indeterminate | 1 (<0.1%) | 0 (0%) |  |
| Negative | 16,316 (94%) | 1,134 (97%) |  |
| Not Done | 1 (<0.1%) | 0 (0%) |  |
| Positive | 1,055 (6.1%) | 40 (3.4%) |  |
| **Anti-HIV I/II:** |  |  | 0.035 |
| Negative | 17,182 (99%) | 1,153 (98%) |  |
| Not Done | 176 (1.0%) | 21 (1.8%) |  |
| Positive | 15 (<0.1%) | 0 (0%) |  |
| **Anti-HTLV I/II:** |  |  | 0.3 |
| Cannot Disclose | 1 (<0.1%) | 0 (0%) |  |
| Indeterminate | 1 (<0.1%) | 0 (0%) |  |
| Negative | 2,765 (16%) | 195 (17%) |  |
| Not Done | 14,597 (84%) | 977 (83%) |  |
| Positive | 5 (<0.1%) | 1 (<0.1%) |  |
| Unknown | 4 (<0.1%) | 1 (<0.1%) |  |
| **Arginine Vasopressin:** |  |  | <0.001 |
| Missing | 1 (<0.1%) | 0 (0%) |  |
| Negative / No | 6,100 (35%) | 913 (78%) |  |
| Positive / Yes | 11,268 (65%) | 261 (22%) |  |
| Unknown | 4 (<0.1%) | 0 (0%) |  |
| **Donor/s Cause of Death** |  |  | <0.001 |
| Anoxia | 6,139 (35%) | 592 (50%) |  |
| Cerebrovascular / Stroke | 5,512 (32%) | 174 (15%) |  |
| CNS Tumor | 54 (0.3%) | 0 (0%) |  |
| Head Trauma | 5,317 (31%) | 359 (31%) |  |
| Other | 351 (2.0%) | 49 (4.2%) |  |
| **Cardiac Arr. Since Event that led to Decl of Death** |  |  | <0.001 |
| Missing | 2 (<0.1%) | 876 (75%) |  |
| No | 16,149 (93%) | 283 (24%) |  |
| Yes | 1,222 (7.0%) | 15 (1.3%) |  |
| **Donor RNA Nat serology value for Chagas test** |  |  | 0.004 |
| Missing | 5,125 (29%) | 276 (24%) |  |
| Cannot Disclose | 1 (<0.1%) | 0 (0%) |  |
| Negative | 29 (0.2%) | 0 (0%) |  |
| Not Done | 12,212 (70%) | 898 (76%) |  |
| Unknown | 6 (<0.1%) | 0 (0%) |  |
| **Chest X-ray:** |  |  | 0.093 |
| No chest x-ray | 14 (<0.1%) | 1 (<0.1%) |  |
| Normal | 3,414 (20%) | 197 (17%) |  |
| Abnormal-left | 1,776 (10%) | 115 (9.8%) |  |
| Abnormal-right | 1,736 (10.0%) | 107 (9.1%) |  |
| Abnormal-both | 9,677 (56%) | 703 (60%) |  |
| Results Unknown | 8 (<0.1%) | 1 (<0.1%) |  |
| Missing | 748 (4.3%) | 50 (4.3%) |  |
| **Donor/s Citizenship** |  |  | 0.007 |
| US Citizen | 15,879 (91%) | 1,103 (94%) |  |
| Non-US Citizen/US Resident | 578 (3.3%) | 19 (1.6%) |  |
| Non-US Citizen/Non-US Resident | 60 (0.3%) | 3 (0.3%) |  |
| Unknown | 856 (4.9%) | 49 (4.2%) |  |
| **Clamp Time Zone** |  |  | <0.001 |
| Eastern | 8,254 (48%) | 518 (44%) |  |
| Central | 5,707 (33%) | 415 (35%) |  |
| Mountain | 912 (5.2%) | 85 (7.2%) |  |
| Pacific | 2,126 (12%) | 155 (13%) |  |
| Alaska | 38 (0.2%) | 1 (<0.1%) |  |
| Hawaii | 57 (0.3%) | 0 (0%) |  |
| Atlantic | 279 (1.6%) | 0 (0%) |  |
| **Clinical Infection** |  |  | 0.019 |
| Negative / No | 4,424 (25%) | 334 (28%) |  |
| Positive / Yes | 12,923 (74%) | 836 (71%) |  |
| Unknown | 26 (0.1%) | 4 (0.3%) |  |
| **Donor cause of death is stroke (1= yes, 0= no)** |  |  | <0.001 |
| No | 11,798 (68%) | 995 (85%) |  |
| Yes | 5,575 (32%) | 179 (15%) |  |
| **Donor/s Continued Use in Last 6 Months** |  |  | 0.011 |
| Missing | 13,753 (79%) | 976 (83%) |  |
| Negative / No | 579 (3.3%) | 24 (2.0%) |  |
| Positive / Yes | 3,026 (17%) | 173 (15%) |  |
| Unknown | 15 (<0.1%) | 1 (<0.1%) |  |
| **Cocaine Use Last 6 Months** |  |  | 0.2 |
| Missing | 13,882 (80%) | 953 (81%) |  |
| Negative / No | 1,348 (7.8%) | 91 (7.8%) |  |
| Positive / Yes | 1,732 (10.0%) | 97 (8.3%) |  |
| Unknown | 411 (2.4%) | 33 (2.8%) |  |
| **Other Drug Abuse Last 6 Months** |  |  | 0.002 |
| Missing | 9,478 (55%) | 578 (49%) |  |
| Negative / No | 1,530 (8.8%) | 114 (9.7%) |  |
| Positive / Yes | 5,681 (33%) | 414 (35%) |  |
| Unknown | 684 (3.9%) | 68 (5.8%) |  |
| **Coronary Angiogram** |  |  | <0.001 |
| No | 13,472 (78%) | 1,158 (99%) |  |
| Yes, normal | 2,883 (17%) | 5 (0.4%) |  |
| Yes, not normal | 1,018 (5.9%) | 11 (0.9%) |  |
| **If not DCD, Was this a consented DCD donor that progressed to brain death?** |  |  | <0.001 |
| Missing | 6 (<0.1%) | 1,174 (100%) |  |
| No | 16,301 (94%) | 0 (0%) |  |
| Yes | 1,066 (6.1%) | 0 (0%) |  |
| **Meds given Donor: DDAVP** |  |  | 0.5 |
| Missing | 1 (<0.1%) | 0 (0%) |  |
| Negative / No | 15,729 (91%) | 1,073 (91%) |  |
| Positive / Yes | 1,635 (9.4%) | 101 (8.6%) |  |
| Unknown | 8 (<0.1%) | 0 (0%) |  |
| **Cirumstances of Death** |  |  | <0.001 |
| MVA | 2,566 (15%) | 234 (20%) |  |
| SUICIDE | 1,827 (11%) | 208 (18%) |  |
| HOMICIDE | 939 (5.4%) | 48 (4.1%) |  |
| CHILD-ABUSE | 1 (<0.1%) | 0 (0%) |  |
| NON-MVA | 2,173 (13%) | 152 (13%) |  |
| DEATH FROM NATURAL CAUSES | 7,434 (43%) | 385 (33%) |  |
| NONE OF THE ABOVE | 2,433 (14%) | 147 (13%) |  |
| **Mechanism of Death** |  |  | <0.001 |
| DROWNING | 92 (0.5%) | 13 (1.1%) |  |
| SEIZURE | 137 (0.8%) | 14 (1.2%) |  |
| DRUG INTOXICATION | 2,053 (12%) | 140 (12%) |  |
| ASPHYXIATION | 781 (4.5%) | 152 (13%) |  |
| CARDIOVASCULAR | 2,720 (16%) | 227 (19%) |  |
| ELECTRICAL | 11 (<0.1%) | 2 (0.2%) |  |
| GUNSHOT WOUND | 1,792 (10%) | 101 (8.6%) |  |
| STAB | 32 (0.2%) | 7 (0.6%) |  |
| BLUNT INJURY | 3,407 (20%) | 269 (23%) |  |
| INTRACRANIAL HEMORRHAGE/STROKE | 5,592 (32%) | 180 (15%) |  |
| DEATH FROM NATURAL CAUSES | 445 (2.6%) | 46 (3.9%) |  |
| NONE OF THE ABOVE | 311 (1.8%) | 23 (2.0%) |  |
| **EBNA (Epstein-Barr nuclear antigen)** |  |  | <0.001 |
| Cannot Disclose | 2 (<0.1%) | 1 (<0.1%) |  |
| Indeterminate | 12 (<0.1%) | 3 (0.3%) |  |
| Negative | 308 (1.8%) | 43 (3.7%) |  |
| Not Done | 14,474 (83%) | 908 (77%) |  |
| Positive | 2,573 (15%) | 219 (19%) |  |
| Unknown | 4 (<0.1%) | 0 (0%) |  |
| **EBV//IgG:** |  |  | <0.001 |
| Indeterminate | 89 (0.5%) | 2 (0.2%) |  |
| Negative | 785 (4.5%) | 83 (7.1%) |  |
| Not Done | 743 (4.3%) | 65 (5.5%) |  |
| Positive | 15,756 (91%) | 1,024 (87%) |  |
| **EBV//IgM:** |  |  | <0.001 |
| Cannot Disclose | 1 (<0.1%) | 0 (0%) |  |
| Indeterminate | 48 (0.3%) | 3 (0.3%) |  |
| Negative | 14,351 (83%) | 879 (75%) |  |
| Not Done | 2,748 (16%) | 277 (24%) |  |
| Positive | 225 (1.3%) | 15 (1.3%) |  |
| **Method** |  |  | 0.8 |
| Echo | 13,151 (76%) | 206 (18%) |  |
| MUGA | 1 (<0.1%) | 0 (0%) |  |
| Angiogram | 248 (1.4%) | 3 (0.3%) |  |
| Missing | 3,973 (23%) | 965 (82%) |  |
| **SRTR Donor Ethnicity** |  |  | <0.001 |
| Latino | 2,571 (15%) | 108 (9.2%) |  |
| Non-Latino or unknown | 14,802 (85%) | 1,066 (91%) |  |
| **Does donor meet criteria to be an Expanded Donor?** |  |  | <0.001 |
| No | 13,257 (76%) | 1,103 (94%) |  |
| Yes | 4,101 (24%) | 68 (5.8%) |  |
| Missing | 15 (<0.1%) | 3 (0.3%) |  |
| **Meets expanded donor criteria for kidney (1= yes , 0= no)** |  |  | <0.001 |
| No | 13,246 (76%) | 1,104 (94%) |  |
| Yes | 4,127 (24%) | 70 (6.0%) |  |
| **Did the patient express to family or others the intent to be a donor** |  |  | <0.001 |
| Negative / No | 4,940 (28%) | 327 (28%) |  |
| Positive / Yes | 5,245 (30%) | 453 (39%) |  |
| Unknown | 7,188 (41%) | 394 (34%) |  |
| **Donor/s Gender** |  |  | <0.001 |
| Female | 6,944 (40%) | 393 (33%) |  |
| Male | 10,429 (60%) | 781 (67%) |  |
| **Hepatitis B Core Antibody Status** |  |  | 0.007 |
| Negative | 16,520 (95%) | 1,147 (98%) |  |
| Not Done | 5 (<0.1%) | 0 (0%) |  |
| Pending | 9 (<0.1%) | 3 (0.3%) |  |
| Positive | 834 (4.8%) | 24 (2.0%) |  |
| Unknown | 2 (<0.1%) | 0 (0%) |  |
| Missing | 3 (<0.1%) | 0 (0%) |  |
| **Donor RNA Nat serology value for hbv test** |  |  | 0.006 |
| Missing | 5,125 (29%) | 276 (24%) |  |
| Indeterminate | 2 (<0.1%) | 0 (0%) |  |
| Negative | 12,096 (70%) | 881 (75%) |  |
| Not Done | 119 (0.7%) | 16 (1.4%) |  |
| Positive | 30 (0.2%) | 1 (<0.1%) |  |
| Unknown | 1 (<0.1%) | 0 (0%) |  |
| **HBsAb (Hepatitis B Surface Antibody)** |  |  | 0.027 |
| Cannot Disclose | 3 (<0.1%) | 0 (0%) |  |
| Indeterminate | 39 (0.2%) | 3 (0.3%) |  |
| Negative | 2,106 (12%) | 107 (9.1%) |  |
| Not Done | 14,238 (82%) | 1,011 (86%) |  |
| Positive | 984 (5.7%) | 53 (4.5%) |  |
| Unknown | 3 (<0.1%) | 0 (0%) |  |
| **HBV//Surface Antigen:** |  |  | 0.5 |
| Negative | 17,347 (100%) | 1,172 (100%) |  |
| Not Done | 14 (<0.1%) | 2 (0.2%) |  |
| Positive | 12 (<0.1%) | 0 (0%) |  |
| **Donor RNA Nat serology value for hcv test** |  |  | 0.004 |
| Missing | 5,125 (29%) | 276 (24%) |  |
| Indeterminate | 3 (<0.1%) | 0 (0%) |  |
| Negative | 11,654 (67%) | 870 (74%) |  |
| Not Done | 18 (0.1%) | 2 (0.2%) |  |
| Positive | 572 (3.3%) | 26 (2.2%) |  |
| Unknown | 1 (<0.1%) | 0 (0%) |  |
| **HCV Antibody Status** |  |  | <0.001 |
| Indeterminate | 1 (<0.1%) | 0 (0%) |  |
| Negative | 16,305 (94%) | 1,130 (96%) |  |
| Pending | 9 (<0.1%) | 4 (0.3%) |  |
| Positive | 1,056 (6.1%) | 40 (3.4%) |  |
| Missing | 2 (<0.1%) | 0 (0%) |  |
| **Heavy Alcohol Use (heavy= 2+ drinks/day):** |  |  | 0.2 |
| Negative / No | 14,210 (82%) | 938 (80%) |  |
| Positive / Yes | 2,800 (16%) | 211 (18%) |  |
| Unknown | 363 (2.1%) | 25 (2.1%) |  |
| **Heparin** |  |  | 0.2 |
| Negative / No | 321 (1.8%) | 32 (2.7%) |  |
| Positive / Yes | 17,050 (98%) | 1,142 (97%) |  |
| Unknown | 2 (<0.1%) | 0 (0%) |  |
| **Donor serum creatinine > 1.5 (1= yes, 0= no)** |  |  | <0.001 |
| No | 12,154 (70%) | 1,028 (88%) |  |
| Yes | 5,219 (30%) | 146 (12%) |  |
| **History of Cancer** |  |  | 0.6 |
| NO | 16,692 (96%) | 1,147 (98%) |  |
| CNS TUMOR - OTHER | 18 (0.1%) | 2 (0.2%) |  |
| GENITOURINARY - BLADDER | 14 (<0.1%) | 0 (0%) |  |
| GENITOURINARY - UTERINE CERVIX | 44 (0.3%) | 2 (0.2%) |  |
| GENITOURINARY - UTERINE BODY ENDOMETRIAL | 5 (<0.1%) | 0 (0%) |  |
| GENITOURINARY - VULVA | 1 (<0.1%) | 0 (0%) |  |
| GENITOURINARY - OVARIAN | 19 (0.1%) | 1 (<0.1%) |  |
| GENITOURINARY - PENIS, TESTICULAR | 9 (<0.1%) | 1 (<0.1%) |  |
| SKIN - SQUAMOUS, BASAL CELL | 176 (1.0%) | 7 (0.6%) |  |
| GENITOURINARY - PROSTATE | 37 (0.2%) | 1 (<0.1%) |  |
| GENITOURINARY - KIDNEY | 9 (<0.1%) | 0 (0%) |  |
| GENITOURINARY - UNKNOWN | 1 (<0.1%) | 0 (0%) |  |
| GASTROINTESTINAL - STOMACH | 4 (<0.1%) | 0 (0%) |  |
| GASTROINTESTINAL - COLO-RECTAL | 7 (<0.1%) | 0 (0%) |  |
| GASTROINTESTINAL - PANCREAS | 1 (<0.1%) | 0 (0%) |  |
| BREAST | 39 (0.2%) | 1 (<0.1%) |  |
| SKIN - MELANOMA | 15 (<0.1%) | 0 (0%) |  |
| THYROID | 17 (<0.1%) | 2 (0.2%) |  |
| TONGUE/THROAT | 11 (<0.1%) | 0 (0%) |  |
| LARYNX | 1 (<0.1%) | 0 (0%) |  |
| LUNG (include bronchial) | 1 (<0.1%) | 0 (0%) |  |
| LEUKEMIA/LYMPHOMA | 12 (<0.1%) | 1 (<0.1%) |  |
| CNS TUMOR - ASTROCYTOMA | 7 (<0.1%) | 0 (0%) |  |
| CNS TUMOR - GLIOBLASTOMA MULTIFORME | 4 (<0.1%) | 0 (0%) |  |
| CNS TUMOR - MEDULLOBLASTOMA | 1 (<0.1%) | 1 (<0.1%) |  |
| CNS TUMOR - MENINGIOMA | 7 (<0.1%) | 0 (0%) |  |
| UNKNOWN | 115 (0.7%) | 5 (0.4%) |  |
| OTHER, SPECIFY | 106 (0.6%) | 3 (0.3%) |  |
| **Cigarette Use > 20 Pack Years - Ever** |  |  | <0.001 |
| Missing | 0 (0%) | 0 (0%) |  |
| Negative / No | 13,435 (77%) | 964 (82%) |  |
| Positive / Yes | 3,620 (21%) | 198 (17%) |  |
| Unknown | 318 (1.8%) | 12 (1.0%) |  |
| **Cocaine Use** |  |  | 0.4 |
| Missing | 0 (0%) | 0 (0%) |  |
| Negative / No | 13,585 (78%) | 937 (80%) |  |
| Positive / Yes | 3,491 (20%) | 221 (19%) |  |
| Unknown | 297 (1.7%) | 16 (1.4%) |  |
| **History of Diabetes** |  |  | <0.001 |
| No | 15,218 (88%) | 1,089 (93%) |  |
| Unknown | 110 (0.6%) | 9 (0.8%) |  |
| Yes, >10 years | 769 (4.4%) | 29 (2.5%) |  |
| Yes, 0-5 years | 663 (3.8%) | 28 (2.4%) |  |
| Yes, 6-10 years | 394 (2.3%) | 13 (1.1%) |  |
| Yes, Duration Unknown | 219 (1.3%) | 6 (0.5%) |  |
| **History of Hypertension** |  |  | <0.001 |
| No | 11,153 (64%) | 919 (78%) |  |
| Unknown | 124 (0.7%) | 8 (0.7%) |  |
| Yes, >10 years | 1,678 (9.7%) | 57 (4.9%) |  |
| Yes, 0-5 years | 2,497 (14%) | 121 (10%) |  |
| Yes, 6-10 years | 1,171 (6.7%) | 43 (3.7%) |  |
| Yes, Duration Unknown | 750 (4.3%) | 26 (2.2%) |  |
| **Insulin Dependent** |  |  | 0.2 |
| No | 991 (5.7%) | 27 (2.3%) |  |
| Unknown | 143 (0.8%) | 5 (0.4%) |  |
| Yes, >10 years | 394 (2.3%) | 18 (1.5%) |  |
| Yes, 0-5 years | 240 (1.4%) | 14 (1.2%) |  |
| Yes, 6-10 years | 122 (0.7%) | 6 (0.5%) |  |
| Yes, Duration Unknown | 155 (0.9%) | 6 (0.5%) |  |
| Missing | 15,328 (88%) | 1,098 (94%) |  |
| **Other Drug Abuse** |  |  | 0.001 |
| Missing | 0 (0%) | 0 (0%) |  |
| Negative / No | 9,281 (53%) | 569 (48%) |  |
| Positive / Yes | 7,895 (45%) | 596 (51%) |  |
| Unknown | 197 (1.1%) | 9 (0.8%) |  |
| **History of Previous MI** |  |  | 0.029 |
| Negative / No | 16,555 (95%) | 1,138 (97%) |  |
| Positive / Yes | 665 (3.8%) | 27 (2.3%) |  |
| Unknown | 153 (0.9%) | 9 (0.8%) |  |
| **Donor RNA Nat serology value for hiv test** |  |  | 0.001 |
| Missing | 5,125 (29%) | 276 (24%) |  |
| Indeterminate | 2 (<0.1%) | 0 (0%) |  |
| Negative | 12,173 (70%) | 885 (75%) |  |
| Not Done | 62 (0.4%) | 13 (1.1%) |  |
| Positive | 10 (<0.1%) | 0 (0%) |  |
| Unknown | 1 (<0.1%) | 0 (0%) |  |
| **Donor/s Home State** |  |  | <0.001 |
| ALASKA | 38 (0.2%) | 2 (0.2%) |  |
| ALABAMA | 359 (2.1%) | 10 (0.9%) |  |
| ARKANSAS | 169 (1.0%) | 10 (0.9%) |  |
| PAGO PAGO | 1 (<0.1%) | 0 (0%) |  |
| ARIZONA | 339 (2.0%) | 41 (3.5%) |  |
| CALIFORNIA | 1,596 (9.2%) | 89 (7.6%) |  |
| COLORADO | 153 (0.9%) | 9 (0.8%) |  |
| CONNECTICUT | 147 (0.8%) | 0 (0%) |  |
| DISTRICT OF COLUMBIA | 35 (0.2%) | 1 (<0.1%) |  |
| DELAWARE | 93 (0.5%) | 3 (0.3%) |  |
| FLORIDA | 1,297 (7.5%) | 112 (9.5%) |  |
| GEORGIA | 700 (4.0%) | 35 (3.0%) |  |
| GUAM | 1 (<0.1%) | 0 (0%) |  |
| HAWAII | 56 (0.3%) | 0 (0%) |  |
| IOWA | 148 (0.9%) | 11 (0.9%) |  |
| IDAHO | 68 (0.4%) | 15 (1.3%) |  |
| ILLINOIS | 741 (4.3%) | 63 (5.4%) |  |
| INDIANA | 463 (2.7%) | 50 (4.3%) |  |
| KANSAS | 189 (1.1%) | 20 (1.7%) |  |
| KENTUCKY | 244 (1.4%) | 14 (1.2%) |  |
| LOUISIANA | 423 (2.4%) | 20 (1.7%) |  |
| MASSACHUSETTS | 259 (1.5%) | 13 (1.1%) |  |
| MARYLAND | 409 (2.4%) | 26 (2.2%) |  |
| MAINE | 56 (0.3%) | 3 (0.3%) |  |
| MICHIGAN | 497 (2.9%) | 56 (4.8%) |  |
| MINNESOTA | 174 (1.0%) | 21 (1.8%) |  |
| MISSOURI | 476 (2.7%) | 15 (1.3%) |  |
| MISSISSIPPI | 212 (1.2%) | 11 (0.9%) |  |
| MONTANA | 36 (0.2%) | 2 (0.2%) |  |
| FOREIGN COUNTRY | 6 (<0.1%) | 0 (0%) |  |
| NORTH CAROLINA | 484 (2.8%) | 30 (2.6%) |  |
| NORTH DAKOTA | 23 (0.1%) | 2 (0.2%) |  |
| NEBRASKA | 106 (0.6%) | 10 (0.9%) |  |
| NEW HAMPSHIRE | 56 (0.3%) | 2 (0.2%) |  |
| NEW JERSEY | 418 (2.4%) | 14 (1.2%) |  |
| NEW MEXICO | 97 (0.6%) | 9 (0.8%) |  |
| NEVADA | 233 (1.3%) | 21 (1.8%) |  |
| NEW YORK | 600 (3.5%) | 25 (2.1%) |  |
| OHIO | 682 (3.9%) | 75 (6.4%) |  |
| OKLAHOMA | 295 (1.7%) | 28 (2.4%) |  |
| OREGON | 76 (0.4%) | 10 (0.9%) |  |
| PENNSYLVANIA | 924 (5.3%) | 44 (3.7%) |  |
| PUERTO RICO | 274 (1.6%) | 0 (0%) |  |
| RHODE ISLAND | 27 (0.2%) | 1 (<0.1%) |  |
| SOUTH CAROLINA | 321 (1.8%) | 12 (1.0%) |  |
| SOUTH DAKOTA | 31 (0.2%) | 5 (0.4%) |  |
| TENNESSEE | 526 (3.0%) | 24 (2.0%) |  |
| TEXAS | 1,543 (8.9%) | 83 (7.1%) |  |
| UTAH | 146 (0.8%) | 10 (0.9%) |  |
| VIRGINIA | 351 (2.0%) | 21 (1.8%) |  |
| VIRGIN ISLANDS | 1 (<0.1%) | 0 (0%) |  |
| VERMONT | 23 (0.1%) | 1 (<0.1%) |  |
| WASHINGTON | 196 (1.1%) | 31 (2.6%) |  |
| WISCONSIN | 352 (2.0%) | 55 (4.7%) |  |
| WEST VIRGINIA | 116 (0.7%) | 2 (0.2%) |  |
| WYOMING | 23 (0.1%) | 1 (<0.1%) |  |
| UNKNOWN | 64 (0.4%) | 6 (0.5%) |  |
| **Donor RNA Nat serology value for htlv test** |  |  | 0.029 |
| Missing | 5,125 (29%) | 276 (24%) |  |
| Cannot Disclose | 1 (<0.1%) | 0 (0%) |  |
| Indeterminate | 1 (<0.1%) | 0 (0%) |  |
| Negative | 38 (0.2%) | 5 (0.4%) |  |
| Not Done | 12,202 (70%) | 893 (76%) |  |
| Unknown | 6 (<0.1%) | 0 (0%) |  |
| **Donor history of hypertension (1= yes, 0= no)** |  |  | <0.001 |
| No | 11,131 (64%) | 919 (78%) |  |
| Yes | 6,119 (35%) | 247 (21%) |  |
| Missing | 123 (0.7%) | 8 (0.7%) |  |
| **Infection Source: Blood** |  |  | 0.2 |
| No | 15,545 (89%) | 1,036 (88%) |  |
| Yes | 1,828 (11%) | 138 (12%) |  |
| **Infection Source: Blood/Confirmed by Culture** |  |  | 0.2 |
| Missing | 16,850 (97%) | 1,147 (98%) |  |
| No | 34 (0.2%) | 0 (0%) |  |
| Yes | 489 (2.8%) | 27 (2.3%) |  |
| **Infection Source: Lung** |  |  | 0.001 |
| No | 6,116 (35%) | 472 (40%) |  |
| Yes | 11,257 (65%) | 702 (60%) |  |
| **Infection Source: Lung/Confirmed by Culture** |  |  | <0.001 |
| Missing | 14,096 (81%) | 1,011 (86%) |  |
| No | 70 (0.4%) | 1 (<0.1%) |  |
| Yes | 3,207 (18%) | 162 (14%) |  |
| **Infection Source: Other** |  |  | 0.7 |
| No | 16,078 (93%) | 1,083 (92%) |  |
| Yes | 1,295 (7.5%) | 91 (7.8%) |  |
| **Infection Source: Other/Confimed by Culture** |  |  | 0.2 |
| Missing | 16,988 (98%) | 1,155 (98%) |  |
| No | 22 (0.1%) | 0 (0%) |  |
| Yes | 363 (2.1%) | 19 (1.6%) |  |
| **Infection Source: Urine** |  |  | 0.2 |
| No | 14,619 (84%) | 972 (83%) |  |
| Yes | 2,754 (16%) | 202 (17%) |  |
| **Infection Source: Urine/Confirmed by Culture** |  |  | 0.14 |
| Missing | 16,505 (95%) | 1,127 (96%) |  |
| No | 35 (0.2%) | 0 (0%) |  |
| Yes | 833 (4.8%) | 47 (4.0%) |  |
| **Three or More Inotropic Agents at Time of Incision** |  |  | <0.001 |
| Missing | 12,063 (69%) | 887 (76%) |  |
| No | 5,232 (30%) | 287 (24%) |  |
| Yes | 78 (0.4%) | 0 (0%) |  |
| **Inotropic Support** |  |  | <0.001 |
| Missing | 0 (0%) | 0 (0%) |  |
| Negative / No | 8,922 (51%) | 1,135 (97%) |  |
| Positive / Yes | 8,436 (49%) | 39 (3.3%) |  |
| Unknown | 15 (<0.1%) | 0 (0%) |  |
| **Insulin:** |  |  | <0.001 |
| Missing | 0 (0%) | 0 (0%) |  |
| Negative / No | 6,701 (39%) | 930 (79%) |  |
| Positive / Yes | 10,665 (61%) | 244 (21%) |  |
| Unknown | 7 (<0.1%) | 0 (0%) |  |
| **Left Lung Machine Perfusion Intended or Performed:** |  |  | <0.001 |
| Missing | 11,802 (68%) | 864 (74%) |  |
| No | 5,431 (31%) | 287 (24%) |  |
| Yes | 140 (0.8%) | 23 (2.0%) |  |
| **Was the patient declared legally brain dead:** |  |  | <0.001 |
| Missing | 4,715 (27%) | 261 (22%) |  |
| No | 2 (<0.1%) | 887 (76%) |  |
| Yes | 12,656 (73%) | 26 (2.2%) |  |
| **Kidney Biopsy (left)** |  |  | 0.009 |
| Missing | 383 (2.2%) | 14 (1.2%) |  |
| No | 9,607 (55%) | 617 (53%) |  |
| Yes | 7,383 (42%) | 543 (46%) |  |
| **Pump (Left Kidney)** |  |  | <0.001 |
| Missing | 400 (2.3%) | 15 (1.3%) |  |
| No | 12,779 (74%) | 500 (43%) |  |
| Yes | 4,194 (24%) | 659 (56%) |  |
| **Transferred on pump (left)://Transferred on pump:** |  |  | <0.001 |
| Missing | 13,180 (76%) | 515 (44%) |  |
| No | 1,157 (6.7%) | 188 (16%) |  |
| Yes | 3,036 (17%) | 471 (40%) |  |
| **Left Lung Bronchoscopy:** |  |  | <0.001 |
| No Bronchoscopy | 754 (4.3%) | 131 (11%) |  |
| Bronchoscopy Results normal | 4,468 (26%) | 91 (7.8%) |  |
| Bronchoscopy Results, Abnormal-purulent secretions | 1,121 (6.5%) | 20 (1.7%) |  |
| Bronchoscopy Results, Abnormal-aspiration of foreign body | 48 (0.3%) | 1 (<0.1%) |  |
| Bronchoscopy Results, Abnormal-blood | 394 (2.3%) | 2 (0.2%) |  |
| Bronchoscopy Results, Abnormal-anatomy/other lesion | 106 (0.6%) | 5 (0.4%) |  |
| Bronchoscopy Results, Unknown | 19 (0.1%) | 3 (0.3%) |  |
| Unknown if bronchoscopy performed | 7 (<0.1%) | 0 (0%) |  |
| Missing | 10,456 (60%) | 921 (78%) |  |
| **Liver Biopsy** |  |  | <0.001 |
| Missing | 0 (0%) | 0 (0%) |  |
| No | 10,680 (61%) | 917 (78%) |  |
| Yes | 6,693 (39%) | 257 (22%) |  |
| **Granulomas Histology performed** |  |  | 0.7 |
| No | 5,301 (31%) | 288 (25%) |  |
| Yes | 13 (<0.1%) | 0 (0%) |  |
| Missing | 12,059 (69%) | 886 (75%) |  |
| **Hemosidera Histology performed** |  |  | 0.6 |
| No | 5,299 (31%) | 288 (25%) |  |
| Yes | 15 (<0.1%) | 0 (0%) |  |
| Missing | 12,059 (69%) | 886 (75%) |  |
| **Other Histology performed** |  |  | <0.001 |
| No | 4,410 (25%) | 268 (23%) |  |
| Yes | 904 (5.2%) | 20 (1.7%) |  |
| Missing | 12,059 (69%) | 886 (75%) |  |
| **Medical Examiner/Coroner** |  |  | <0.001 |
| NO | 6,076 (35%) | 312 (27%) |  |
| YES, MEDICAL EXAMINER CONSENTED | 11,212 (65%) | 851 (72%) |  |
| YES, MEDICAL EXAMINER REFUSED CONSENT | 24 (0.1%) | 3 (0.3%) |  |
| UNKNOWN | 61 (0.4%) | 8 (0.7%) |  |
| **Does the Donor meet CDC guidelines for High Risk for an organ donor:** |  |  | 0.3 |
| Missing | 0 (0%) | 0 (0%) |  |
| Negative / No | 13,086 (75%) | 904 (77%) |  |
| Positive / Yes | 4,285 (25%) | 270 (23%) |  |
| Unknown | 2 (<0.1%) | 0 (0%) |  |
| **Non-Heart Beating Donor** |  |  | <0.001 |
| Missing | 0 (0%) | 0 (0%) |  |
| No | 17,367 (100%) | 0 (0%) |  |
| Yes | 6 (<0.1%) | 1,174 (100%) |  |
| **Controlled** |  |  | <0.001 |
| Missing | 17,367 (100%) | 0 (0%) |  |
| Negative / No | 6 (<0.1%) | 0 (0%) |  |
| Positive / Yes | 0 (0%) | 1,174 (100%) |  |
| **Core Cooling Used** |  |  | <0.001 |
| Missing | 17,366 (100%) | 0 (0%) |  |
| No | 5 (<0.1%) | 425 (36%) |  |
| Yes | 2 (<0.1%) | 749 (64%) |  |
| **Organ was used in a shared 1 (vs local 0) TX** |  |  | <0.001 |
| No | 10,414 (60%) | 764 (65%) |  |
| Yes | 6,959 (40%) | 410 (35%) |  |
| **Lung - Was pO2 done:** |  |  | <0.001 |
| Missing | 12,062 (69%) | 887 (76%) |  |
| Negative / No | 31 (0.2%) | 2 (0.2%) |  |
| Positive / Yes | 5,280 (30%) | 285 (24%) |  |
| **Pre-Recov Meds given Donor: Diuretics** |  |  | <0.001 |
| Missing | 1 (<0.1%) | 0 (0%) |  |
| Negative / No | 5,545 (32%) | 942 (80%) |  |
| Positive / Yes | 11,822 (68%) | 232 (20%) |  |
| Unknown | 5 (<0.1%) | 0 (0%) |  |
| **Pre-Recov Meds given Donor: Steroids** |  |  | <0.001 |
| Missing | 1 (<0.1%) | 0 (0%) |  |
| Negative / No | 4,851 (28%) | 939 (80%) |  |
| Positive / Yes | 12,512 (72%) | 235 (20%) |  |
| Unknown | 9 (<0.1%) | 0 (0%) |  |
| **Pre-Recov Meds given Donor: T3** |  |  | 0.3 |
| Missing | 1 (<0.1%) | 0 (0%) |  |
| Negative / No | 17,333 (100%) | 1,174 (100%) |  |
| Positive / Yes | 33 (0.2%) | 0 (0%) |  |
| Unknown | 6 (<0.1%) | 0 (0%) |  |
| **Pre-Recov Meds given Donor: T4** |  |  | <0.001 |
| Missing | 1 (<0.1%) | 0 (0%) |  |
| Negative / No | 5,853 (34%) | 1,104 (94%) |  |
| Positive / Yes | 11,516 (66%) | 70 (6.0%) |  |
| Unknown | 3 (<0.1%) | 0 (0%) |  |
| **Previous Gastrointestinal Disease** |  |  | 0.2 |
| Missing | 11 (<0.1%) | 0 (0%) |  |
| Negative / No | 16,092 (93%) | 1,106 (94%) |  |
| Positive / Yes | 1,125 (6.5%) | 61 (5.2%) |  |
| Unknown | 145 (0.8%) | 7 (0.6%) |  |
| **Protein in Urine** |  |  | <0.001 |
| Negative / No | 8,943 (51%) | 521 (44%) |  |
| Positive / Yes | 8,248 (47%) | 638 (54%) |  |
| Unknown | 182 (1.0%) | 15 (1.3%) |  |
| **Was a pulmonary artery catheter placed:** |  |  | <0.001 |
| No | 16,578 (95%) | 1,163 (99%) |  |
| Yes | 795 (4.6%) | 11 (0.9%) |  |
| **SRTR Donor Race** |  |  | <0.001 |
| Asian | 406 (2.3%) | 19 (1.6%) |  |
| Black | 3,379 (19%) | 109 (9.3%) |  |
| Multiracial | 42 (0.2%) | 3 (0.3%) |  |
| Native American | 86 (0.5%) | 10 (0.9%) |  |
| Pacific Islander | 49 (0.3%) | 2 (0.2%) |  |
| White | 13,411 (77%) | 1,031 (88%) |  |
| **Recovered Outside the U.S.** |  |  | >0.9 |
| N | 17,371 (100%) | 1,174 (100%) |  |
| Y | 2 (<0.1%) | 0 (0%) |  |
| **Duration of Resuscitation** |  |  | 0.7 |
| 1 | 26 (0.1%) | 1 (<0.1%) |  |
| 10 | 77 (0.4%) | 0 (0%) |  |
| 11 | 29 (0.2%) | 0 (0%) |  |
| 112 | 1 (<0.1%) | 0 (0%) |  |
| 115 | 1 (<0.1%) | 0 (0%) |  |
| 12 | 20 (0.1%) | 1 (<0.1%) |  |
| 120 | 1 (<0.1%) | 0 (0%) |  |
| 13 | 13 (<0.1%) | 0 (0%) |  |
| 14 | 13 (<0.1%) | 1 (<0.1%) |  |
| 15 | 78 (0.4%) | 1 (<0.1%) |  |
| 16 | 13 (<0.1%) | 0 (0%) |  |
| 17 | 5 (<0.1%) | 0 (0%) |  |
| 18 | 9 (<0.1%) | 0 (0%) |  |
| 19 | 1 (<0.1%) | 0 (0%) |  |
| 2 | 56 (0.3%) | 0 (0%) |  |
| 20 | 94 (0.5%) | 0 (0%) |  |
| 21 | 9 (<0.1%) | 0 (0%) |  |
| 22 | 17 (<0.1%) | 0 (0%) |  |
| 23 | 6 (<0.1%) | 0 (0%) |  |
| 24 | 6 (<0.1%) | 0 (0%) |  |
| 25 | 27 (0.2%) | 0 (0%) |  |
| 26 | 8 (<0.1%) | 0 (0%) |  |
| 27 | 10 (<0.1%) | 0 (0%) |  |
| 28 | 7 (<0.1%) | 0 (0%) |  |
| 29 | 7 (<0.1%) | 0 (0%) |  |
| 3 | 42 (0.2%) | 0 (0%) |  |
| 30 | 63 (0.4%) | 2 (0.2%) |  |
| 31 | 7 (<0.1%) | 1 (<0.1%) |  |
| 32 | 6 (<0.1%) | 0 (0%) |  |
| 33 | 7 (<0.1%) | 0 (0%) |  |
| 34 | 7 (<0.1%) | 0 (0%) |  |
| 35 | 21 (0.1%) | 0 (0%) |  |
| 36 | 7 (<0.1%) | 0 (0%) |  |
| 37 | 9 (<0.1%) | 0 (0%) |  |
| 38 | 1 (<0.1%) | 0 (0%) |  |
| 39 | 5 (<0.1%) | 0 (0%) |  |
| 4 | 46 (0.3%) | 0 (0%) |  |
| 40 | 24 (0.1%) | 1 (<0.1%) |  |
| 42 | 1 (<0.1%) | 0 (0%) |  |
| 43 | 4 (<0.1%) | 0 (0%) |  |
| 45 | 35 (0.2%) | 0 (0%) |  |
| 46 | 2 (<0.1%) | 0 (0%) |  |
| 47 | 4 (<0.1%) | 0 (0%) |  |
| 48 | 1 (<0.1%) | 0 (0%) |  |
| 49 | 3 (<0.1%) | 0 (0%) |  |
| 5 | 70 (0.4%) | 0 (0%) |  |
| 50 | 9 (<0.1%) | 0 (0%) |  |
| 51 | 1 (<0.1%) | 0 (0%) |  |
| 52 | 1 (<0.1%) | 0 (0%) |  |
| 53 | 3 (<0.1%) | 0 (0%) |  |
| 54 | 1 (<0.1%) | 0 (0%) |  |
| 55 | 4 (<0.1%) | 0 (0%) |  |
| 57 | 3 (<0.1%) | 0 (0%) |  |
| 58 | 3 (<0.1%) | 0 (0%) |  |
| 59 | 1 (<0.1%) | 0 (0%) |  |
| 6 | 26 (0.1%) | 0 (0%) |  |
| 60 | 24 (0.1%) | 1 (<0.1%) |  |
| 62 | 2 (<0.1%) | 0 (0%) |  |
| 63 | 3 (<0.1%) | 0 (0%) |  |
| 65 | 4 (<0.1%) | 0 (0%) |  |
| 68 | 1 (<0.1%) | 0 (0%) |  |
| 7 | 25 (0.1%) | 1 (<0.1%) |  |
| 70 | 1 (<0.1%) | 0 (0%) |  |
| 73 | 1 (<0.1%) | 0 (0%) |  |
| 75 | 2 (<0.1%) | 0 (0%) |  |
| 8 | 26 (0.1%) | 2 (0.2%) |  |
| 83 | 1 (<0.1%) | 0 (0%) |  |
| 85 | 1 (<0.1%) | 0 (0%) |  |
| 9 | 19 (0.1%) | 0 (0%) |  |
| 90 | 5 (<0.1%) | 0 (0%) |  |
| Missing | 16,307 (94%) | 1,162 (99%) |  |
| **Right Lung Machine Perfusion Intended or Performed** |  |  | <0.001 |
| Missing | 11,873 (68%) | 864 (74%) |  |
| No | 5,372 (31%) | 286 (24%) |  |
| Yes | 128 (0.7%) | 24 (2.0%) |  |
| **RPR-VDRL** |  |  | 0.012 |
| Indeterminate | 1 (<0.1%) | 0 (0%) |  |
| Negative | 16,727 (96%) | 1,156 (98%) |  |
| Not Done | 504 (2.9%) | 10 (0.9%) |  |
| Positive | 141 (0.8%) | 8 (0.7%) |  |
| **Kidney Biopsy (right)** |  |  | 0.003 |
| Missing | 397 (2.3%) | 13 (1.1%) |  |
| No | 9,455 (54%) | 609 (52%) |  |
| Yes | 7,521 (43%) | 552 (47%) |  |
| **Pump (Right Kidney)** |  |  | <0.001 |
| Missing | 413 (2.4%) | 14 (1.2%) |  |
| No | 12,674 (73%) | 498 (42%) |  |
| Yes | 4,286 (25%) | 662 (56%) |  |
| **Transferred on pump (right)://Transferred on pump:** |  |  | <0.001 |
| Missing | 13,087 (75%) | 512 (44%) |  |
| No | 1,257 (7.2%) | 185 (16%) |  |
| Yes | 3,029 (17%) | 477 (41%) |  |
| **Right Lung Bronchoscopy:** |  |  | <0.001 |
| No Bronchoscopy | 751 (4.3%) | 136 (12%) |  |
| Bronchoscopy Results normal | 4,229 (24%) | 82 (7.0%) |  |
| Bronchoscopy Results, Abnormal-purulent secretions | 1,206 (6.9%) | 24 (2.0%) |  |
| Bronchoscopy Results, Abnormal-aspiration of foreign body | 69 (0.4%) | 2 (0.2%) |  |
| Bronchoscopy Results, Abnormal-blood | 410 (2.4%) | 4 (0.3%) |  |
| Bronchoscopy Results, Abnormal-anatomy/other lesion | 122 (0.7%) | 5 (0.4%) |  |
| Bronchoscopy Results, Unknown | 18 (0.1%) | 3 (0.3%) |  |
| Unknown if bronchoscopy performed | 5 (<0.1%) | 1 (<0.1%) |  |
| Missing | 10,563 (61%) | 917 (78%) |  |
| **strongyloides test result selection** |  |  | >0.9 |
| Missing | 17,291 (100%) | 1,169 (100%) |  |
| Negative | 13 (<0.1%) | 1 (<0.1%) |  |
| Not Done | 69 (0.4%) | 4 (0.3%) |  |
| **Lifestyle Factors: Tattoos** |  |  | 0.007 |
| Negative / No | 9,497 (55%) | 589 (50%) |  |
| Positive / Yes | 7,854 (45%) | 585 (50%) |  |
| Unknown | 22 (0.1%) | 0 (0%) |  |
| **Number of transfusions during this (terminal) hospitalization:** |  |  | <0.001 |
| NONE | 10,110 (58%) | 791 (67%) |  |
| 1 - 5 | 5,191 (30%) | 272 (23%) |  |
| 6 - 10 | 1,300 (7.5%) | 73 (6.2%) |  |
| GREATER THAN 10 | 772 (4.4%) | 38 (3.2%) |  |
| **Donor/s HLA - Typing Method Class I** |  |  | 0.2 |
| Serology | 11 (<0.1%) | 0 (0%) |  |
| DNA | 15,539 (89%) | 1,076 (92%) |  |
| 3 | 1,557 (9.0%) | 90 (7.7%) |  |
| Missing | 266 (1.5%) | 8 (0.7%) |  |
| **Donor/s HLA - Typing Method Class II** |  |  | 0.4 |
| Serology | 20 (0.1%) | 1 (<0.1%) |  |
| DNA | 15,615 (90%) | 1,078 (92%) |  |
| 3 | 1,472 (8.5%) | 87 (7.4%) |  |
| Missing | 266 (1.5%) | 8 (0.7%) |  |
| **Use Double KI allocation, not Expanded Donor** |  |  | 0.040 |
| Missing | 17,215 (99%) | 1,171 (100%) |  |
| No | 69 (0.4%) | 0 (0%) |  |
| Yes | 89 (0.5%) | 3 (0.3%) |  |
| **Vasodilators** |  |  | 0.075 |
| Missing | 1 (<0.1%) | 0 (0%) |  |
| Negative / No | 14,981 (86%) | 1,052 (90%) |  |
| Positive / Yes | 2,379 (14%) | 122 (10%) |  |
| Unknown | 12 (<0.1%) | 0 (0%) |  |
| **Wall Abnormalities //Global:** |  |  | <0.001 |
| Missing | 15,337 (88%) | 1,118 (95%) |  |
| No | 1,236 (7.1%) | 39 (3.3%) |  |
| Yes | 800 (4.6%) | 17 (1.4%) |  |
| **Wall Abnormalities //Segmental:** |  |  | <0.001 |
| Missing | 15,337 (88%) | 1,118 (95%) |  |
| No | 1,316 (7.6%) | 37 (3.2%) |  |
| Yes | 720 (4.1%) | 19 (1.6%) |  |
| **Donor RNA Nat serology value for West Nile test** |  |  | 0.005 |
| Missing | 5,125 (29%) | 276 (24%) |  |
| Cannot Disclose | 3 (<0.1%) | 0 (0%) |  |
| Negative | 3,998 (23%) | 340 (29%) |  |
| Not Done | 8,241 (47%) | 558 (48%) |  |
| Positive | 2 (<0.1%) | 0 (0%) |  |
| Unknown | 4 (<0.1%) | 0 (0%) |  |
| **^1^Median (Q1, Q3); n (%)** | | | |
| **^2^Wilcoxon rank sum test; Pearson's Chi-squared test with simulated p-value (based on 2000 replicates)** | | | |

# **Table B4. Donor Characteristics by Donor Type, 2019-01-01 to 2023-12-31**

| **Characteristic** | **DBD**  N = 22,444^1^ | **DCD**  N = 2,930^1^ | **p-value**^2^ |
| --- | --- | --- | --- |
| **Calculated Donor Age in Years at Organ Recovery or Referral Date** | 41.00 (29.00, 54.00) | 40.00 (29.00, 51.00) | <0.001 |
| **Donor/s Age in Months** | 492.00 (358.00, 652.00) | 488.00 (355.00, 619.00) | <0.001 |
| **most recent serum creatinine** | 1.10 (0.80, 2.00) | 0.80 (0.60, 1.20) | <0.001 |
| **BUN** | 24.00 (16.00, 39.00) | 19.00 (13.00, 29.00) | <0.001 |
| **Cancer Free Interval:** | 7.00 (1.00, 15.00) | 6.00 (0.00, 11.00) | 0.10 |
| **Cardiac Index (final) //Cardiac Index:** | 3.90 (3.20, 4.60) | 3.65 (2.75, 4.15) | 0.3 |
| **Cardiac Index (initial) //Cardiac Index: (L/min/sq.m)** | 4.25 (3.20, 5.40) | 4.27 (3.90, 4.90) | 0.6 |
| **CO (final) (L/min) //CO:** | 7.70 (6.20, 9.10) | 7.20 (6.10, 8.75) | 0.7 |
| **CO (initial) (L/min) //CO: (L/min)** | 8.00 (6.20, 10.60) | 7.80 (6.40, 9.10) | 0.6 |
| **Serum Creatinine** | 1.10 (0.79, 2.00) | 0.80 (0.60, 1.20) | <0.001 |
| **CVP (final) //CVP:** | 9.00 (7.00, 12.00) | 8.00 (7.00, 18.00) | 0.4 |
| **CVP (initial) //CVP: (mm/Hg)** | 9.00 (6.00, 13.00) | 12.00 (5.50, 13.50) | 0.6 |
| **LV Ejection Fraction %** | 60.00 (55.00, 65.00) | 60.00 (55.00, 65.00) | 0.2 |
| **Final Serum Creatinine** | 1.10 (0.80, 2.00) | 0.80 (0.60, 1.20) | <0.001 |
| **Hematocrit:** | 27.90 (24.90, 31.30) | 30.00 (25.70, 34.40) | <0.001 |
| **Donor/s Height in centimeters** | 172.70 (165.00, 178.00) | 173.00 (165.00, 180.00) | <0.001 |
| **INR:** | 1.21 (1.10, 1.40) | 1.20 (1.10, 1.30) | <0.001 |
| **Glomeruli Observed on Kidney biopsy** | 53.00 (27.00, 74.00) | 46.00 (39.00, 65.00) | 0.8 |
| **Percent Glomerular Sclerosis on Kidney biopsy** | 7.00 (1.00, 15.00) | 3.00 (1.00, 8.00) | 0.2 |
| **% Glomerulosclerosis (Left Kidney)** | 1.00 (1.00, 2.00) | 1.00 (1.00, 2.00) | <0.001 |
| **Final Resistance Prior to Shipping (left) //Final Resistance Prior to Shipping:** | 0.21 (0.15, 0.29) | 0.20 (0.15, 0.27) | <0.001 |
| **Serum Lipase** | 29.50 (14.00, 71.00) | 34.00 (19.00, 73.00) | 0.003 |
| **% Macro vesicular fat:** | 5.00 (0.00, 10.00) | 5.00 (0.00, 10.00) | <0.001 |
| **% Micro vesicular fat:** | 5.00 (0.00, 10.00) | 0.00 (0.00, 5.00) | <0.001 |
| **MAP (final) //Map:** | 88.00 (80.00, 100.00) | 89.00 (78.00, 100.00) | >0.9 |
| **MAP (initial) //Map: (mm/Hg)** | 82.00 (68.00, 97.00) | 83.00 (72.00, 105.00) | 0.5 |
| **Maximum Donor Creatinine** | 1.10 (0.80, 2.00) | 0.80 (0.60, 1.20) | <0.001 |
| **PA Diastolic (final) //PA Diastolic:** | 17.00 (14.00, 23.00) | 22.00 (16.00, 25.00) | 0.4 |
| **PA Diastolic (initial) //PA Diastolic: (mm/Hg)** | 19.00 (15.00, 24.00) | 19.00 (14.00, 28.00) | 0.8 |
| **PA Systolic (final) //PA Systolic:** | 32.00 (26.00, 41.00) | 36.00 (30.00, 57.00) | 0.4 |
| **PA Systolic (initial) //PA Systolic: (mm/Hg)** | 32.00 (27.00, 39.00) | 38.00 (29.00, 40.00) | 0.3 |
| **pCO2:** | 38.60 (35.00, 43.00) | 39.00 (34.00, 44.00) | 0.2 |
| **PCWP (final) //PCWP:** | 15.00 (12.00, 20.00) | 13.00 (13.00, 13.00) | 0.5 |
| **PCWP (initial) //PCWP: (mm/Hg)** | 15.00 (13.00, 19.00) | 15.00 (14.00, 16.00) | >0.9 |
| **Peak Serum Creatinine** | 1.61 (1.20, 2.68) | 1.30 (1.01, 1.84) | <0.001 |
| **Blood PH:** | 7.42 (7.37, 7.46) | 7.43 (7.38, 7.46) | <0.001 |
| **Lung pO2 on 100%** | 199.00 (113.00, 410.00) | 134.05 (91.30, 301.00) | <0.001 |
| **Lung pO2 on Fio2 //If Yes, Lung pO2 on FiO2 of:** | 100.00 (50.00, 100.00) | 70.00 (40.00, 100.00) | <0.001 |
| **% Glomerulosclerosis (Right Kidney)** | 1.00 (1.00, 2.00) | 1.00 (1.00, 2.00) | <0.001 |
| **Final Resistance Prior to Shipping (right) //Final Resistance Prior to Shipping:** | 0.20 (0.14, 0.27) | 0.19 (0.14, 0.26) | 0.032 |
| **Serum Amylase** | 59.00 (33.00, 111.00) | 57.00 (35.00, 102.00) | >0.9 |
| **SGOT/AST** | 39.00 (22.00, 79.00) | 50.00 (32.00, 81.00) | <0.001 |
| **SGPT/ALT** | 38.00 (21.00, 78.00) | 39.50 (23.00, 75.00) | 0.034 |
| **Serum Sodium //Last Serum Sodium Prior to Procurement:** | 148.00 (143.00, 154.00) | 146.00 (141.00, 152.00) | <0.001 |
| **SVR (final) //SVR:** | 862.00 (692.00, 1,119.00) | 703.00 (703.00, 703.00) | 0.4 |
| **SVR (initial) //SVR: (dynes/sec/cm)^5)** | 904.00 (673.00, 1,133.00) | 647.00 (611.00, 982.00) | 0.3 |
| **Total Bilirubin** | 0.60 (0.40, 1.00) | 0.50 (0.40, 0.80) | <0.001 |
| **Troponin I** | 0.29 (0.05, 8.70) | 0.31 (0.03, 16.00) | 0.8 |
| **Troponin T** | 14.00 (0.08, 65.00) | 18.00 (1.15, 73.00) | 0.007 |
| **If Abnormal, # of Vessels with > 50% Stenosis** | 0.00 (0.00, 1.00) | 1.00 (0.00, 1.00) | 0.049 |
| **Estimated Warm Ischemic Time in minutes** | 0.00 (0.00, 0.00) | 20.00 (16.00, 24.00) | <0.001 |
| **Donor/s Weight in kilograms** | 80.60 (68.60, 95.00) | 81.00 (68.90, 95.30) | 0.5 |
| **Donor Risk Index (DRI)** | 1.65 (1.46, 1.96) | 2.41 (2.13, 2.82) | <0.001 |
| **Donor/s Blood Type** |  |  | <0.001 |
| A | 3,554 (16%) | 483 (16%) |  |
| A1 | 3,904 (17%) | 550 (19%) |  |
| A1B | 221 (1.0%) | 11 (0.4%) |  |
| A2 | 913 (4.1%) | 132 (4.5%) |  |
| A2B | 114 (0.5%) | 5 (0.2%) |  |
| AB | 326 (1.5%) | 15 (0.5%) |  |
| B | 2,539 (11%) | 268 (9.1%) |  |
| O | 10,873 (48%) | 1,466 (50%) |  |
| **CVA cause of death** |  |  | <0.001 |
| No | 16,700 (74%) | 2,403 (82%) |  |
| Yes | 5,744 (26%) | 527 (18%) |  |
| **history of hypertension** |  |  | <0.001 |
| No | 14,331 (64%) | 2,042 (70%) |  |
| Yes | 7,731 (34%) | 860 (29%) |  |
| Missing | 382 (1.7%) | 28 (1.0%) |  |
| **Allocate Remaining Liver Segment?** |  |  | 0.010 |
| Missing | 22,342 (100%) | 2,927 (100%) |  |
| No | 37 (0.2%) | 3 (0.1%) |  |
| Yes | 65 (0.3%) | 0 (0%) |  |
| **Anti-CMV** |  |  | <0.001 |
| Missing | 0 (0%) | 0 (0%) |  |
| Indeterminate | 72 (0.3%) | 8 (0.3%) |  |
| Negative | 7,868 (35%) | 1,234 (42%) |  |
| Not Done | 112 (0.5%) | 15 (0.5%) |  |
| Positive | 14,392 (64%) | 1,673 (57%) |  |
| **Anti-HBC** |  |  | 0.007 |
| Indeterminate | 2 (<0.1%) | 1 (<0.1%) |  |
| Negative | 21,262 (95%) | 2,821 (96%) |  |
| Not Done | 13 (<0.1%) | 1 (<0.1%) |  |
| Positive | 1,167 (5.2%) | 107 (3.7%) |  |
| **Anti-HCV** |  |  | 0.10 |
| Missing | 0 (0%) | 0 (0%) |  |
| Indeterminate | 4 (<0.1%) | 1 (<0.1%) |  |
| Negative | 20,208 (90%) | 2,686 (92%) |  |
| Not Done | 1 (<0.1%) | 0 (0%) |  |
| Positive | 2,231 (9.9%) | 243 (8.3%) |  |
| **Anti-HIV I/II:** |  |  | 0.005 |
| Negative | 18,981 (85%) | 2,424 (83%) |  |
| Not Done | 3,433 (15%) | 506 (17%) |  |
| Positive | 30 (0.1%) | 0 (0%) |  |
| **Anti-HTLV I/II:** |  |  | 0.037 |
| Indeterminate | 2 (<0.1%) | 0 (0%) |  |
| Negative | 4,290 (19%) | 625 (21%) |  |
| Not Done | 18,144 (81%) | 2,303 (79%) |  |
| Positive | 8 (<0.1%) | 2 (<0.1%) |  |
| **Arginine Vasopressin:** |  |  | <0.001 |
| Missing | 1 (<0.1%) | 0 (0%) |  |
| Negative / No | 7,042 (31%) | 2,205 (75%) |  |
| Positive / Yes | 15,391 (69%) | 722 (25%) |  |
| Unknown | 10 (<0.1%) | 3 (0.1%) |  |
| **Donor/s Cause of Death** |  |  | <0.001 |
| Anoxia | 10,256 (46%) | 1,548 (53%) |  |
| Cerebrovascular / Stroke | 5,808 (26%) | 530 (18%) |  |
| CNS Tumor | 78 (0.3%) | 3 (0.1%) |  |
| Head Trauma | 5,869 (26%) | 729 (25%) |  |
| Other | 433 (1.9%) | 120 (4.1%) |  |
| **Cardiac Arr. Since Event that led to Decl of Death** |  |  | <0.001 |
| Missing | 2 (<0.1%) | 2,893 (99%) |  |
| No | 20,976 (93%) | 32 (1.1%) |  |
| Yes | 1,466 (6.5%) | 5 (0.2%) |  |
| **Donor RNA Nat serology value for Chagas test** |  |  | 0.2 |
| Indeterminate | 3 (<0.1%) | 0 (0%) |  |
| Negative | 94 (0.4%) | 6 (0.2%) |  |
| Not Done | 22,346 (100%) | 2,924 (100%) |  |
| Positive | 1 (<0.1%) | 0 (0%) |  |
| **Chest X-ray:** |  |  | 0.025 |
| No chest x-ray | 15 (<0.1%) | 2 (<0.1%) |  |
| Normal | 3,727 (17%) | 414 (14%) |  |
| Abnormal-left | 1,927 (8.6%) | 228 (7.8%) |  |
| Abnormal-right | 2,031 (9.0%) | 262 (8.9%) |  |
| Abnormal-both | 13,481 (60%) | 1,861 (64%) |  |
| Results Unknown | 85 (0.4%) | 9 (0.3%) |  |
| Unknown if chest x-ray performed | 2 (<0.1%) | 0 (0%) |  |
| Missing | 1,176 (5.2%) | 154 (5.3%) |  |
| **Donor/s Citizenship** |  |  | <0.001 |
| US Citizen | 20,091 (90%) | 2,715 (93%) |  |
| Non-US Citizen/US Resident | 548 (2.4%) | 33 (1.1%) |  |
| Non-US Citizen/Non-US Resident | 109 (0.5%) | 10 (0.3%) |  |
| Unknown | 1,695 (7.6%) | 172 (5.9%) |  |
| Missing | 1 (<0.1%) | 0 (0%) |  |
| **Clamp Time Zone** |  |  | <0.001 |
| Eastern | 10,710 (48%) | 1,285 (44%) |  |
| Central | 7,131 (32%) | 920 (31%) |  |
| Mountain | 1,375 (6.1%) | 341 (12%) |  |
| Pacific | 2,852 (13%) | 384 (13%) |  |
| Alaska | 56 (0.2%) | 0 (0%) |  |
| Hawaii | 60 (0.3%) | 0 (0%) |  |
| Atlantic | 260 (1.2%) | 0 (0%) |  |
| **Clinical Infection** |  |  | 0.007 |
| Missing | 1 (<0.1%) | 0 (0%) |  |
| Negative / No | 4,746 (21%) | 710 (24%) |  |
| Positive / Yes | 17,668 (79%) | 2,217 (76%) |  |
| Unknown | 29 (0.1%) | 3 (0.1%) |  |
| **Donor cause of death is stroke (1= yes, 0= no)** |  |  | <0.001 |
| No | 16,578 (74%) | 2,391 (82%) |  |
| Yes | 5,866 (26%) | 539 (18%) |  |
| **Donor/s Continued Use in Last 6 Months** |  |  | 0.5 |
| Missing | 17,936 (80%) | 2,311 (79%) |  |
| Negative / No | 679 (3.0%) | 87 (3.0%) |  |
| Positive / Yes | 3,790 (17%) | 525 (18%) |  |
| Unknown | 39 (0.2%) | 7 (0.2%) |  |
| **Cocaine Use Last 6 Months** |  |  | 0.15 |
| Missing | 17,424 (78%) | 2,312 (79%) |  |
| Negative / No | 1,484 (6.6%) | 203 (6.9%) |  |
| Positive / Yes | 2,837 (13%) | 328 (11%) |  |
| Unknown | 699 (3.1%) | 87 (3.0%) |  |
| **Other Drug Abuse Last 6 Months** |  |  | 0.017 |
| Missing | 11,249 (50%) | 1,555 (53%) |  |
| Negative / No | 1,337 (6.0%) | 170 (5.8%) |  |
| Positive / Yes | 8,960 (40%) | 1,105 (38%) |  |
| Unknown | 898 (4.0%) | 100 (3.4%) |  |
| **Coronary Angiogram** |  |  | <0.001 |
| No | 15,247 (68%) | 2,782 (95%) |  |
| Yes, normal | 5,539 (25%) | 105 (3.6%) |  |
| Yes, not normal | 1,527 (6.8%) | 31 (1.1%) |  |
| 4 | 104 (0.5%) | 7 (0.2%) |  |
| 5 | 26 (0.1%) | 5 (0.2%) |  |
| Missing | 1 (<0.1%) | 0 (0%) |  |
| **If not DCD, Was this a consented DCD donor that progressed to brain death?** |  |  | <0.001 |
| Missing | 6 (<0.1%) | 2,930 (100%) |  |
| No | 19,142 (85%) | 0 (0%) |  |
| Yes | 3,296 (15%) | 0 (0%) |  |
| **Meds given Donor: DDAVP** |  |  | 0.8 |
| Missing | 1 (<0.1%) | 0 (0%) |  |
| Negative / No | 21,015 (94%) | 2,739 (93%) |  |
| Positive / Yes | 1,416 (6.3%) | 190 (6.5%) |  |
| Unknown | 12 (<0.1%) | 1 (<0.1%) |  |
| **Cirumstances of Death** |  |  | <0.001 |
| MVA | 2,751 (12%) | 416 (14%) |  |
| SUICIDE | 2,107 (9.4%) | 383 (13%) |  |
| HOMICIDE | 1,040 (4.6%) | 76 (2.6%) |  |
| CHILD-ABUSE | 5 (<0.1%) | 0 (0%) |  |
| NON-MVA | 4,303 (19%) | 549 (19%) |  |
| DEATH FROM NATURAL CAUSES | 9,438 (42%) | 1,225 (42%) |  |
| NONE OF THE ABOVE | 2,800 (12%) | 281 (9.6%) |  |
| **Mechanism of Death** |  |  | <0.001 |
| DROWNING | 94 (0.4%) | 12 (0.4%) |  |
| SEIZURE | 229 (1.0%) | 42 (1.4%) |  |
| DRUG INTOXICATION | 4,311 (19%) | 489 (17%) |  |
| ASPHYXIATION | 1,045 (4.7%) | 274 (9.4%) |  |
| CARDIOVASCULAR | 3,879 (17%) | 610 (21%) |  |
| ELECTRICAL | 19 (<0.1%) | 2 (<0.1%) |  |
| GUNSHOT WOUND | 2,109 (9.4%) | 202 (6.9%) |  |
| STAB | 44 (0.2%) | 3 (0.1%) |  |
| BLUNT INJURY | 3,750 (17%) | 541 (18%) |  |
| INTRACRANIAL HEMORRHAGE/STROKE | 5,887 (26%) | 543 (19%) |  |
| DEATH FROM NATURAL CAUSES | 733 (3.3%) | 150 (5.1%) |  |
| NONE OF THE ABOVE | 344 (1.5%) | 62 (2.1%) |  |
| **EBNA (Epstein-Barr nuclear antigen)** |  |  | <0.001 |
| Indeterminate | 26 (0.1%) | 4 (0.1%) |  |
| Negative | 387 (1.7%) | 79 (2.7%) |  |
| Not Done | 19,447 (87%) | 2,422 (83%) |  |
| Positive | 2,583 (12%) | 425 (15%) |  |
| Unknown | 1 (<0.1%) | 0 (0%) |  |
| **EBV//IgG:** |  |  | <0.001 |
| Indeterminate | 193 (0.9%) | 18 (0.6%) |  |
| Negative | 1,285 (5.7%) | 236 (8.1%) |  |
| Not Done | 598 (2.7%) | 121 (4.1%) |  |
| Positive | 20,368 (91%) | 2,555 (87%) |  |
| **EBV//IgM:** |  |  | 0.001 |
| Indeterminate | 54 (0.2%) | 8 (0.3%) |  |
| Negative | 17,968 (80%) | 2,250 (77%) |  |
| Not Done | 4,084 (18%) | 626 (21%) |  |
| Positive | 338 (1.5%) | 46 (1.6%) |  |
| **Method** |  |  | 0.003 |
| Echo | 17,913 (80%) | 1,820 (62%) |  |
| Angiogram | 211 (0.9%) | 8 (0.3%) |  |
| Missing | 4,320 (19%) | 1,102 (38%) |  |
| **SRTR Donor Ethnicity** |  |  | <0.001 |
| Latino | 3,540 (16%) | 302 (10%) |  |
| Non-Latino or unknown | 18,904 (84%) | 2,628 (90%) |  |
| **Does donor meet criteria to be an Expanded Donor?** |  |  | <0.001 |
| No | 17,211 (77%) | 2,531 (86%) |  |
| Yes | 5,185 (23%) | 398 (14%) |  |
| Missing | 48 (0.2%) | 1 (<0.1%) |  |
| **Meets expanded donor criteria for kidney (1= yes , 0= no)** |  |  | <0.001 |
| No | 17,217 (77%) | 2,526 (86%) |  |
| Yes | 5,227 (23%) | 404 (14%) |  |
| **Did the patient express to family or others the intent to be a donor** |  |  | <0.001 |
| Missing | 1,814 (8.1%) | 461 (16%) |  |
| Negative / No | 6,486 (29%) | 738 (25%) |  |
| Positive / Yes | 7,447 (33%) | 994 (34%) |  |
| Unknown | 6,697 (30%) | 737 (25%) |  |
| **Donor/s Gender** |  |  | <0.001 |
| Female | 8,650 (39%) | 943 (32%) |  |
| Male | 13,794 (61%) | 1,987 (68%) |  |
| **Hepatitis B Core Antibody Status** |  |  | 0.005 |
| Indeterminate | 2 (<0.1%) | 1 (<0.1%) |  |
| Negative | 21,269 (95%) | 2,818 (96%) |  |
| Not Done | 5 (<0.1%) | 2 (<0.1%) |  |
| Pending | 2 (<0.1%) | 2 (<0.1%) |  |
| Positive | 1,166 (5.2%) | 107 (3.7%) |  |
| **Donor RNA Nat serology value for hbv test** |  |  | 0.011 |
| Negative | 22,333 (100%) | 2,928 (100%) |  |
| Not Done | 3 (<0.1%) | 0 (0%) |  |
| Positive | 108 (0.5%) | 2 (<0.1%) |  |
| **HBsAb (Hepatitis B Surface Antibody)** |  |  | 0.5 |
| Indeterminate | 16 (<0.1%) | 3 (0.1%) |  |
| Negative | 1,249 (5.6%) | 170 (5.8%) |  |
| Not Done | 20,372 (91%) | 2,667 (91%) |  |
| Positive | 806 (3.6%) | 90 (3.1%) |  |
| Unknown | 1 (<0.1%) | 0 (0%) |  |
| **HBV//Surface Antigen:** |  |  | 0.5 |
| Negative | 22,391 (100%) | 2,925 (100%) |  |
| Not Done | 9 (<0.1%) | 2 (<0.1%) |  |
| Positive | 44 (0.2%) | 3 (0.1%) |  |
| **Donor RNA Nat serology value for hcv test** |  |  | 0.016 |
| Negative | 21,198 (94%) | 2,809 (96%) |  |
| Not Done | 1 (<0.1%) | 0 (0%) |  |
| Positive | 1,245 (5.5%) | 121 (4.1%) |  |
| **HCV Antibody Status** |  |  | 0.039 |
| Indeterminate | 3 (<0.1%) | 1 (<0.1%) |  |
| Negative | 20,203 (90%) | 2,684 (92%) |  |
| Not Done | 2 (<0.1%) | 1 (<0.1%) |  |
| Pending | 2 (<0.1%) | 1 (<0.1%) |  |
| Positive | 2,234 (10.0%) | 243 (8.3%) |  |
| **Heavy Alcohol Use (heavy= 2+ drinks/day):** |  |  | <0.001 |
| Negative / No | 17,657 (79%) | 2,246 (77%) |  |
| Positive / Yes | 3,876 (17%) | 586 (20%) |  |
| Unknown | 911 (4.1%) | 98 (3.3%) |  |
| **Heparin** |  |  | 0.3 |
| Missing | 1 (<0.1%) | 0 (0%) |  |
| Negative / No | 228 (1.0%) | 39 (1.3%) |  |
| Positive / Yes | 22,208 (99%) | 2,890 (99%) |  |
| Unknown | 7 (<0.1%) | 1 (<0.1%) |  |
| **Donor serum creatinine > 1.5 (1= yes, 0= no)** |  |  | <0.001 |
| No | 14,772 (66%) | 2,421 (83%) |  |
| Yes | 7,672 (34%) | 509 (17%) |  |
| **History of Cancer** |  |  | 0.2 |
| NO | 21,319 (95%) | 2,824 (96%) |  |
| CNS TUMOR - OTHER | 18 (<0.1%) | 4 (0.1%) |  |
| GENITOURINARY - BLADDER | 6 (<0.1%) | 2 (<0.1%) |  |
| GENITOURINARY - UTERINE CERVIX | 85 (0.4%) | 4 (0.1%) |  |
| GENITOURINARY - UTERINE BODY ENDOMETRIAL | 8 (<0.1%) | 0 (0%) |  |
| GENITOURINARY - UTERINE BODY CHORIOCARCINOMA | 1 (<0.1%) | 0 (0%) |  |
| GENITOURINARY - VULVA | 1 (<0.1%) | 0 (0%) |  |
| GENITOURINARY - OVARIAN | 27 (0.1%) | 2 (<0.1%) |  |
| GENITOURINARY - PENIS, TESTICULAR | 12 (<0.1%) | 1 (<0.1%) |  |
| SKIN - SQUAMOUS, BASAL CELL | 198 (0.9%) | 27 (0.9%) |  |
| GENITOURINARY - PROSTATE | 52 (0.2%) | 4 (0.1%) |  |
| GENITOURINARY - KIDNEY | 22 (<0.1%) | 3 (0.1%) |  |
| GENITOURINARY - UNKNOWN | 6 (<0.1%) | 1 (<0.1%) |  |
| GASTROINTESTINAL - ESOPHAGEAL | 2 (<0.1%) | 0 (0%) |  |
| GASTROINTESTINAL - STOMACH | 3 (<0.1%) | 0 (0%) |  |
| GASTROINTESTINAL - SMALL INTESTINE | 3 (<0.1%) | 0 (0%) |  |
| GASTROINTESTINAL - COLO-RECTAL | 14 (<0.1%) | 1 (<0.1%) |  |
| GASTROINTESTINAL - PANCREAS | 2 (<0.1%) | 1 (<0.1%) |  |
| BREAST | 73 (0.3%) | 7 (0.2%) |  |
| SKIN - MELANOMA | 20 (<0.1%) | 2 (<0.1%) |  |
| THYROID | 29 (0.1%) | 3 (0.1%) |  |
| TONGUE/THROAT | 7 (<0.1%) | 2 (<0.1%) |  |
| LARYNX | 1 (<0.1%) | 1 (<0.1%) |  |
| LUNG (include bronchial) | 4 (<0.1%) | 0 (0%) |  |
| LEUKEMIA/LYMPHOMA | 17 (<0.1%) | 5 (0.2%) |  |
| CNS TUMOR - ASTROCYTOMA | 9 (<0.1%) | 2 (<0.1%) |  |
| CNS TUMOR - GLIOBLASTOMA MULTIFORME | 4 (<0.1%) | 0 (0%) |  |
| CNS TUMOR - MEDULLOBLASTOMA | 3 (<0.1%) | 1 (<0.1%) |  |
| CNS TUMOR - NEUROBLASTOMA | 1 (<0.1%) | 0 (0%) |  |
| CNS TUMOR - MENINGIOMA | 9 (<0.1%) | 0 (0%) |  |
| UNKNOWN | 365 (1.6%) | 21 (0.7%) |  |
| OTHER, SPECIFY | 122 (0.5%) | 12 (0.4%) |  |
| Missing | 1 (<0.1%) | 0 (0%) |  |
| **Cigarette Use > 20 Pack Years - Ever** |  |  | <0.001 |
| Missing | 0 (0%) | 0 (0%) |  |
| Negative / No | 17,154 (76%) | 2,251 (77%) |  |
| Positive / Yes | 4,508 (20%) | 619 (21%) |  |
| Unknown | 782 (3.5%) | 60 (2.0%) |  |
| **Cocaine Use** |  |  | <0.001 |
| Missing | 1,816 (8.1%) | 460 (16%) |  |
| Negative / No | 15,043 (67%) | 1,802 (62%) |  |
| Positive / Yes | 5,020 (22%) | 618 (21%) |  |
| Unknown | 565 (2.5%) | 50 (1.7%) |  |
| **History of Diabetes** |  |  | <0.001 |
| No | 19,253 (86%) | 2,610 (89%) |  |
| Unknown | 370 (1.6%) | 26 (0.9%) |  |
| Yes, >10 years | 1,132 (5.0%) | 102 (3.5%) |  |
| Yes, 0-5 years | 813 (3.6%) | 111 (3.8%) |  |
| Yes, 6-10 years | 520 (2.3%) | 48 (1.6%) |  |
| Yes, Duration Unknown | 356 (1.6%) | 33 (1.1%) |  |
| **History of Hypertension** |  |  | <0.001 |
| No | 14,298 (64%) | 2,039 (70%) |  |
| Unknown | 381 (1.7%) | 29 (1.0%) |  |
| Yes, >10 years | 2,081 (9.3%) | 186 (6.3%) |  |
| Yes, 0-5 years | 3,066 (14%) | 400 (14%) |  |
| Yes, 6-10 years | 1,424 (6.3%) | 147 (5.0%) |  |
| Yes, Duration Unknown | 1,194 (5.3%) | 129 (4.4%) |  |
| **Insulin Dependent** |  |  | 0.10 |
| No | 1,182 (5.3%) | 146 (5.0%) |  |
| Unknown | 365 (1.6%) | 33 (1.1%) |  |
| Yes, >10 years | 535 (2.4%) | 50 (1.7%) |  |
| Yes, 0-5 years | 271 (1.2%) | 28 (1.0%) |  |
| Yes, 6-10 years | 172 (0.8%) | 18 (0.6%) |  |
| Yes, Duration Unknown | 296 (1.3%) | 19 (0.6%) |  |
| Missing | 19,623 (87%) | 2,636 (90%) |  |
| **Other Drug Abuse** |  |  | <0.001 |
| Missing | 1,815 (8.1%) | 460 (16%) |  |
| Negative / No | 9,057 (40%) | 1,068 (36%) |  |
| Positive / Yes | 11,195 (50%) | 1,375 (47%) |  |
| Unknown | 377 (1.7%) | 27 (0.9%) |  |
| **History of Previous MI** |  |  | 0.14 |
| Missing | 1 (<0.1%) | 0 (0%) |  |
| Negative / No | 20,901 (93%) | 2,756 (94%) |  |
| Positive / Yes | 1,088 (4.8%) | 136 (4.6%) |  |
| Unknown | 454 (2.0%) | 38 (1.3%) |  |
| **Donor RNA Nat serology value for hiv test** |  |  | 0.2 |
| Negative | 22,418 (100%) | 2,927 (100%) |  |
| Not Done | 5 (<0.1%) | 2 (<0.1%) |  |
| Positive | 21 (<0.1%) | 1 (<0.1%) |  |
| **Donor/s Home State** |  |  | <0.001 |
| ALASKA | 55 (0.2%) | 0 (0%) |  |
| ALABAMA | 537 (2.4%) | 41 (1.4%) |  |
| ARKANSAS | 227 (1.0%) | 47 (1.6%) |  |
| ARIZONA | 494 (2.2%) | 104 (3.5%) |  |
| CALIFORNIA | 1,889 (8.4%) | 229 (7.8%) |  |
| COLORADO | 318 (1.4%) | 97 (3.3%) |  |
| CONNECTICUT | 182 (0.8%) | 16 (0.5%) |  |
| DISTRICT OF COLUMBIA | 51 (0.2%) | 5 (0.2%) |  |
| DELAWARE | 140 (0.6%) | 4 (0.1%) |  |
| FLORIDA | 1,636 (7.3%) | 245 (8.4%) |  |
| GEORGIA | 815 (3.6%) | 80 (2.7%) |  |
| HAWAII | 52 (0.2%) | 1 (<0.1%) |  |
| IOWA | 177 (0.8%) | 30 (1.0%) |  |
| IDAHO | 102 (0.5%) | 24 (0.8%) |  |
| ILLINOIS | 796 (3.5%) | 107 (3.7%) |  |
| INDIANA | 600 (2.7%) | 103 (3.5%) |  |
| KANSAS | 265 (1.2%) | 28 (1.0%) |  |
| KENTUCKY | 379 (1.7%) | 57 (1.9%) |  |
| LOUISIANA | 499 (2.2%) | 57 (1.9%) |  |
| MASSACHUSETTS | 318 (1.4%) | 25 (0.9%) |  |
| MARYLAND | 412 (1.8%) | 41 (1.4%) |  |
| MAINE | 71 (0.3%) | 5 (0.2%) |  |
| MICHIGAN | 667 (3.0%) | 102 (3.5%) |  |
| MINNESOTA | 257 (1.1%) | 38 (1.3%) |  |
| MISSOURI | 657 (2.9%) | 61 (2.1%) |  |
| MISSISSIPPI | 299 (1.3%) | 29 (1.0%) |  |
| MONTANA | 69 (0.3%) | 12 (0.4%) |  |
| FOREIGN COUNTRY | 6 (<0.1%) | 1 (<0.1%) |  |
| NORTH CAROLINA | 731 (3.3%) | 113 (3.9%) |  |
| NORTH DAKOTA | 42 (0.2%) | 9 (0.3%) |  |
| NEBRASKA | 108 (0.5%) | 32 (1.1%) |  |
| NEW HAMPSHIRE | 60 (0.3%) | 3 (0.1%) |  |
| NEW JERSEY | 501 (2.2%) | 42 (1.4%) |  |
| NEW MEXICO | 136 (0.6%) | 23 (0.8%) |  |
| NEVADA | 279 (1.2%) | 45 (1.5%) |  |
| NEW YORK | 975 (4.3%) | 91 (3.1%) |  |
| OHIO | 943 (4.2%) | 179 (6.1%) |  |
| OKLAHOMA | 304 (1.4%) | 58 (2.0%) |  |
| OREGON | 216 (1.0%) | 36 (1.2%) |  |
| PENNSYLVANIA | 1,044 (4.7%) | 95 (3.2%) |  |
| PUERTO RICO | 252 (1.1%) | 1 (<0.1%) |  |
| RHODE ISLAND | 39 (0.2%) | 4 (0.1%) |  |
| SOUTH CAROLINA | 365 (1.6%) | 41 (1.4%) |  |
| SOUTH DAKOTA | 25 (0.1%) | 5 (0.2%) |  |
| TENNESSEE | 725 (3.2%) | 72 (2.5%) |  |
| TEXAS | 1,852 (8.3%) | 234 (8.0%) |  |
| UTAH | 168 (0.7%) | 62 (2.1%) |  |
| VIRGINIA | 491 (2.2%) | 35 (1.2%) |  |
| VIRGIN ISLANDS | 2 (<0.1%) | 0 (0%) |  |
| VERMONT | 31 (0.1%) | 4 (0.1%) |  |
| WASHINGTON | 427 (1.9%) | 59 (2.0%) |  |
| WISCONSIN | 423 (1.9%) | 59 (2.0%) |  |
| WEST VIRGINIA | 184 (0.8%) | 17 (0.6%) |  |
| WYOMING | 32 (0.1%) | 11 (0.4%) |  |
| UNKNOWN | 119 (0.5%) | 11 (0.4%) |  |
| **Donor RNA Nat serology value for htlv test** |  |  | 0.6 |
| Indeterminate | 4 (<0.1%) | 0 (0%) |  |
| Negative | 29 (0.1%) | 2 (<0.1%) |  |
| Not Done | 22,409 (100%) | 2,928 (100%) |  |
| Positive | 1 (<0.1%) | 0 (0%) |  |
| Unknown | 1 (<0.1%) | 0 (0%) |  |
| **Donor history of hypertension (1= yes, 0= no)** |  |  | <0.001 |
| No | 14,275 (64%) | 2,037 (70%) |  |
| Yes | 7,795 (35%) | 865 (30%) |  |
| Missing | 374 (1.7%) | 28 (1.0%) |  |
| **Infection Source: Blood** |  |  | 0.007 |
| No | 19,454 (87%) | 2,488 (85%) |  |
| Yes | 2,990 (13%) | 442 (15%) |  |
| **Infection Source: Lung** |  |  | <0.001 |
| No | 6,719 (30%) | 1,027 (35%) |  |
| Yes | 15,725 (70%) | 1,903 (65%) |  |
| **Infection Source: Other** |  |  | 0.061 |
| No | 20,722 (92%) | 2,676 (91%) |  |
| Yes | 1,722 (7.7%) | 254 (8.7%) |  |
| **Infection Source: Urine** |  |  | 0.4 |
| No | 19,133 (85%) | 2,481 (85%) |  |
| Yes | 3,311 (15%) | 449 (15%) |  |
| **Inotropic Support** |  |  | <0.001 |
| Missing | 1 (<0.1%) | 0 (0%) |  |
| Negative / No | 12,805 (57%) | 2,799 (96%) |  |
| Positive / Yes | 9,587 (43%) | 130 (4.4%) |  |
| Unknown | 51 (0.2%) | 1 (<0.1%) |  |
| **Insulin:** |  |  | <0.001 |
| Missing | 1 (<0.1%) | 0 (0%) |  |
| Negative / No | 10,622 (47%) | 2,399 (82%) |  |
| Positive / Yes | 11,805 (53%) | 530 (18%) |  |
| Unknown | 16 (<0.1%) | 1 (<0.1%) |  |
| **Left Lung Machine Perfusion Intended or Performed:** |  |  | <0.001 |
| Missing | 12,713 (57%) | 2,099 (72%) |  |
| No | 9,310 (41%) | 736 (25%) |  |
| Yes | 421 (1.9%) | 95 (3.2%) |  |
| **Was the patient declared legally brain dead:** |  |  | <0.001 |
| Missing | 1 (<0.1%) | 0 (0%) |  |
| No | 1 (<0.1%) | 2,887 (99%) |  |
| Yes | 22,442 (100%) | 43 (1.5%) |  |
| **Kidney Biopsy (left)** |  |  | <0.001 |
| Missing | 483 (2.2%) | 33 (1.1%) |  |
| No | 11,561 (52%) | 1,374 (47%) |  |
| Yes | 10,400 (46%) | 1,523 (52%) |  |
| **Pump (Left Kidney)** |  |  | <0.001 |
| Missing | 498 (2.2%) | 34 (1.2%) |  |
| No | 14,799 (66%) | 1,116 (38%) |  |
| Yes | 7,147 (32%) | 1,780 (61%) |  |
| **Transferred on pump (left)://Transferred on pump:** |  |  | <0.001 |
| Missing | 15,297 (68%) | 1,150 (39%) |  |
| No | 2,785 (12%) | 615 (21%) |  |
| Yes | 4,362 (19%) | 1,165 (40%) |  |
| **Left Lung Bronchoscopy:** |  |  | <0.001 |
| No Bronchoscopy | 756 (3.4%) | 258 (8.8%) |  |
| Bronchoscopy Results normal | 6,021 (27%) | 249 (8.5%) |  |
| Bronchoscopy Results, Abnormal-purulent secretions | 1,087 (4.8%) | 62 (2.1%) |  |
| Bronchoscopy Results, Abnormal-aspiration of foreign body | 65 (0.3%) | 3 (0.1%) |  |
| Bronchoscopy Results, Abnormal-blood | 397 (1.8%) | 16 (0.5%) |  |
| Bronchoscopy Results, Abnormal-anatomy/other lesion | 121 (0.5%) | 9 (0.3%) |  |
| Bronchoscopy Results, Unknown | 13 (<0.1%) | 2 (<0.1%) |  |
| 8 | 35 (0.2%) | 8 (0.3%) |  |
| Missing | 13,949 (62%) | 2,323 (79%) |  |
| **Liver Biopsy** |  |  | <0.001 |
| Missing | 2 (<0.1%) | 0 (0%) |  |
| No | 12,334 (55%) | 2,010 (69%) |  |
| Yes | 10,108 (45%) | 920 (31%) |  |
| **Medical Examiner/Coroner** |  |  | 0.011 |
| NO | 6,397 (29%) | 741 (25%) |  |
| YES, MEDICAL EXAMINER CONSENTED | 14,177 (63%) | 1,706 (58%) |  |
| YES, MEDICAL EXAMINER REFUSED CONSENT | 41 (0.2%) | 3 (0.1%) |  |
| UNKNOWN | 104 (0.5%) | 25 (0.9%) |  |
| Missing | 1,725 (7.7%) | 455 (16%) |  |
| **Does the Donor meet CDC guidelines for High Risk for an organ donor:** |  |  | 0.001 |
| Missing | 0 (0%) | 0 (0%) |  |
| Negative / No | 17,041 (76%) | 2,317 (79%) |  |
| Positive / Yes | 5,403 (24%) | 613 (21%) |  |
| **Non-Heart Beating Donor** |  |  | <0.001 |
| Missing | 0 (0%) | 0 (0%) |  |
| No | 22,439 (100%) | 0 (0%) |  |
| Yes | 5 (<0.1%) | 2,930 (100%) |  |
| **Controlled** |  |  | <0.001 |
| Missing | 22,440 (100%) | 0 (0%) |  |
| Negative / No | 4 (<0.1%) | 0 (0%) |  |
| Positive / Yes | 0 (0%) | 2,930 (100%) |  |
| **Core Cooling Used** |  |  | <0.001 |
| Missing | 20,408 (91%) | 0 (0%) |  |
| No | 767 (3.4%) | 442 (15%) |  |
| Yes | 1,269 (5.7%) | 2,488 (85%) |  |
| **Organ was used in a shared 1 (vs local 0) TX** |  |  | <0.001 |
| No | 8,399 (37%) | 1,539 (53%) |  |
| Yes | 14,045 (63%) | 1,391 (47%) |  |
| **Pre-Recov Meds given Donor: Diuretics** |  |  | <0.001 |
| Missing | 1 (<0.1%) | 0 (0%) |  |
| Negative / No | 8,319 (37%) | 2,363 (81%) |  |
| Positive / Yes | 14,117 (63%) | 566 (19%) |  |
| Unknown | 7 (<0.1%) | 1 (<0.1%) |  |
| **Pre-Recov Meds given Donor: Steroids** |  |  | <0.001 |
| Missing | 1 (<0.1%) | 0 (0%) |  |
| Negative / No | 6,233 (28%) | 2,343 (80%) |  |
| Positive / Yes | 16,182 (72%) | 584 (20%) |  |
| Unknown | 28 (0.1%) | 3 (0.1%) |  |
| **Pre-Recov Meds given Donor: T3** |  |  | 0.2 |
| Missing | 1 (<0.1%) | 0 (0%) |  |
| Negative / No | 22,384 (100%) | 2,929 (100%) |  |
| Positive / Yes | 45 (0.2%) | 0 (0%) |  |
| Unknown | 14 (<0.1%) | 1 (<0.1%) |  |
| **Pre-Recov Meds given Donor: T4** |  |  | <0.001 |
| Missing | 1 (<0.1%) | 0 (0%) |  |
| Negative / No | 10,708 (48%) | 2,719 (93%) |  |
| Positive / Yes | 11,722 (52%) | 210 (7.2%) |  |
| Unknown | 13 (<0.1%) | 1 (<0.1%) |  |
| **Previous Gastrointestinal Disease** |  |  | 0.049 |
| Missing | 16 (<0.1%) | 1 (<0.1%) |  |
| Negative / No | 20,753 (92%) | 2,732 (93%) |  |
| Positive / Yes | 1,331 (5.9%) | 171 (5.8%) |  |
| Unknown | 344 (1.5%) | 26 (0.9%) |  |
| **Protein in Urine** |  |  | 0.2 |
| Missing | 1 (<0.1%) | 0 (0%) |  |
| Negative / No | 9,015 (40%) | 1,121 (38%) |  |
| Positive / Yes | 13,183 (59%) | 1,774 (61%) |  |
| Unknown | 245 (1.1%) | 35 (1.2%) |  |
| **Was a pulmonary artery catheter placed:** |  |  | <0.001 |
| Missing | 1,818 (8.1%) | 460 (16%) |  |
| No | 20,059 (89%) | 2,454 (84%) |  |
| Yes | 567 (2.5%) | 16 (0.5%) |  |
| **SRTR Donor Race** |  |  | <0.001 |
| Asian | 579 (2.6%) | 58 (2.0%) |  |
| Black | 4,387 (20%) | 311 (11%) |  |
| Multiracial | 87 (0.4%) | 11 (0.3%) |  |
| Native American | 160 (0.7%) | 21 (0.7%) |  |
| Pacific Islander | 64 (0.3%) | 7 (0.2%) |  |
| White | 17,167 (76%) | 2,522 (86%) |  |
| **Duration of Resuscitation** |  |  | 0.8 |
| 1 | 19 (<0.1%) | 0 (0%) |  |
| 10 | 89 (0.4%) | 0 (0%) |  |
| 11 | 15 (<0.1%) | 0 (0%) |  |
| 12 | 28 (0.1%) | 0 (0%) |  |
| 120 | 1 (<0.1%) | 0 (0%) |  |
| 13 | 9 (<0.1%) | 0 (0%) |  |
| 14 | 13 (<0.1%) | 0 (0%) |  |
| 15 | 68 (0.3%) | 0 (0%) |  |
| 16 | 12 (<0.1%) | 0 (0%) |  |
| 17 | 6 (<0.1%) | 0 (0%) |  |
| 18 | 11 (<0.1%) | 0 (0%) |  |
| 19 | 6 (<0.1%) | 0 (0%) |  |
| 2 | 59 (0.3%) | 0 (0%) |  |
| 20 | 90 (0.4%) | 0 (0%) |  |
| 21 | 11 (<0.1%) | 0 (0%) |  |
| 22 | 13 (<0.1%) | 0 (0%) |  |
| 23 | 11 (<0.1%) | 0 (0%) |  |
| 24 | 6 (<0.1%) | 0 (0%) |  |
| 25 | 37 (0.2%) | 1 (<0.1%) |  |
| 26 | 8 (<0.1%) | 0 (0%) |  |
| 27 | 3 (<0.1%) | 0 (0%) |  |
| 28 | 4 (<0.1%) | 0 (0%) |  |
| 29 | 8 (<0.1%) | 0 (0%) |  |
| 3 | 46 (0.2%) | 0 (0%) |  |
| 30 | 83 (0.4%) | 2 (<0.1%) |  |
| 31 | 2 (<0.1%) | 0 (0%) |  |
| 32 | 3 (<0.1%) | 0 (0%) |  |
| 33 | 4 (<0.1%) | 0 (0%) |  |
| 34 | 4 (<0.1%) | 0 (0%) |  |
| 35 | 21 (<0.1%) | 0 (0%) |  |
| 36 | 8 (<0.1%) | 0 (0%) |  |
| 37 | 7 (<0.1%) | 0 (0%) |  |
| 38 | 1 (<0.1%) | 0 (0%) |  |
| 39 | 1 (<0.1%) | 0 (0%) |  |
| 4 | 44 (0.2%) | 0 (0%) |  |
| 40 | 32 (0.1%) | 0 (0%) |  |
| 41 | 2 (<0.1%) | 0 (0%) |  |
| 42 | 2 (<0.1%) | 0 (0%) |  |
| 43 | 5 (<0.1%) | 0 (0%) |  |
| 44 | 6 (<0.1%) | 0 (0%) |  |
| 45 | 30 (0.1%) | 0 (0%) |  |
| 46 | 1 (<0.1%) | 0 (0%) |  |
| 47 | 1 (<0.1%) | 0 (0%) |  |
| 48 | 3 (<0.1%) | 0 (0%) |  |
| 49 | 2 (<0.1%) | 0 (0%) |  |
| 5 | 49 (0.2%) | 0 (0%) |  |
| 50 | 20 (<0.1%) | 0 (0%) |  |
| 52 | 1 (<0.1%) | 0 (0%) |  |
| 54 | 1 (<0.1%) | 0 (0%) |  |
| 55 | 5 (<0.1%) | 0 (0%) |  |
| 59 | 1 (<0.1%) | 0 (0%) |  |
| 6 | 40 (0.2%) | 0 (0%) |  |
| 60 | 32 (0.1%) | 0 (0%) |  |
| 62 | 2 (<0.1%) | 0 (0%) |  |
| 63 | 1 (<0.1%) | 0 (0%) |  |
| 65 | 1 (<0.1%) | 0 (0%) |  |
| 68 | 1 (<0.1%) | 0 (0%) |  |
| 69 | 1 (<0.1%) | 0 (0%) |  |
| 7 | 14 (<0.1%) | 0 (0%) |  |
| 70 | 6 (<0.1%) | 0 (0%) |  |
| 71 | 1 (<0.1%) | 0 (0%) |  |
| 75 | 2 (<0.1%) | 0 (0%) |  |
| 77 | 1 (<0.1%) | 0 (0%) |  |
| 8 | 29 (0.1%) | 1 (<0.1%) |  |
| 86 | 1 (<0.1%) | 0 (0%) |  |
| 88 | 2 (<0.1%) | 0 (0%) |  |
| 89 | 1 (<0.1%) | 0 (0%) |  |
| 9 | 21 (<0.1%) | 0 (0%) |  |
| 90 | 3 (<0.1%) | 0 (0%) |  |
| 99 | 1 (<0.1%) | 0 (0%) |  |
| Missing | 21,372 (95%) | 2,926 (100%) |  |
| **Right Lung Machine Perfusion Intended or Performed** |  |  | <0.001 |
| Missing | 12,795 (57%) | 2,096 (72%) |  |
| No | 9,228 (41%) | 739 (25%) |  |
| Yes | 421 (1.9%) | 95 (3.2%) |  |
| **RPR-VDRL** |  |  | 0.7 |
| Indeterminate | 1 (<0.1%) | 0 (0%) |  |
| Negative | 22,065 (98%) | 2,887 (99%) |  |
| Not Done | 27 (0.1%) | 4 (0.1%) |  |
| Positive | 351 (1.6%) | 39 (1.3%) |  |
| **Kidney Biopsy (right)** |  |  | <0.001 |
| Missing | 492 (2.2%) | 33 (1.1%) |  |
| No | 11,350 (51%) | 1,347 (46%) |  |
| Yes | 10,602 (47%) | 1,550 (53%) |  |
| **Pump (Right Kidney)** |  |  | <0.001 |
| Missing | 509 (2.3%) | 33 (1.1%) |  |
| No | 14,597 (65%) | 1,068 (36%) |  |
| Yes | 7,338 (33%) | 1,829 (62%) |  |
| **Transferred on pump (right)://Transferred on pump:** |  |  | <0.001 |
| Missing | 15,106 (67%) | 1,101 (38%) |  |
| No | 2,892 (13%) | 650 (22%) |  |
| Yes | 4,446 (20%) | 1,179 (40%) |  |
| **Right Lung Bronchoscopy:** |  |  | <0.001 |
| No Bronchoscopy | 765 (3.4%) | 260 (8.9%) |  |
| Bronchoscopy Results normal | 5,765 (26%) | 247 (8.4%) |  |
| Bronchoscopy Results, Abnormal-purulent secretions | 1,239 (5.5%) | 67 (2.3%) |  |
| Bronchoscopy Results, Abnormal-aspiration of foreign body | 91 (0.4%) | 4 (0.1%) |  |
| Bronchoscopy Results, Abnormal-blood | 434 (1.9%) | 14 (0.5%) |  |
| Bronchoscopy Results, Abnormal-anatomy/other lesion | 133 (0.6%) | 9 (0.3%) |  |
| Bronchoscopy Results, Unknown | 14 (<0.1%) | 2 (<0.1%) |  |
| 8 | 37 (0.2%) | 10 (0.3%) |  |
| Unknown if bronchoscopy performed | 1 (<0.1%) | 0 (0%) |  |
| Missing | 13,965 (62%) | 2,317 (79%) |  |
| **strongyloides test result selection** |  |  | <0.001 |
| Missing | 2,959 (13%) | 279 (9.5%) |  |
| Indeterminate | 9 (<0.1%) | 2 (<0.1%) |  |
| Negative | 3,575 (16%) | 416 (14%) |  |
| Not Done | 15,695 (70%) | 2,216 (76%) |  |
| Pending | 88 (0.4%) | 5 (0.2%) |  |
| Positive | 118 (0.5%) | 12 (0.4%) |  |
| **Lifestyle Factors: Tattoos** |  |  | <0.001 |
| Missing | 1,815 (8.1%) | 460 (16%) |  |
| Negative / No | 9,433 (42%) | 1,069 (36%) |  |
| Positive / Yes | 11,161 (50%) | 1,394 (48%) |  |
| Unknown | 35 (0.2%) | 7 (0.2%) |  |
| **Number of transfusions during this (terminal) hospitalization:** |  |  | <0.001 |
| NONE | 13,626 (61%) | 2,002 (68%) |  |
| 1 - 5 | 6,453 (29%) | 701 (24%) |  |
| 6 - 10 | 1,478 (6.6%) | 129 (4.4%) |  |
| GREATER THAN 10 | 844 (3.8%) | 72 (2.5%) |  |
| UNKNOWN | 5 (<0.1%) | 0 (0%) |  |
| Missing | 38 (0.2%) | 26 (0.9%) |  |
| **Donor/s HLA - Typing Method Class I** |  |  | 0.029 |
| Serology | 11 (<0.1%) | 0 (0%) |  |
| DNA | 21,850 (97%) | 2,873 (98%) |  |
| 3 | 263 (1.2%) | 20 (0.7%) |  |
| Missing | 320 (1.4%) | 37 (1.3%) |  |
| **Donor/s HLA - Typing Method Class II** |  |  | 0.034 |
| Serology | 20 (<0.1%) | 2 (<0.1%) |  |
| DNA | 21,870 (97%) | 2,875 (98%) |  |
| 3 | 234 (1.0%) | 16 (0.5%) |  |
| Missing | 320 (1.4%) | 37 (1.3%) |  |
| **Vasodilators** |  |  | <0.001 |
| Missing | 1 (<0.1%) | 0 (0%) |  |
| Negative / No | 18,214 (81%) | 2,548 (87%) |  |
| Positive / Yes | 4,218 (19%) | 379 (13%) |  |
| Unknown | 11 (<0.1%) | 3 (0.1%) |  |
| **Wall Abnormalities //Global:** |  |  | 0.6 |
| Missing | 20,137 (90%) | 2,645 (90%) |  |
| No | 1,368 (6.1%) | 170 (5.8%) |  |
| Yes | 939 (4.2%) | 115 (3.9%) |  |
| **Wall Abnormalities //Segmental:** |  |  | 0.5 |
| Missing | 20,138 (90%) | 2,645 (90%) |  |
| No | 1,638 (7.3%) | 196 (6.7%) |  |
| Yes | 668 (3.0%) | 89 (3.0%) |  |
| **Donor RNA Nat serology value for West Nile test** |  |  | <0.001 |
| Indeterminate | 2 (<0.1%) | 1 (<0.1%) |  |
| Negative | 8,065 (36%) | 1,197 (41%) |  |
| Not Done | 14,377 (64%) | 1,732 (59%) |  |
| **^1^Median (Q1, Q3); n (%)** | | | |
| **^2^Wilcoxon rank sum test; Pearson's Chi-squared test with simulated p-value (based on 2000 replicates)** | | | |

| **Table D4. Graft failure causes by donor type among recipients with graft failure** | | | |
| --- | --- | --- | --- |
| **Characteristic** | **DBD**  N = 2,966^1^ | **DCD**  N = 146^1^ | **p-value**^2^ |
| Biliary Tract Complication |  |  | <0.001 |
| No | 2,650 (89%) | 117 (80%) |  |
| Yes | 316 (11%) | 29 (20%) |  |
| Hepatitis: DeNovo |  |  | 0.7 |
| No | 2,928 (99%) | 145 (99%) |  |
| Yes | 38 (1.3%) | 1 (0.7%) |  |
| Hepatitis: Recurrent |  |  | <0.001 |
| No | 2,060 (69%) | 124 (85%) |  |
| Yes | 906 (31%) | 22 (15%) |  |
| Infection |  |  | 0.034 |
| No | 2,348 (79%) | 105 (72%) |  |
| Yes | 618 (21%) | 41 (28%) |  |
| Primary Graft Failure |  |  | 0.040 |
| No | 2,517 (85%) | 133 (91%) |  |
| Yes | 449 (15%) | 13 (8.9%) |  |
| Patient Noncompliance |  |  | 0.10 |
| No | 2,831 (95%) | 144 (99%) |  |
| Yes | 135 (4.6%) | 2 (1.4%) |  |
| Recurrent Disease: non-Hepatitis |  |  | 0.6 |
| No | 2,068 (70%) | 105 (72%) |  |
| Yes | 898 (30%) | 41 (28%) |  |
| Acute Rejection |  |  | 0.8 |
| No | 2,549 (86%) | 124 (85%) |  |
| Yes | 417 (14%) | 22 (15%) |  |
| Chronic Rejection |  |  | 0.6 |
| No | 2,295 (77%) | 116 (79%) |  |
| Yes | 671 (23%) | 30 (21%) |  |
| Vascular Thrombosis |  |  | 0.11 |
| No | 2,737 (92%) | 140 (96%) |  |
| Yes | 229 (7.7%) | 6 (4.1%) |  |
| ^1^n (%) | | | |
| ^2^Pearson's Chi-squared test with simulated p-value | | | |
